# Supplementary material for: Gas chromatography (GC) fingerprinting and immunomodulatory activity of polysaccharide from the rhizome of Menispermum dauricum DC
Source: PeerJ. 2022 Aug 22;10:e13946. doi: 10.7717/peerj.13946 (PMC9406803; doi:10.7717/peerj.13946)
Supplement: Supplemental Information 1 — 1. GlcUA; 2. Gal; 3. Glc; 4. Man; 5. The total polysaccharides from the rhizome of M. dauricum (tMDP); 6. Ara; 7. Fuc; 8. Xyl; 9. Rha; 10. Fru [file peerj-10-13946-s001.docx]

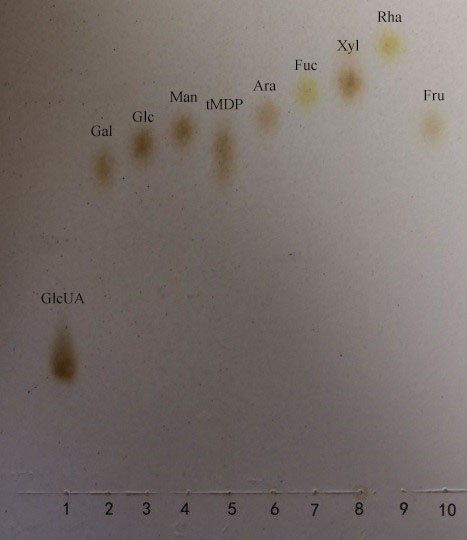


Fig. S1 TLC of the total polysaccharides from the rhizome of *M. dauricum*. 1. GlcUA；2. Gal；3. Glc；4. Man；5. The total polysaccharides from the rhizome of *M. dauricum* (tMDP)；6. Ara；7. Fuc；8. Xyl；9. Rha；10. Fru
