# Supplementary figures and images for: Gas chromatography (GC) fingerprinting and immunomodulatory activity of polysaccharide from the rhizome of Menispermum dauricum DC (part 1 of 2)
Source: PeerJ. 2022 Aug 22;10:e13946. doi: 10.7717/peerj.13946 (PMC9406803; doi:10.7717/peerj.13946)

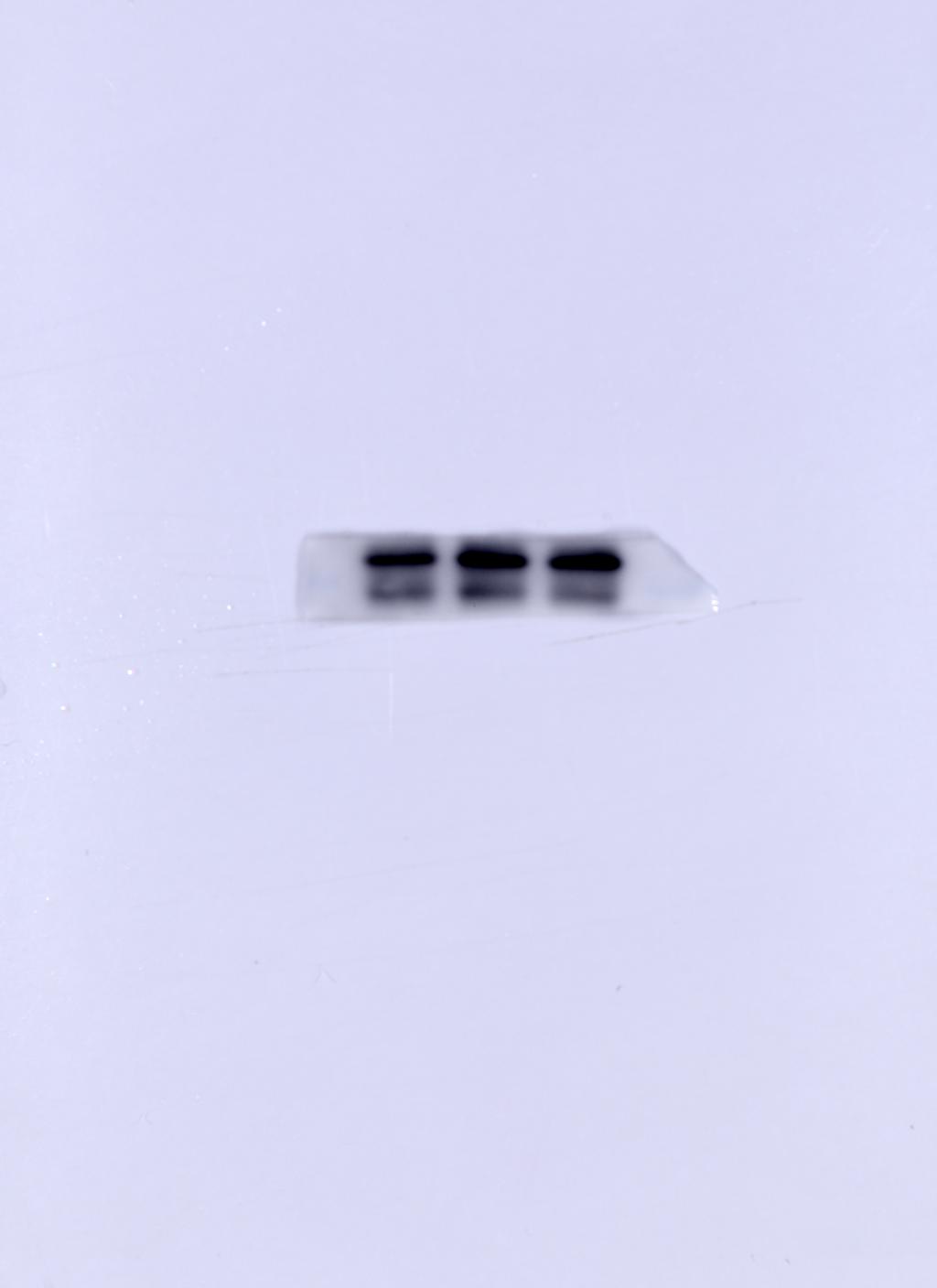

Supplement: Supplemental Information 2 — The CDF files for Fig. 2 are the data of analyzing the fingerprints of 10 batches of the rhizome of M. dauricum Polysaccharide. The software to open this file is the Similarity Evaluation System for Chromatographic Fingerprint of Traditional Chinese Medicine, and the software installation package is available in the Figure 2 folder. The OPJU file in the Figure 3 folder is the data of PCA analysis, and the open software is Origin, which can be downloaded and used for a fee from the Origin official website. [file peerj-10-13946-s002.zip › Raw date/Fig.6/MyD88/1/MyD88+Marker.jpg]

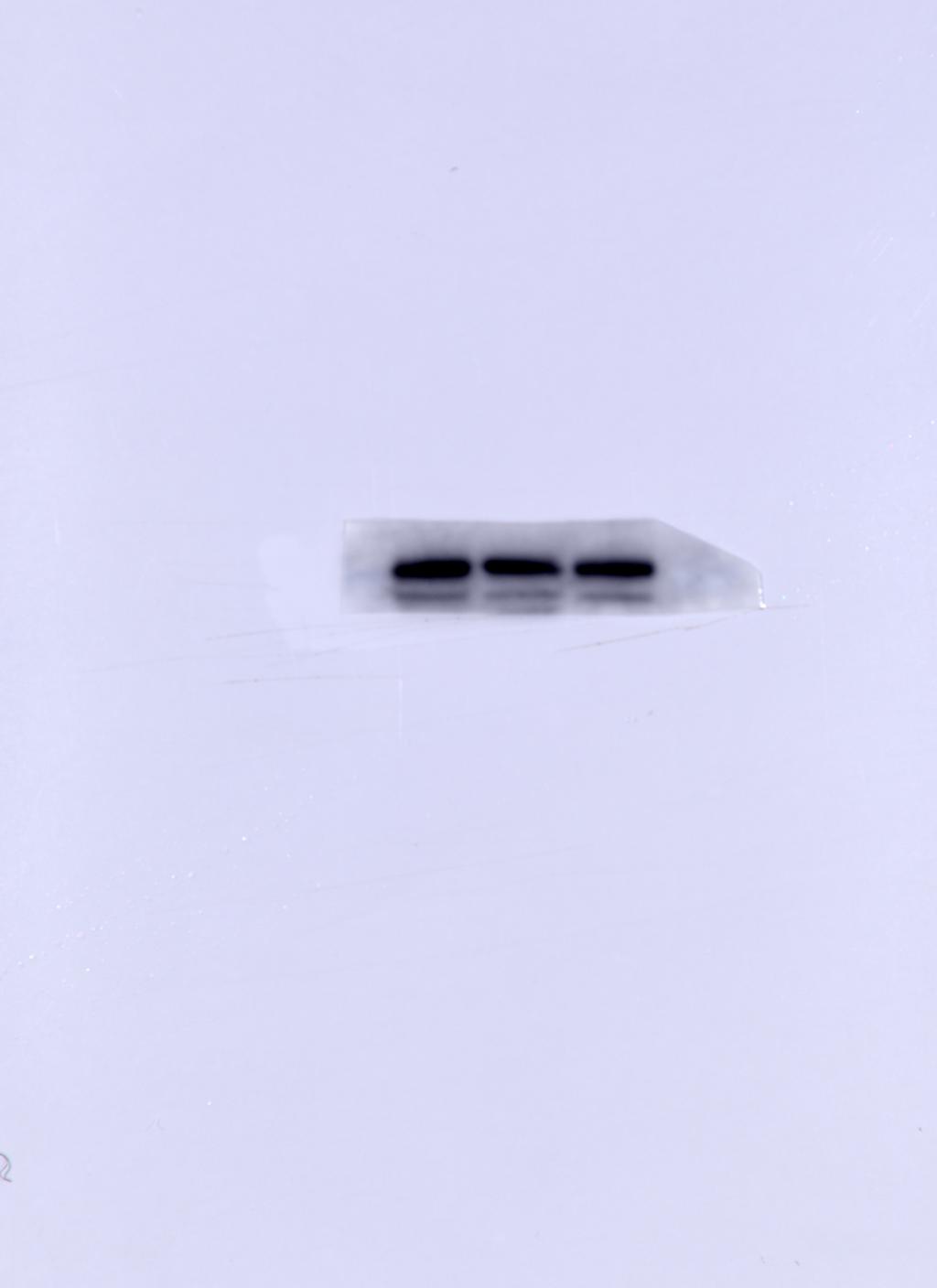

Supplement: Supplemental Information 2 — The CDF files for Fig. 2 are the data of analyzing the fingerprints of 10 batches of the rhizome of M. dauricum Polysaccharide. The software to open this file is the Similarity Evaluation System for Chromatographic Fingerprint of Traditional Chinese Medicine, and the software installation package is available in the Figure 2 folder. The OPJU file in the Figure 3 folder is the data of PCA analysis, and the open software is Origin, which can be downloaded and used for a fee from the Origin official website. [file peerj-10-13946-s002.zip › Raw date/Fig.6/MyD88/1/MyD88-GAPDH+Marker.jpg]

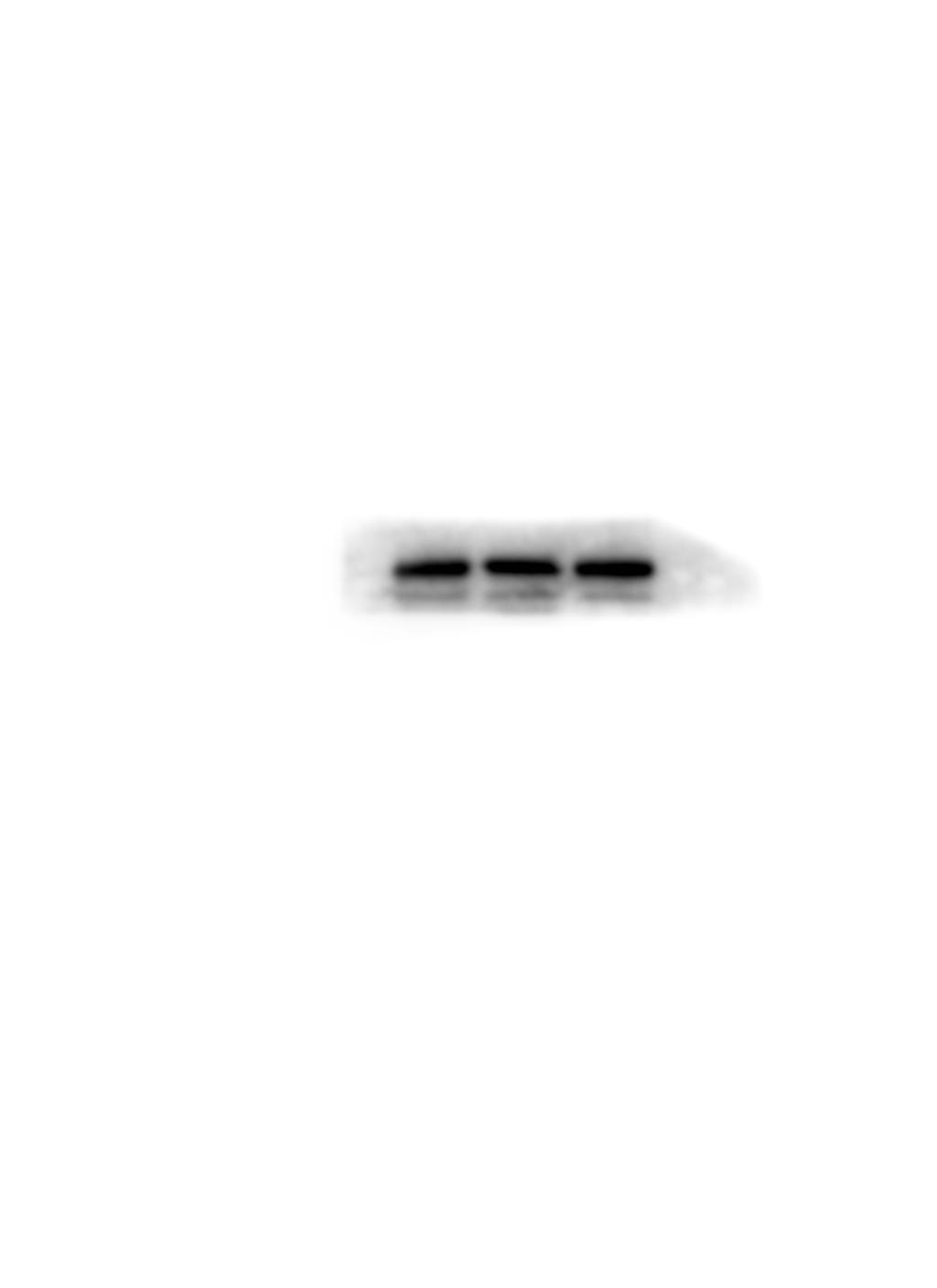

Supplement: Supplemental Information 2 — The CDF files for Fig. 2 are the data of analyzing the fingerprints of 10 batches of the rhizome of M. dauricum Polysaccharide. The software to open this file is the Similarity Evaluation System for Chromatographic Fingerprint of Traditional Chinese Medicine, and the software installation package is available in the Figure 2 folder. The OPJU file in the Figure 3 folder is the data of PCA analysis, and the open software is Origin, which can be downloaded and used for a fee from the Origin official website. [file peerj-10-13946-s002.zip › Raw date/Fig.6/MyD88/1/MyD88-GAPDH.jpg]

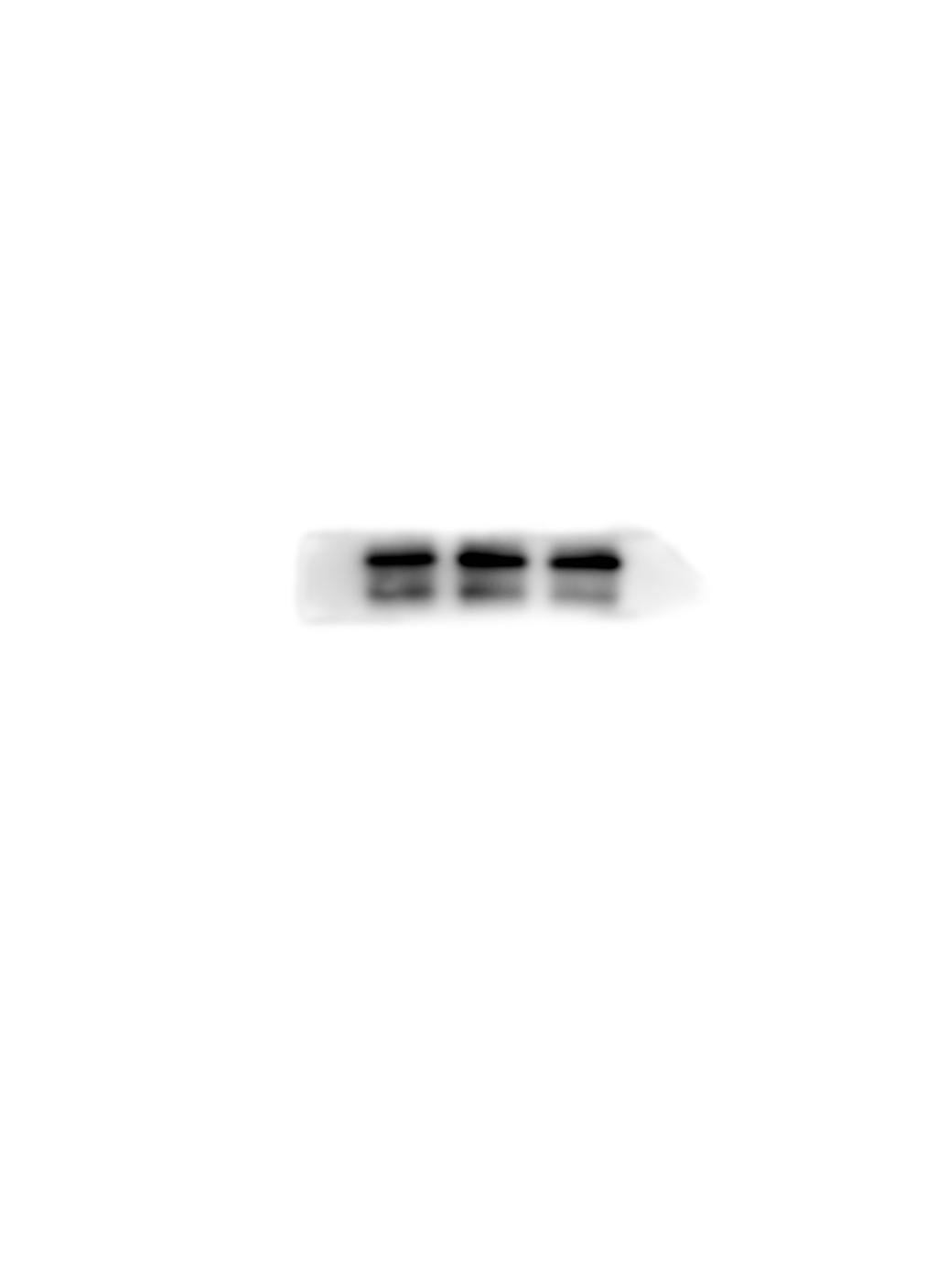

Supplement: Supplemental Information 2 — The CDF files for Fig. 2 are the data of analyzing the fingerprints of 10 batches of the rhizome of M. dauricum Polysaccharide. The software to open this file is the Similarity Evaluation System for Chromatographic Fingerprint of Traditional Chinese Medicine, and the software installation package is available in the Figure 2 folder. The OPJU file in the Figure 3 folder is the data of PCA analysis, and the open software is Origin, which can be downloaded and used for a fee from the Origin official website. [file peerj-10-13946-s002.zip › Raw date/Fig.6/MyD88/1/MyD88.jpg]

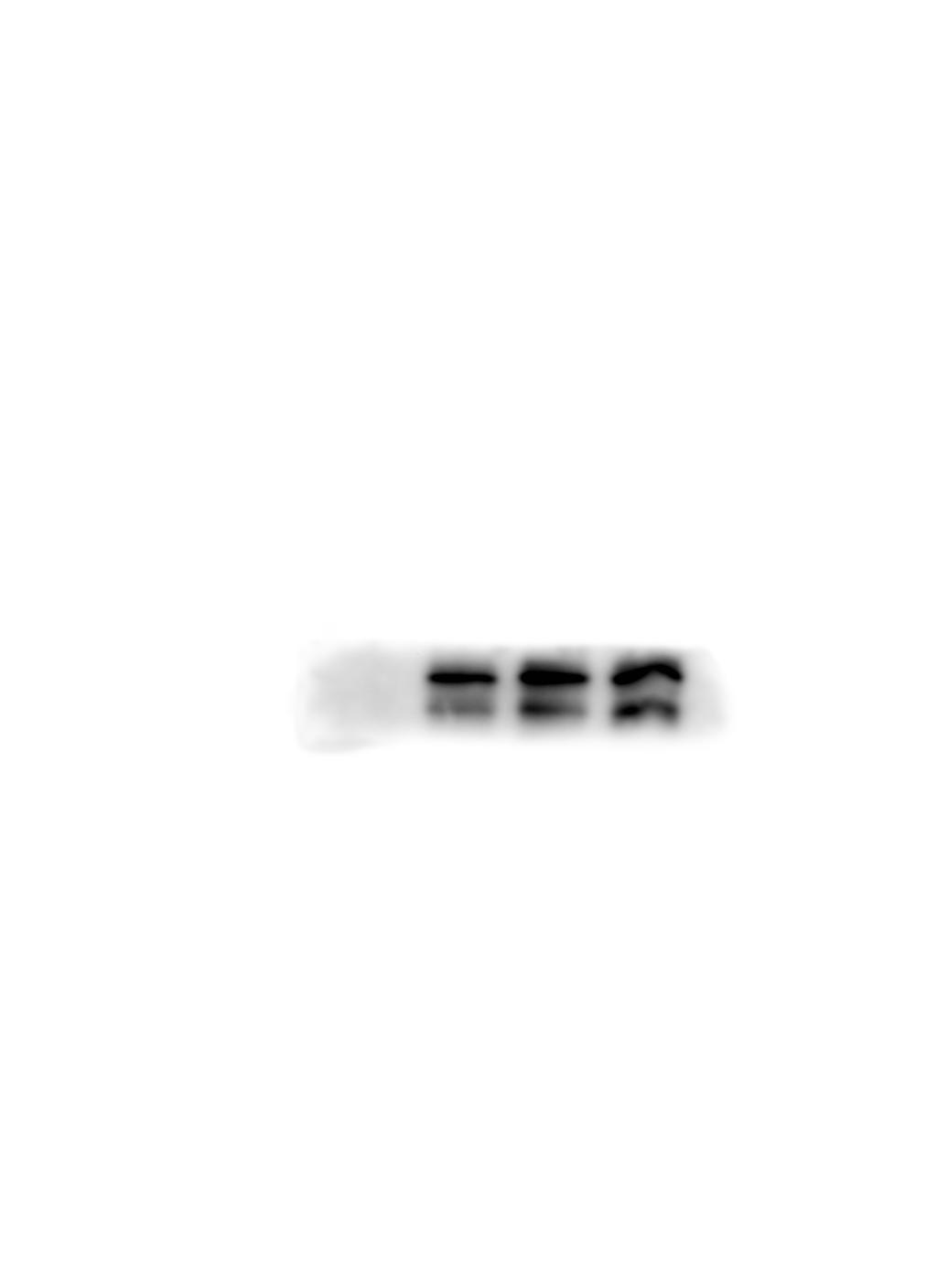

Supplement: Supplemental Information 2 — The CDF files for Fig. 2 are the data of analyzing the fingerprints of 10 batches of the rhizome of M. dauricum Polysaccharide. The software to open this file is the Similarity Evaluation System for Chromatographic Fingerprint of Traditional Chinese Medicine, and the software installation package is available in the Figure 2 folder. The OPJU file in the Figure 3 folder is the data of PCA analysis, and the open software is Origin, which can be downloaded and used for a fee from the Origin official website. [file peerj-10-13946-s002.zip › Raw date/Fig.6/MyD88/2/MyD88 .jpg]

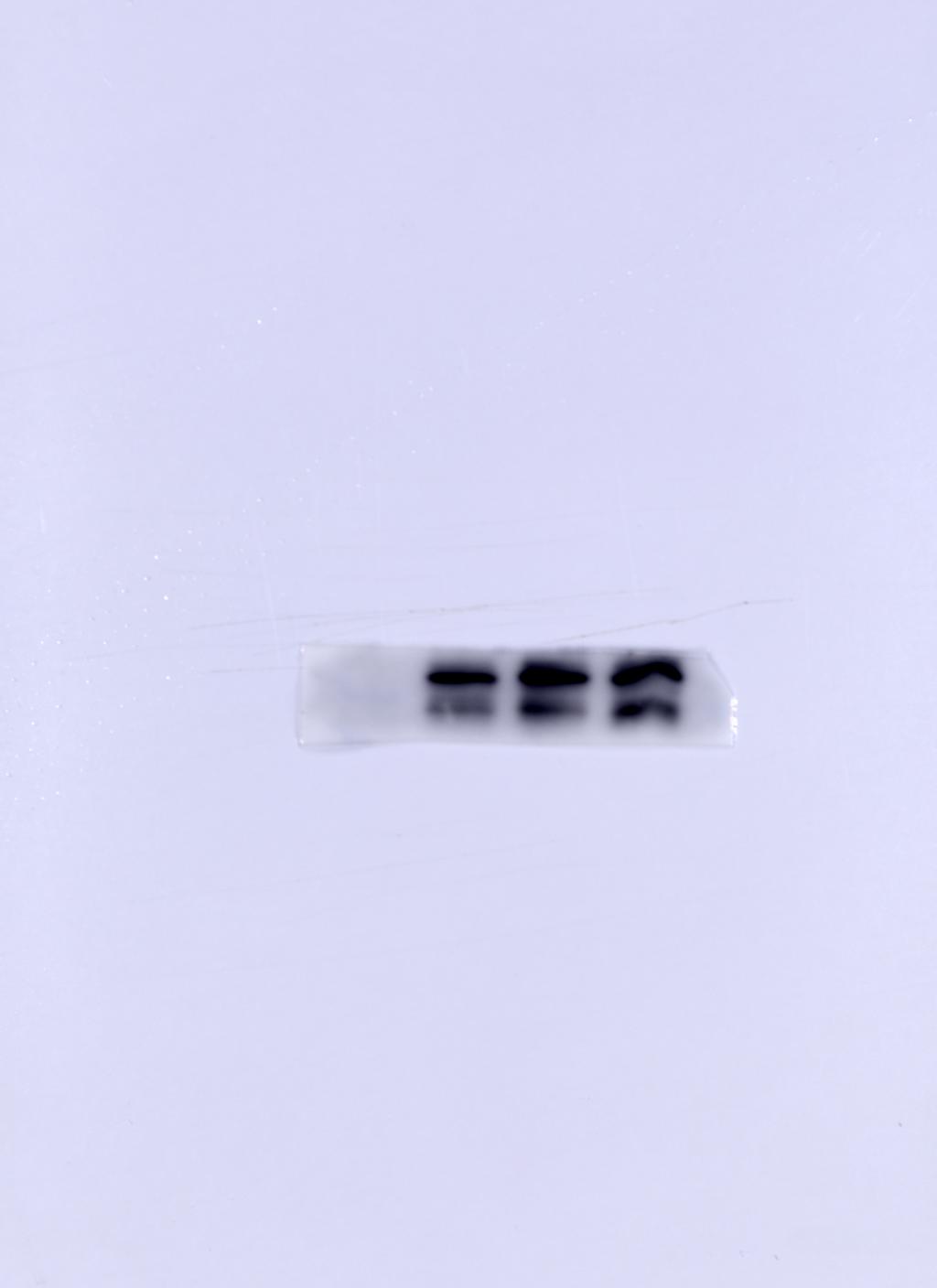

Supplement: Supplemental Information 2 — The CDF files for Fig. 2 are the data of analyzing the fingerprints of 10 batches of the rhizome of M. dauricum Polysaccharide. The software to open this file is the Similarity Evaluation System for Chromatographic Fingerprint of Traditional Chinese Medicine, and the software installation package is available in the Figure 2 folder. The OPJU file in the Figure 3 folder is the data of PCA analysis, and the open software is Origin, which can be downloaded and used for a fee from the Origin official website. [file peerj-10-13946-s002.zip › Raw date/Fig.6/MyD88/2/MyD88+Marker.jpg]

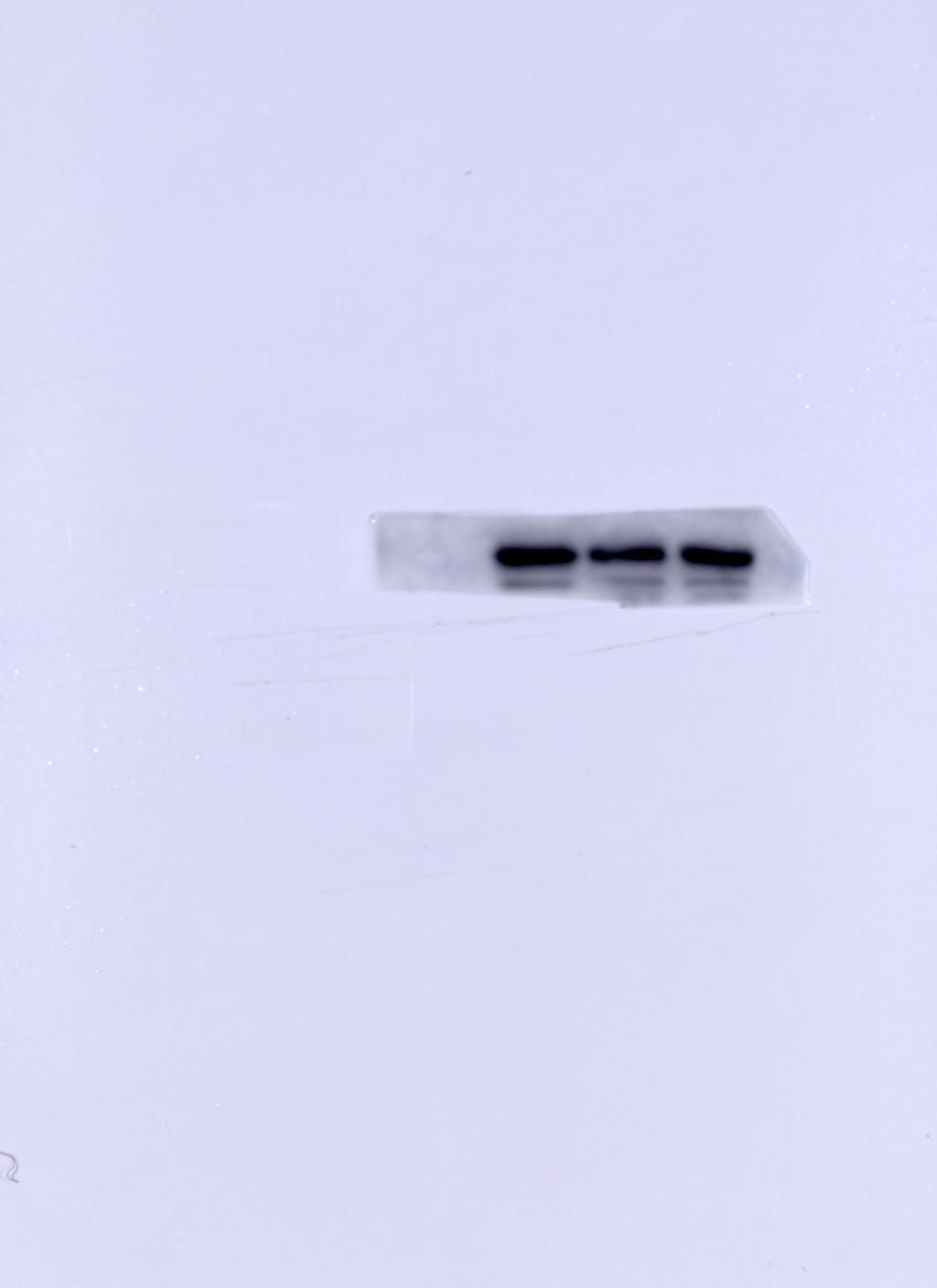

Supplement: Supplemental Information 2 — The CDF files for Fig. 2 are the data of analyzing the fingerprints of 10 batches of the rhizome of M. dauricum Polysaccharide. The software to open this file is the Similarity Evaluation System for Chromatographic Fingerprint of Traditional Chinese Medicine, and the software installation package is available in the Figure 2 folder. The OPJU file in the Figure 3 folder is the data of PCA analysis, and the open software is Origin, which can be downloaded and used for a fee from the Origin official website. [file peerj-10-13946-s002.zip › Raw date/Fig.6/MyD88/2/MyD88-GAPDH+Marker.jpg]

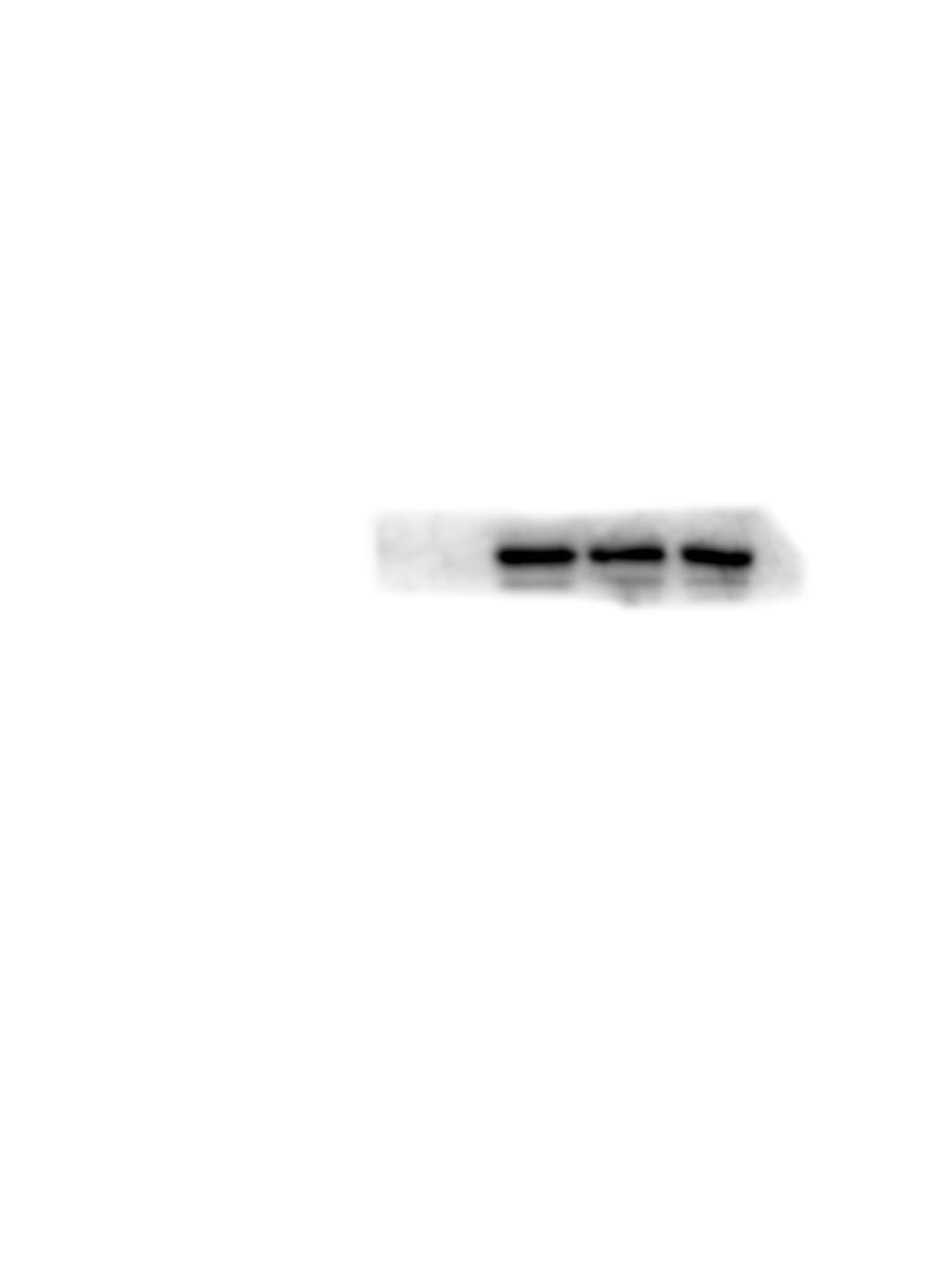

Supplement: Supplemental Information 2 — The CDF files for Fig. 2 are the data of analyzing the fingerprints of 10 batches of the rhizome of M. dauricum Polysaccharide. The software to open this file is the Similarity Evaluation System for Chromatographic Fingerprint of Traditional Chinese Medicine, and the software installation package is available in the Figure 2 folder. The OPJU file in the Figure 3 folder is the data of PCA analysis, and the open software is Origin, which can be downloaded and used for a fee from the Origin official website. [file peerj-10-13946-s002.zip › Raw date/Fig.6/MyD88/2/MyD88-GAPDH.jpg]

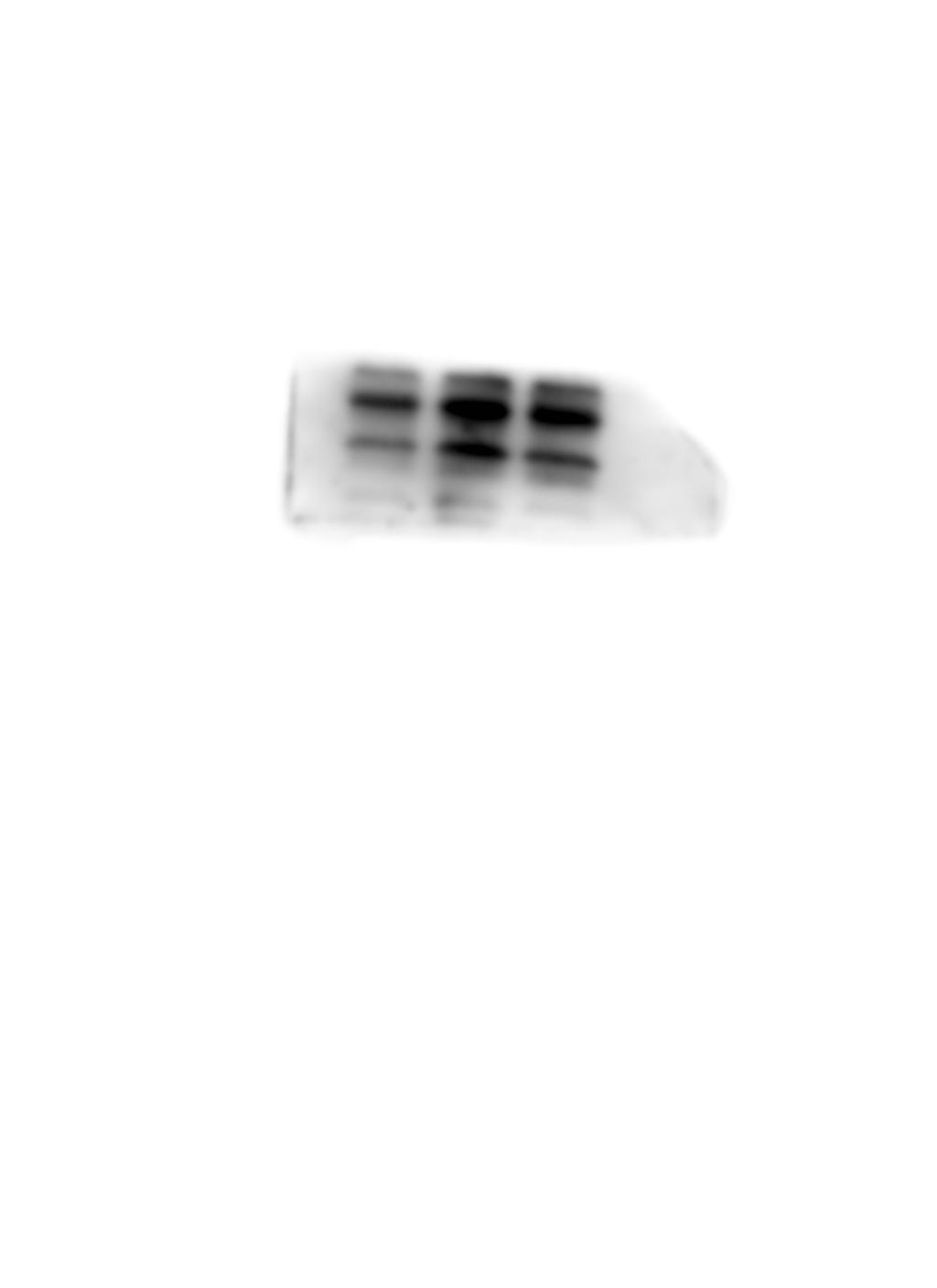

Supplement: Supplemental Information 2 — The CDF files for Fig. 2 are the data of analyzing the fingerprints of 10 batches of the rhizome of M. dauricum Polysaccharide. The software to open this file is the Similarity Evaluation System for Chromatographic Fingerprint of Traditional Chinese Medicine, and the software installation package is available in the Figure 2 folder. The OPJU file in the Figure 3 folder is the data of PCA analysis, and the open software is Origin, which can be downloaded and used for a fee from the Origin official website. [file peerj-10-13946-s002.zip › Raw date/Fig.6/MyD88/3/MyD88 .jpg]

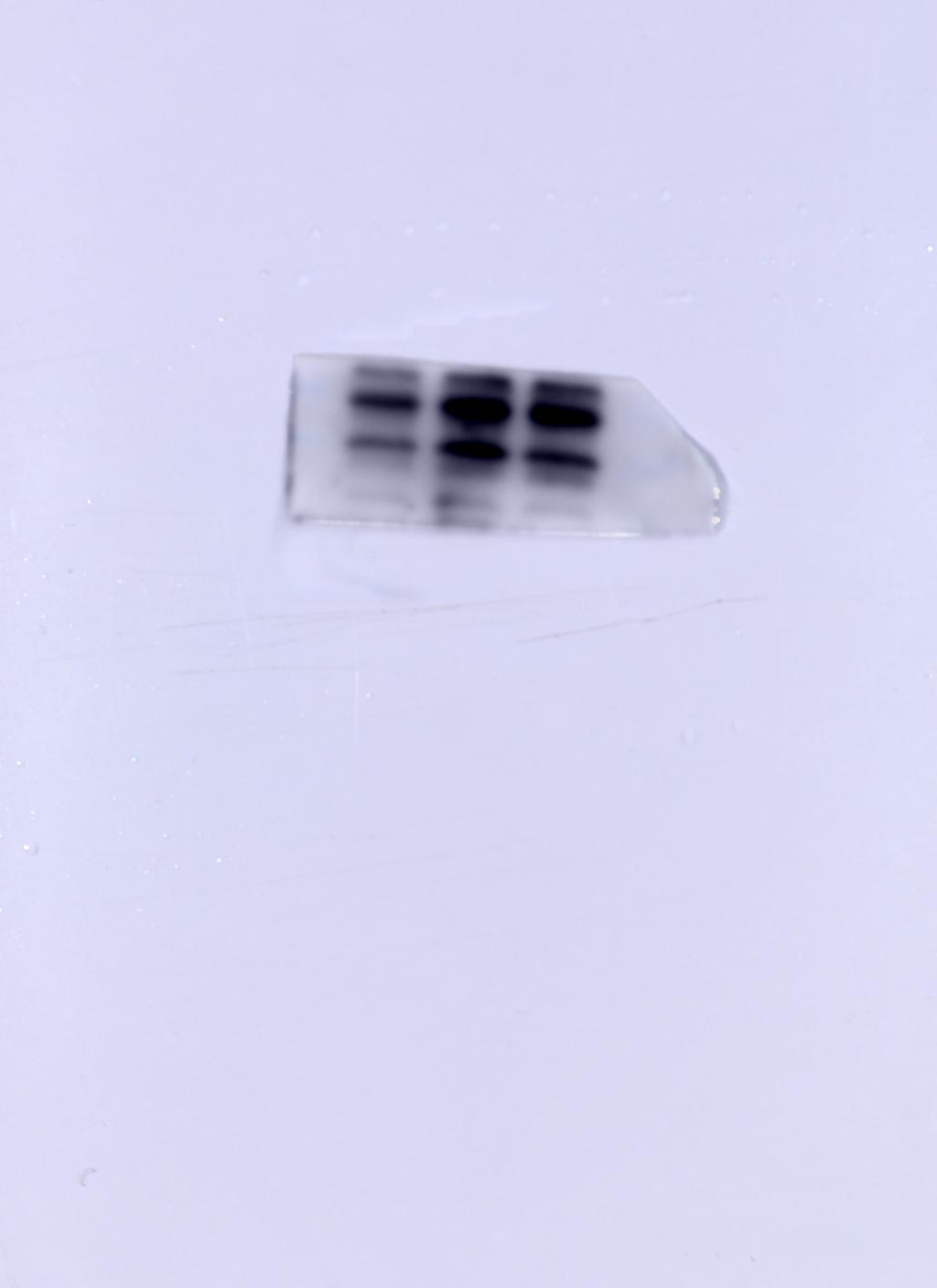

Supplement: Supplemental Information 2 — The CDF files for Fig. 2 are the data of analyzing the fingerprints of 10 batches of the rhizome of M. dauricum Polysaccharide. The software to open this file is the Similarity Evaluation System for Chromatographic Fingerprint of Traditional Chinese Medicine, and the software installation package is available in the Figure 2 folder. The OPJU file in the Figure 3 folder is the data of PCA analysis, and the open software is Origin, which can be downloaded and used for a fee from the Origin official website. [file peerj-10-13946-s002.zip › Raw date/Fig.6/MyD88/3/MyD88+Marker.jpg]

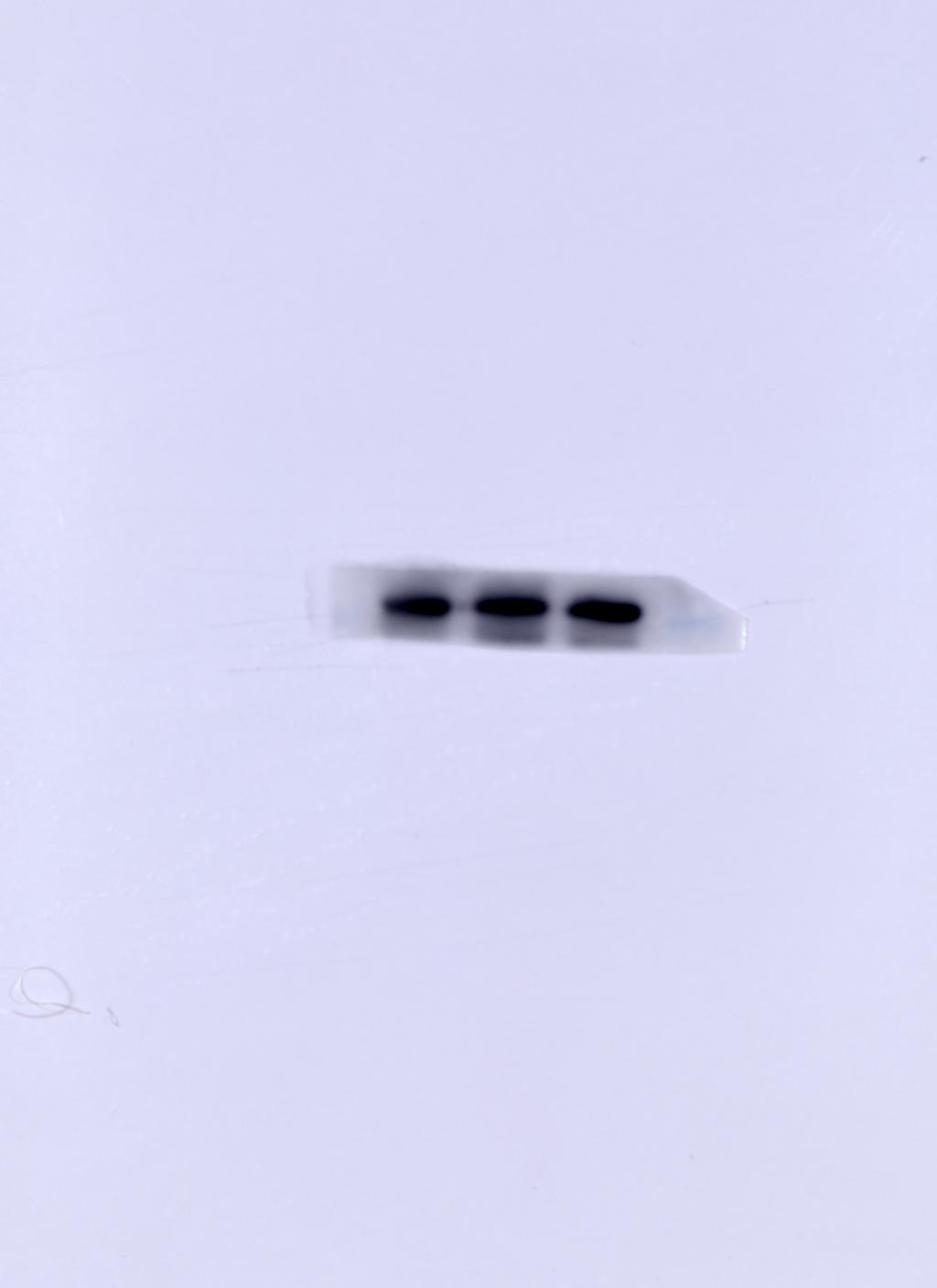

Supplement: Supplemental Information 2 — The CDF files for Fig. 2 are the data of analyzing the fingerprints of 10 batches of the rhizome of M. dauricum Polysaccharide. The software to open this file is the Similarity Evaluation System for Chromatographic Fingerprint of Traditional Chinese Medicine, and the software installation package is available in the Figure 2 folder. The OPJU file in the Figure 3 folder is the data of PCA analysis, and the open software is Origin, which can be downloaded and used for a fee from the Origin official website. [file peerj-10-13946-s002.zip › Raw date/Fig.6/MyD88/3/MyD88-GAPDH+Marker.jpg]

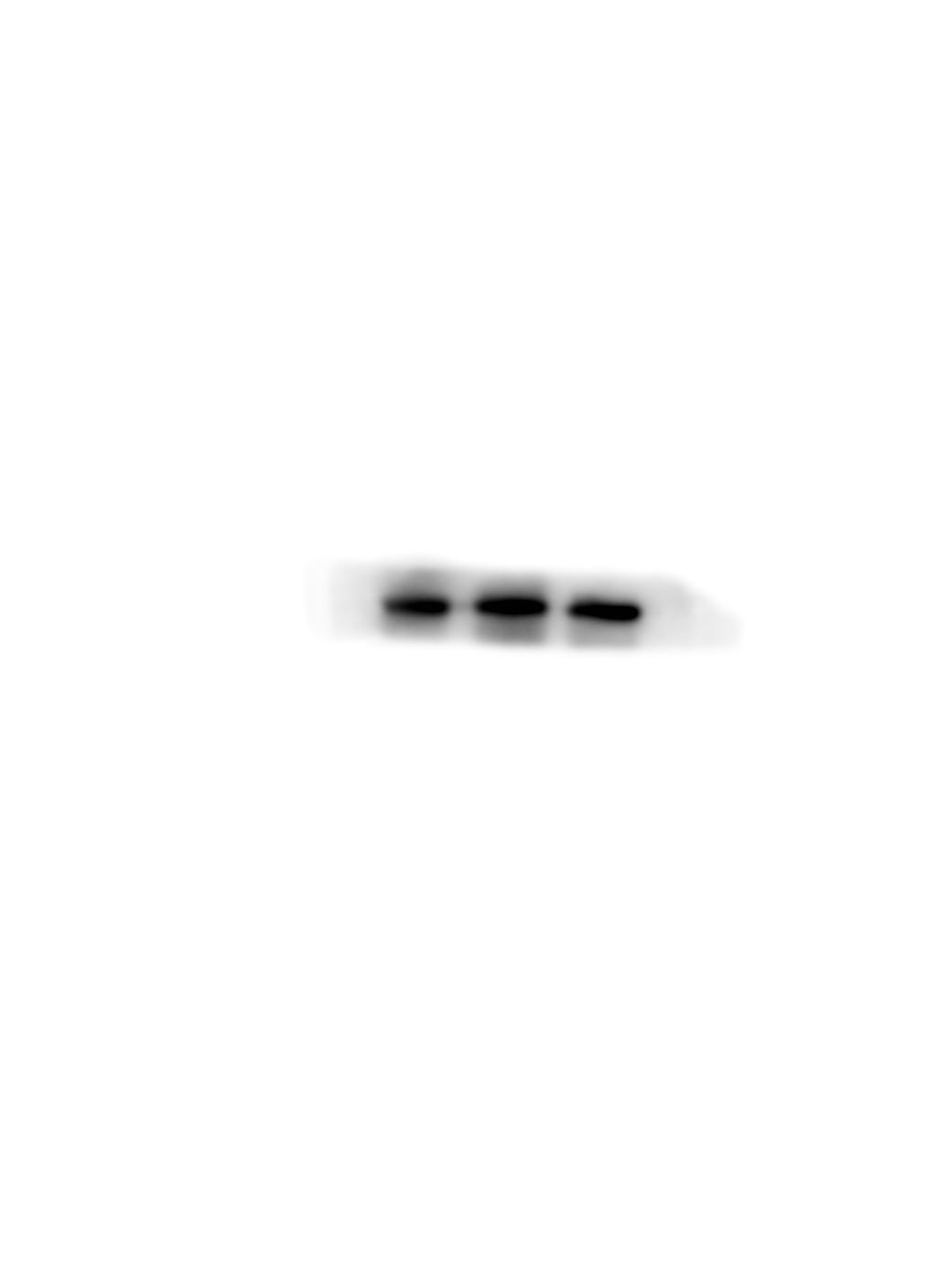

Supplement: Supplemental Information 2 — The CDF files for Fig. 2 are the data of analyzing the fingerprints of 10 batches of the rhizome of M. dauricum Polysaccharide. The software to open this file is the Similarity Evaluation System for Chromatographic Fingerprint of Traditional Chinese Medicine, and the software installation package is available in the Figure 2 folder. The OPJU file in the Figure 3 folder is the data of PCA analysis, and the open software is Origin, which can be downloaded and used for a fee from the Origin official website. [file peerj-10-13946-s002.zip › Raw date/Fig.6/MyD88/3/MyD88-GAPDH.jpg]

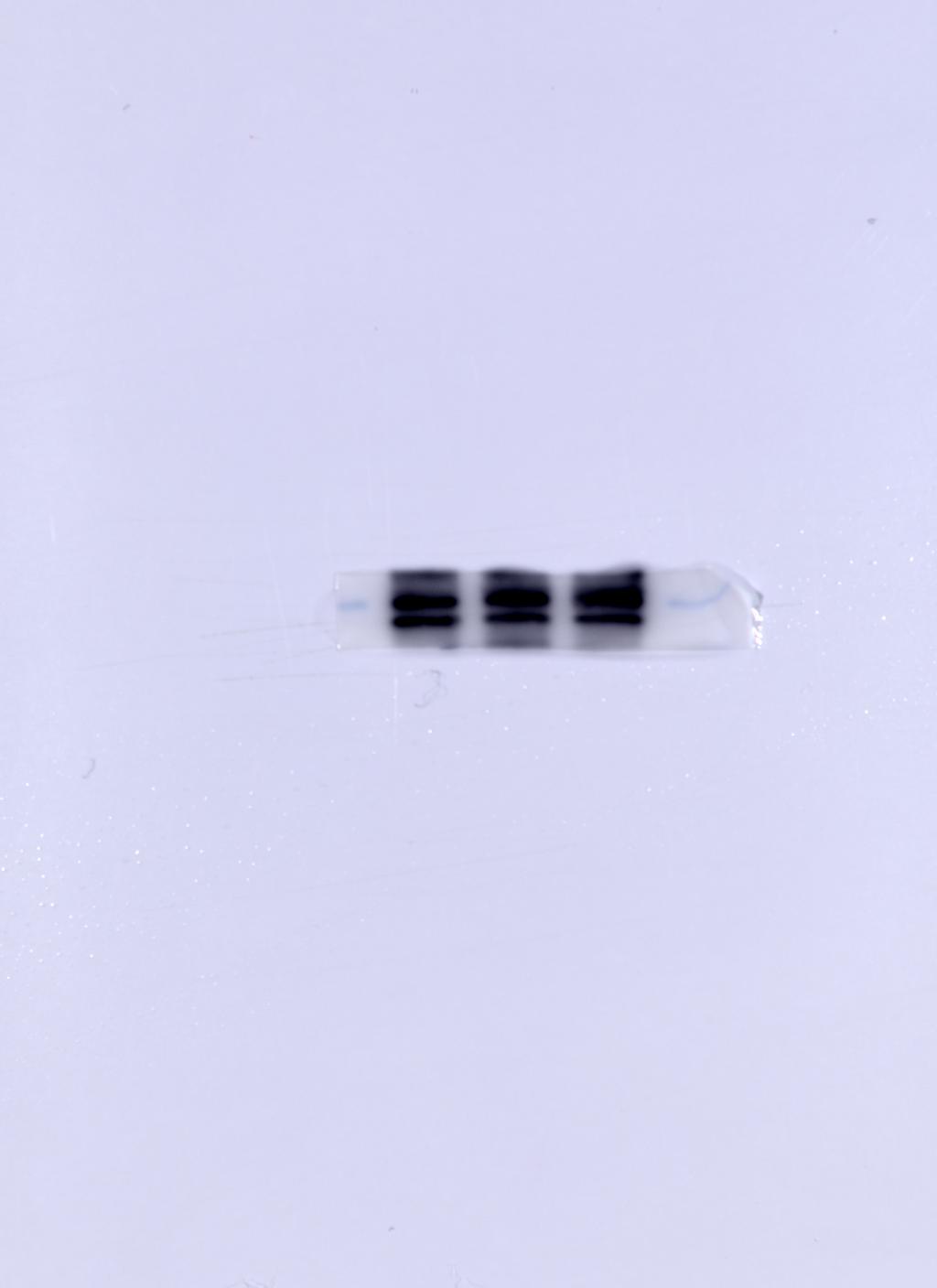

Supplement: Supplemental Information 2 — The CDF files for Fig. 2 are the data of analyzing the fingerprints of 10 batches of the rhizome of M. dauricum Polysaccharide. The software to open this file is the Similarity Evaluation System for Chromatographic Fingerprint of Traditional Chinese Medicine, and the software installation package is available in the Figure 2 folder. The OPJU file in the Figure 3 folder is the data of PCA analysis, and the open software is Origin, which can be downloaded and used for a fee from the Origin official website. [file peerj-10-13946-s002.zip › Raw date/Fig.6/p-ERK/1/ERK+Marker.jpg]

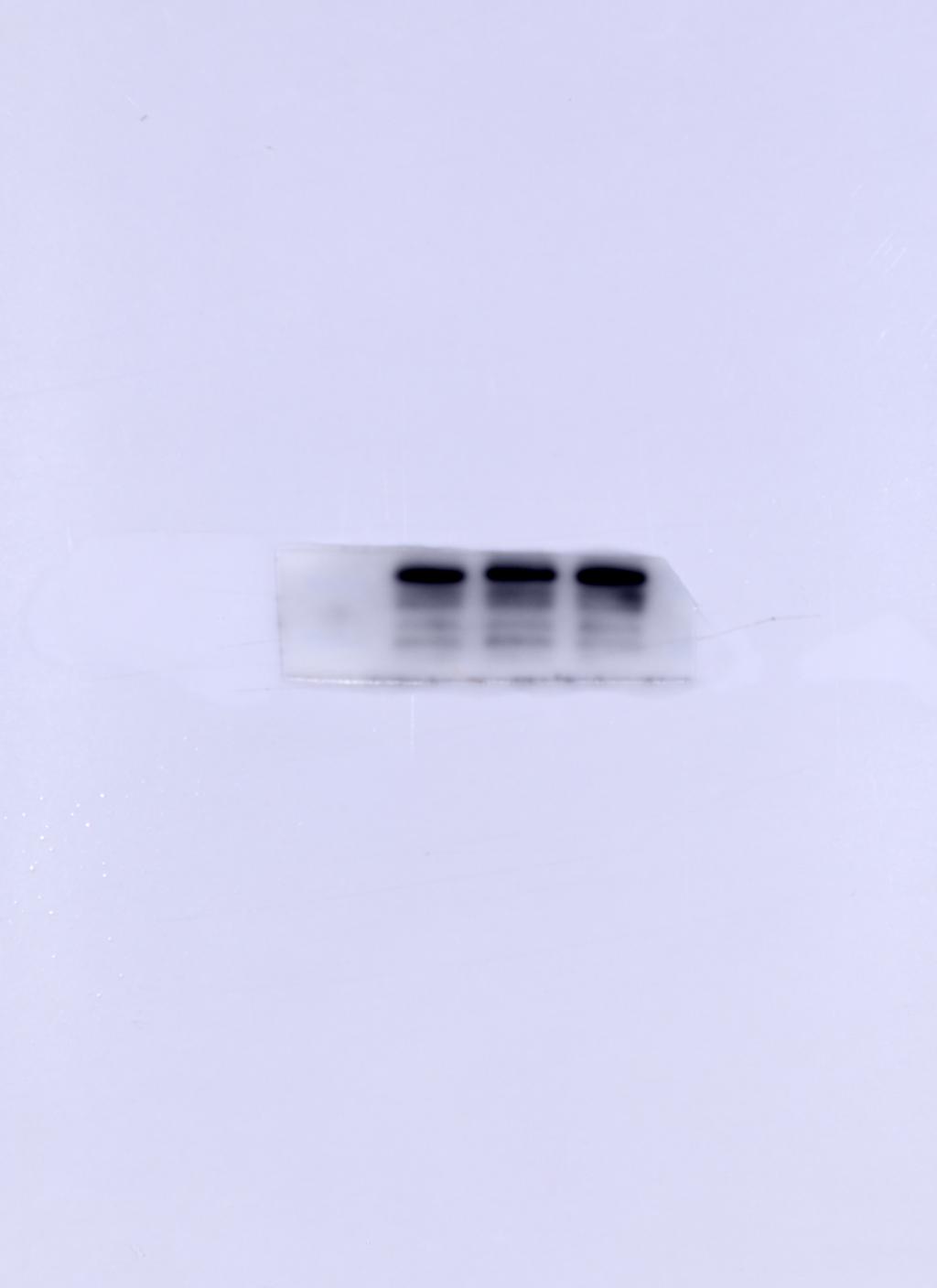

Supplement: Supplemental Information 2 — The CDF files for Fig. 2 are the data of analyzing the fingerprints of 10 batches of the rhizome of M. dauricum Polysaccharide. The software to open this file is the Similarity Evaluation System for Chromatographic Fingerprint of Traditional Chinese Medicine, and the software installation package is available in the Figure 2 folder. The OPJU file in the Figure 3 folder is the data of PCA analysis, and the open software is Origin, which can be downloaded and used for a fee from the Origin official website. [file peerj-10-13946-s002.zip › Raw date/Fig.6/p-ERK/1/ERK-GAPDH+Marker.jpg]

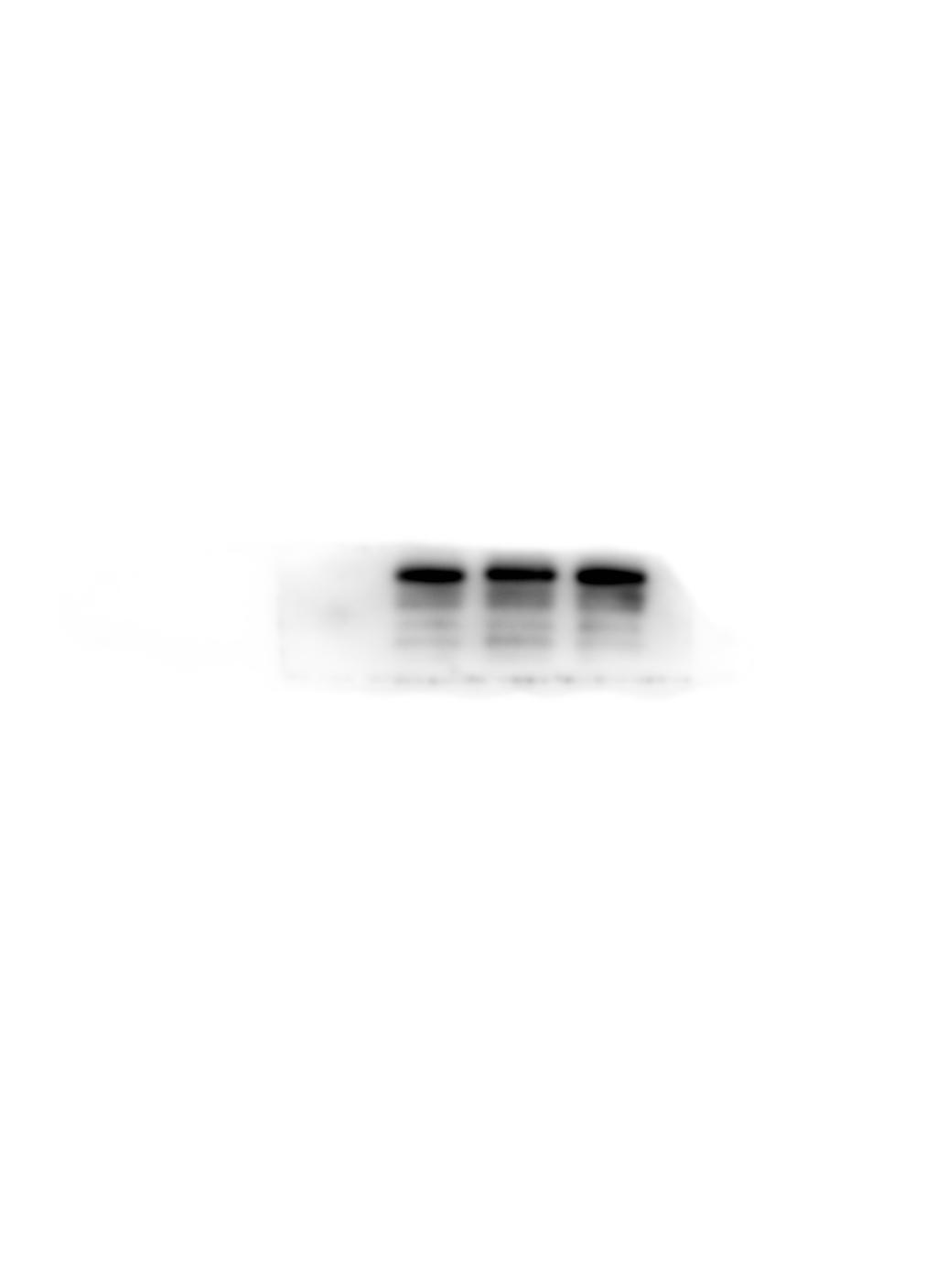

Supplement: Supplemental Information 2 — The CDF files for Fig. 2 are the data of analyzing the fingerprints of 10 batches of the rhizome of M. dauricum Polysaccharide. The software to open this file is the Similarity Evaluation System for Chromatographic Fingerprint of Traditional Chinese Medicine, and the software installation package is available in the Figure 2 folder. The OPJU file in the Figure 3 folder is the data of PCA analysis, and the open software is Origin, which can be downloaded and used for a fee from the Origin official website. [file peerj-10-13946-s002.zip › Raw date/Fig.6/p-ERK/1/ERK-GAPDH.jpg]

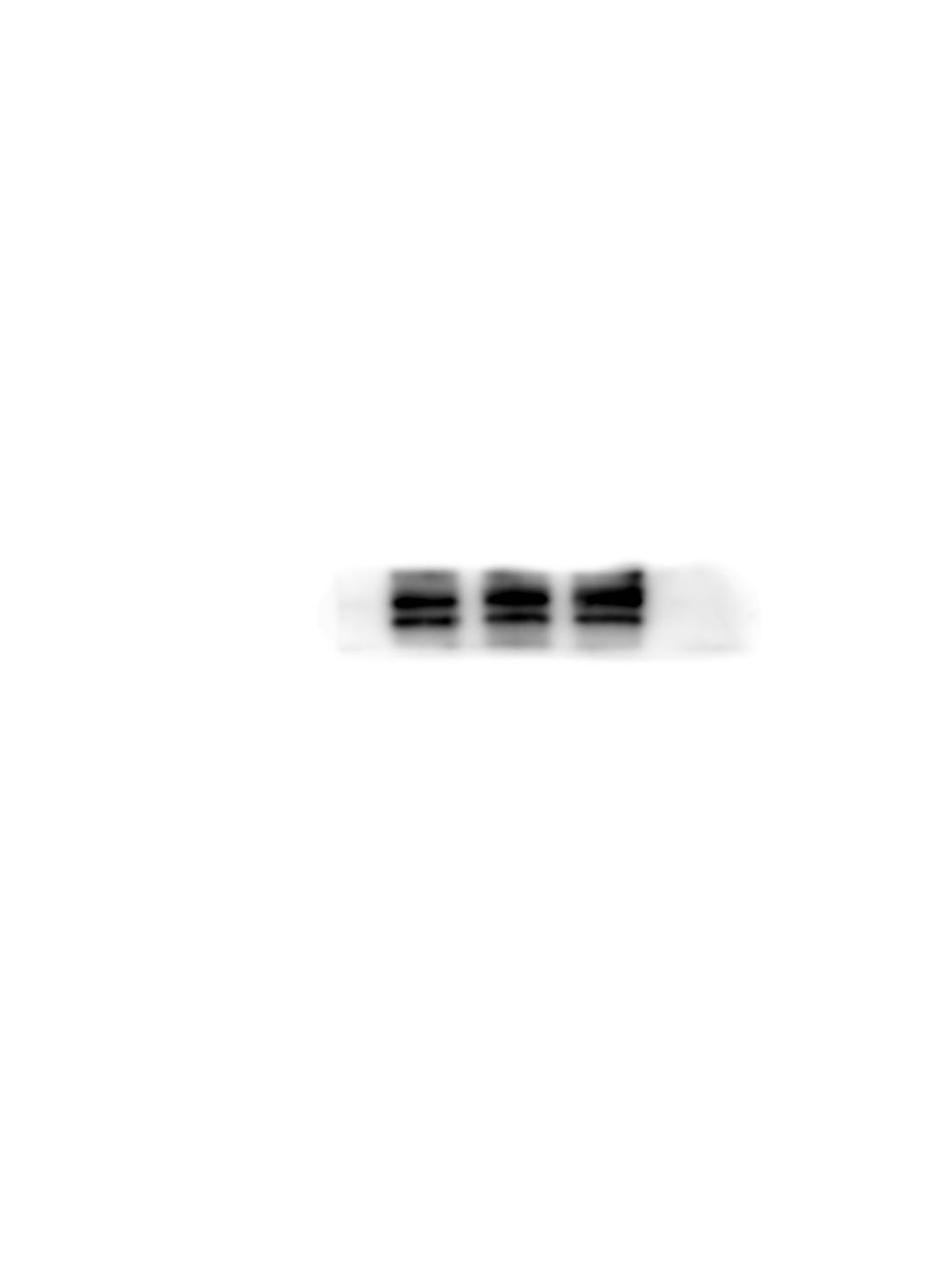

Supplement: Supplemental Information 2 — The CDF files for Fig. 2 are the data of analyzing the fingerprints of 10 batches of the rhizome of M. dauricum Polysaccharide. The software to open this file is the Similarity Evaluation System for Chromatographic Fingerprint of Traditional Chinese Medicine, and the software installation package is available in the Figure 2 folder. The OPJU file in the Figure 3 folder is the data of PCA analysis, and the open software is Origin, which can be downloaded and used for a fee from the Origin official website. [file peerj-10-13946-s002.zip › Raw date/Fig.6/p-ERK/1/ERK.jpg]

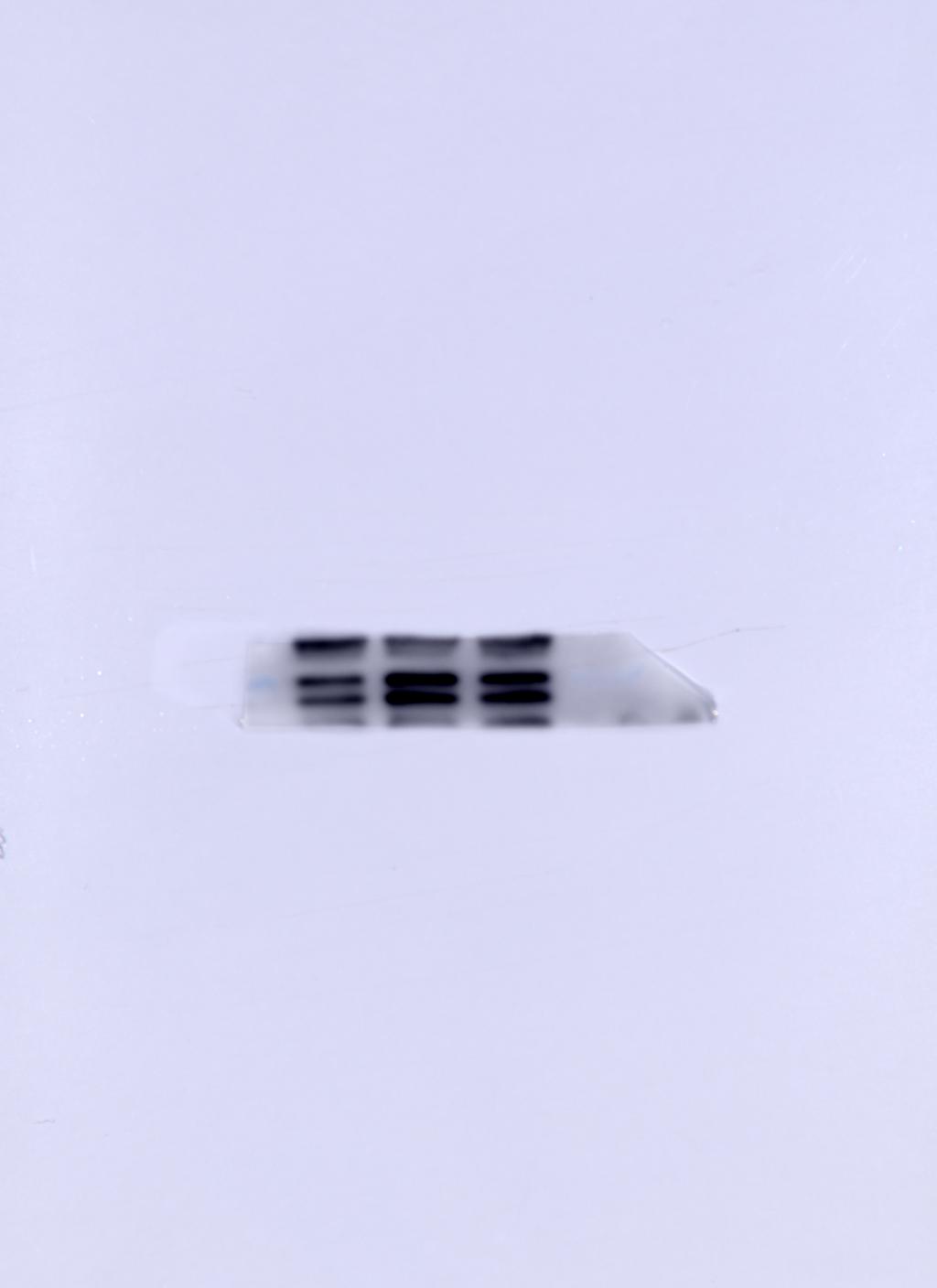

Supplement: Supplemental Information 2 — The CDF files for Fig. 2 are the data of analyzing the fingerprints of 10 batches of the rhizome of M. dauricum Polysaccharide. The software to open this file is the Similarity Evaluation System for Chromatographic Fingerprint of Traditional Chinese Medicine, and the software installation package is available in the Figure 2 folder. The OPJU file in the Figure 3 folder is the data of PCA analysis, and the open software is Origin, which can be downloaded and used for a fee from the Origin official website. [file peerj-10-13946-s002.zip › Raw date/Fig.6/p-ERK/1/p-ERK+Marker.jpg]

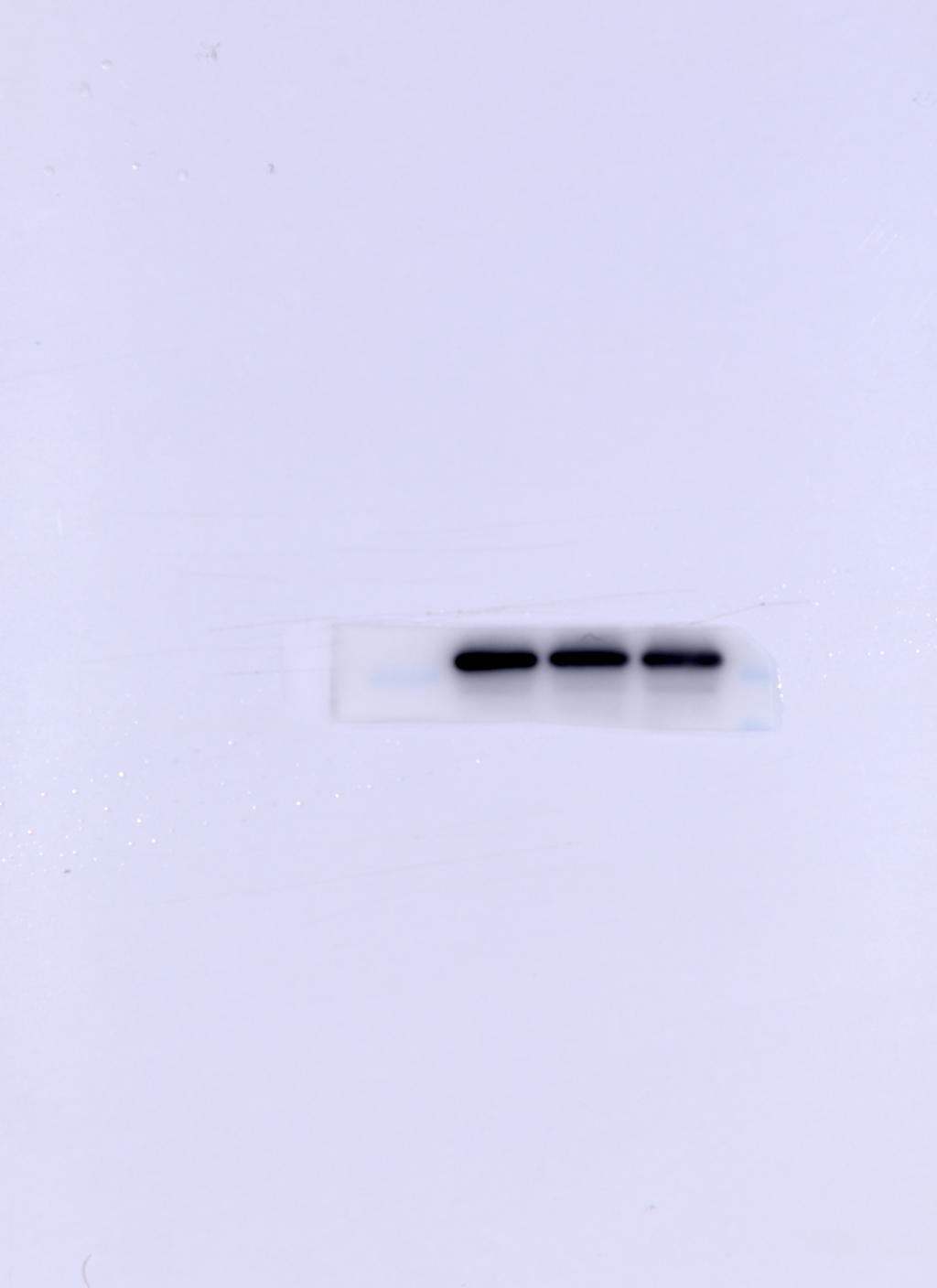

Supplement: Supplemental Information 2 — The CDF files for Fig. 2 are the data of analyzing the fingerprints of 10 batches of the rhizome of M. dauricum Polysaccharide. The software to open this file is the Similarity Evaluation System for Chromatographic Fingerprint of Traditional Chinese Medicine, and the software installation package is available in the Figure 2 folder. The OPJU file in the Figure 3 folder is the data of PCA analysis, and the open software is Origin, which can be downloaded and used for a fee from the Origin official website. [file peerj-10-13946-s002.zip › Raw date/Fig.6/p-ERK/1/p-ERK-GAPDH+Marker.jpg]

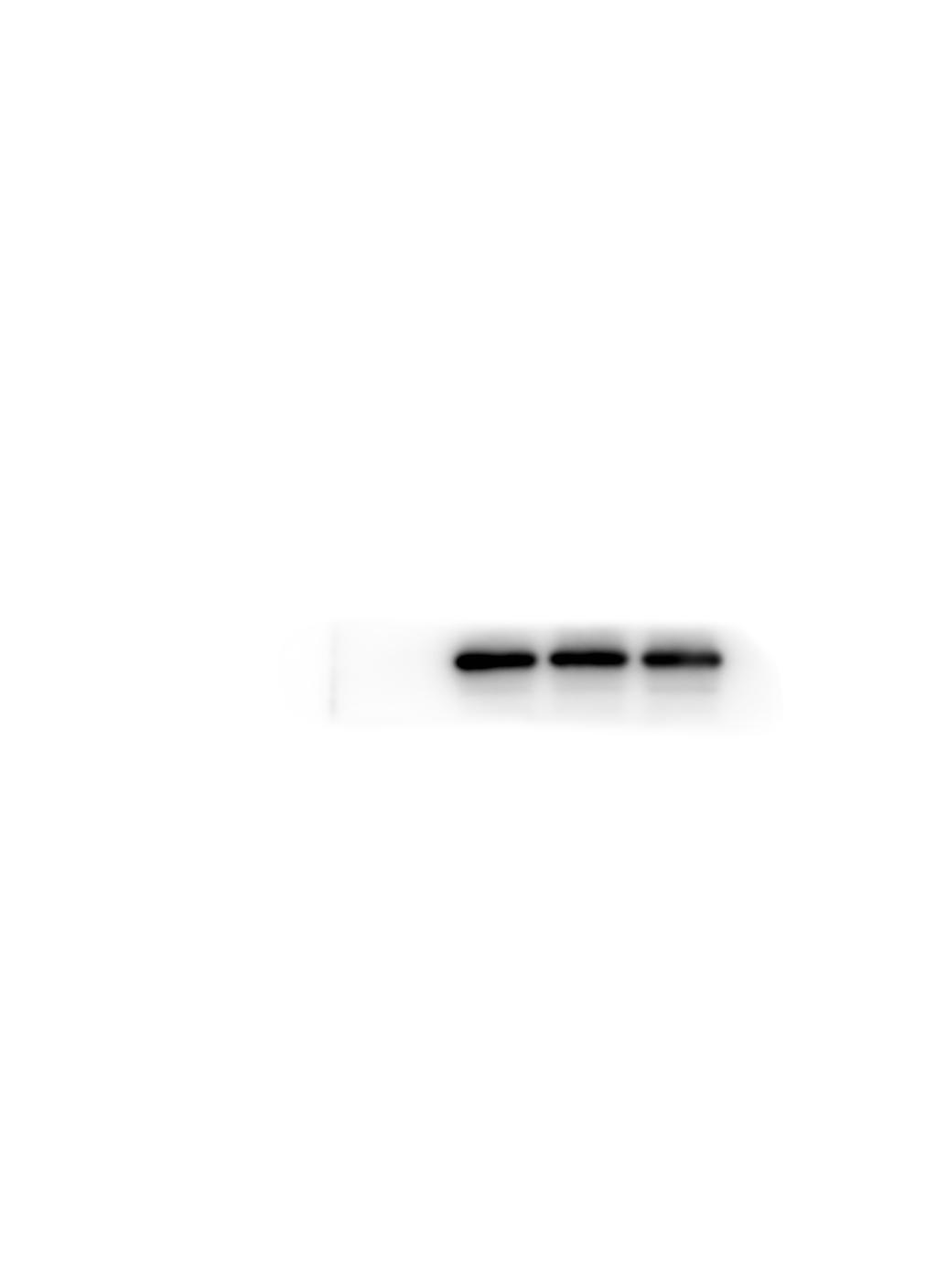

Supplement: Supplemental Information 2 — The CDF files for Fig. 2 are the data of analyzing the fingerprints of 10 batches of the rhizome of M. dauricum Polysaccharide. The software to open this file is the Similarity Evaluation System for Chromatographic Fingerprint of Traditional Chinese Medicine, and the software installation package is available in the Figure 2 folder. The OPJU file in the Figure 3 folder is the data of PCA analysis, and the open software is Origin, which can be downloaded and used for a fee from the Origin official website. [file peerj-10-13946-s002.zip › Raw date/Fig.6/p-ERK/1/p-ERK-GAPDH.jpg]

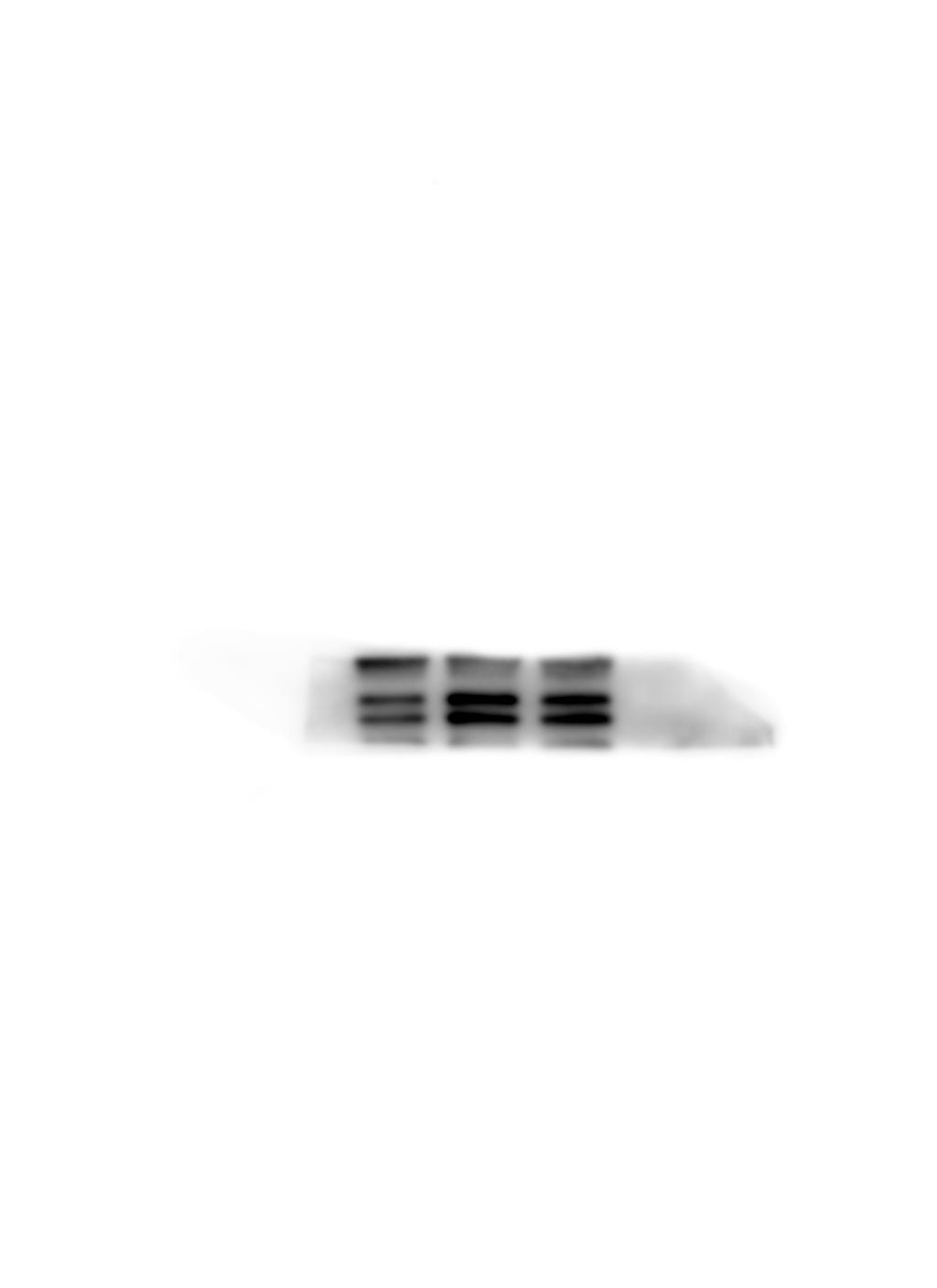

Supplement: Supplemental Information 2 — The CDF files for Fig. 2 are the data of analyzing the fingerprints of 10 batches of the rhizome of M. dauricum Polysaccharide. The software to open this file is the Similarity Evaluation System for Chromatographic Fingerprint of Traditional Chinese Medicine, and the software installation package is available in the Figure 2 folder. The OPJU file in the Figure 3 folder is the data of PCA analysis, and the open software is Origin, which can be downloaded and used for a fee from the Origin official website. [file peerj-10-13946-s002.zip › Raw date/Fig.6/p-ERK/1/p-ERK.jpg]

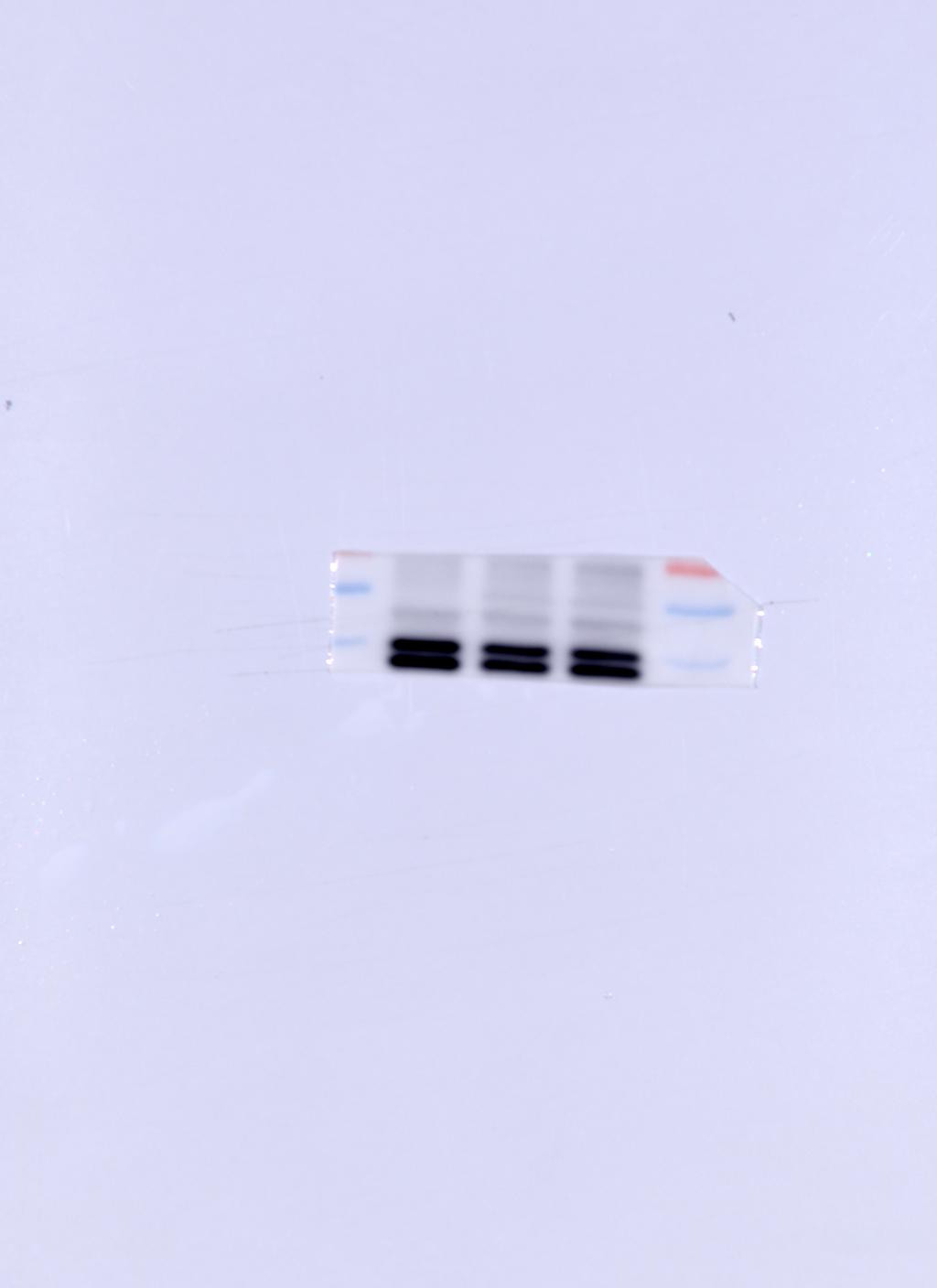

Supplement: Supplemental Information 2 — The CDF files for Fig. 2 are the data of analyzing the fingerprints of 10 batches of the rhizome of M. dauricum Polysaccharide. The software to open this file is the Similarity Evaluation System for Chromatographic Fingerprint of Traditional Chinese Medicine, and the software installation package is available in the Figure 2 folder. The OPJU file in the Figure 3 folder is the data of PCA analysis, and the open software is Origin, which can be downloaded and used for a fee from the Origin official website. [file peerj-10-13946-s002.zip › Raw date/Fig.6/p-ERK/2/ERK+Marker.jpg]

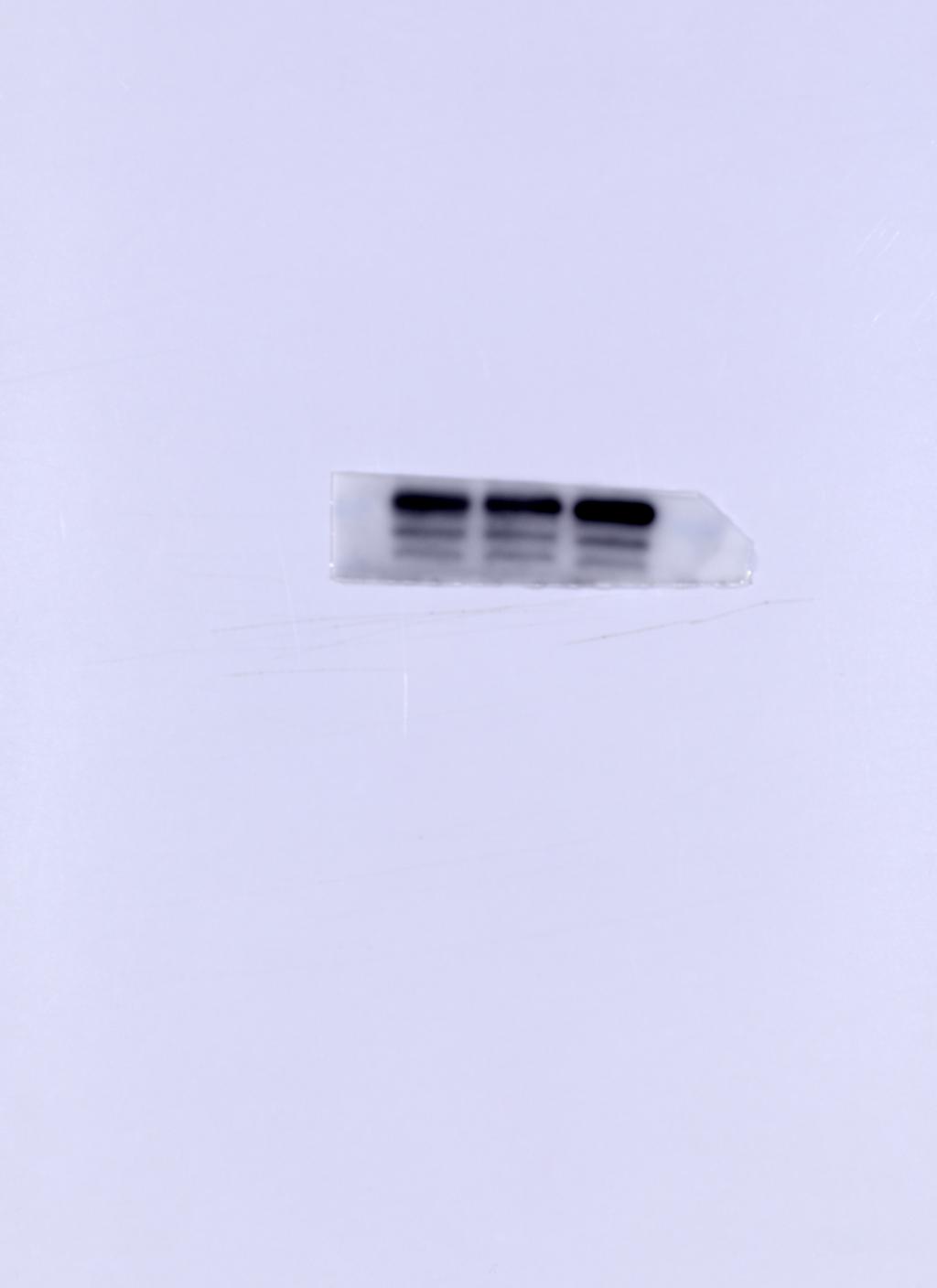

Supplement: Supplemental Information 2 — The CDF files for Fig. 2 are the data of analyzing the fingerprints of 10 batches of the rhizome of M. dauricum Polysaccharide. The software to open this file is the Similarity Evaluation System for Chromatographic Fingerprint of Traditional Chinese Medicine, and the software installation package is available in the Figure 2 folder. The OPJU file in the Figure 3 folder is the data of PCA analysis, and the open software is Origin, which can be downloaded and used for a fee from the Origin official website. [file peerj-10-13946-s002.zip › Raw date/Fig.6/p-ERK/2/ERK-GAPD+Marker.jpg]

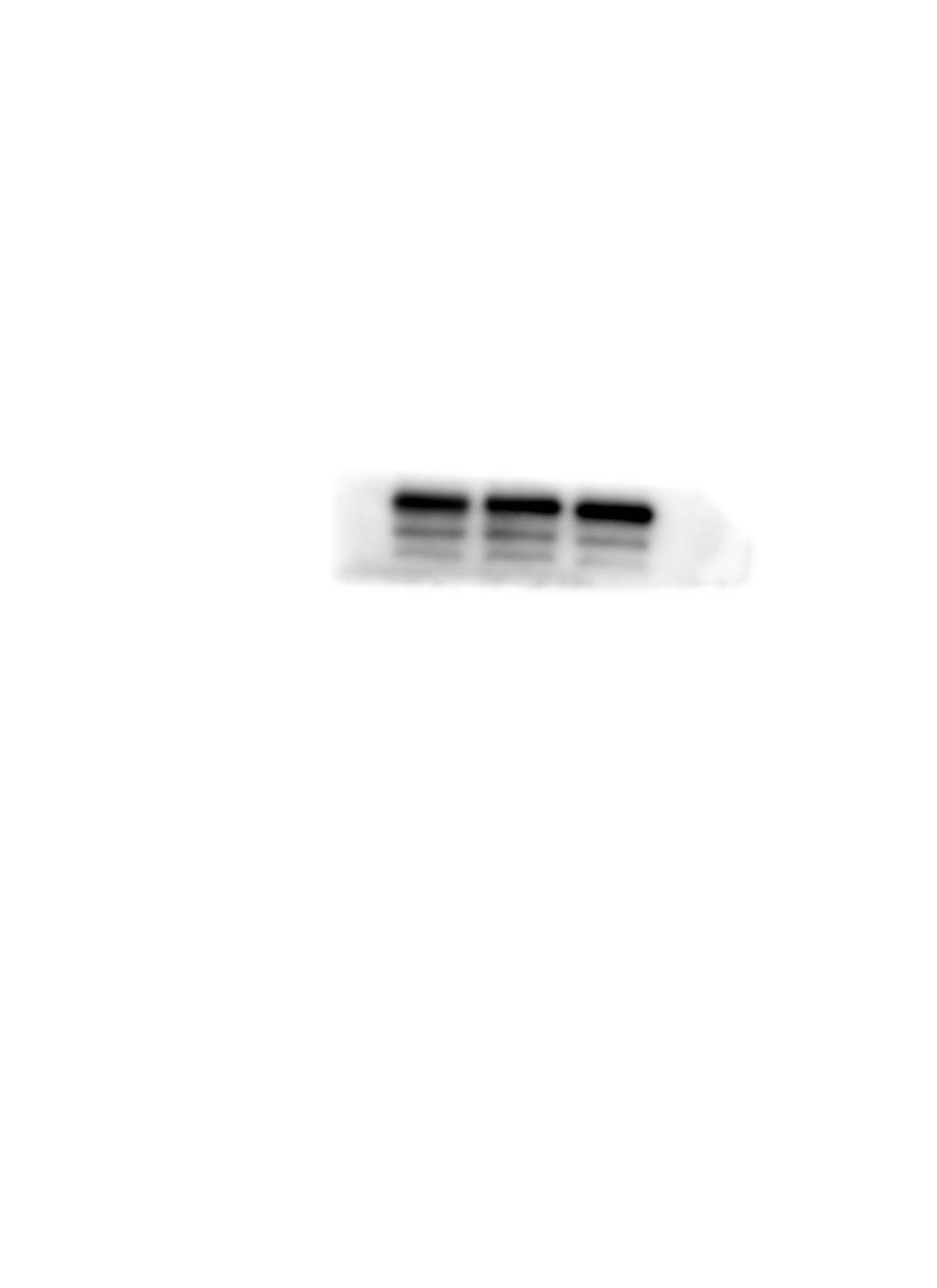

Supplement: Supplemental Information 2 — The CDF files for Fig. 2 are the data of analyzing the fingerprints of 10 batches of the rhizome of M. dauricum Polysaccharide. The software to open this file is the Similarity Evaluation System for Chromatographic Fingerprint of Traditional Chinese Medicine, and the software installation package is available in the Figure 2 folder. The OPJU file in the Figure 3 folder is the data of PCA analysis, and the open software is Origin, which can be downloaded and used for a fee from the Origin official website. [file peerj-10-13946-s002.zip › Raw date/Fig.6/p-ERK/2/ERK-GAPDH.jpg]

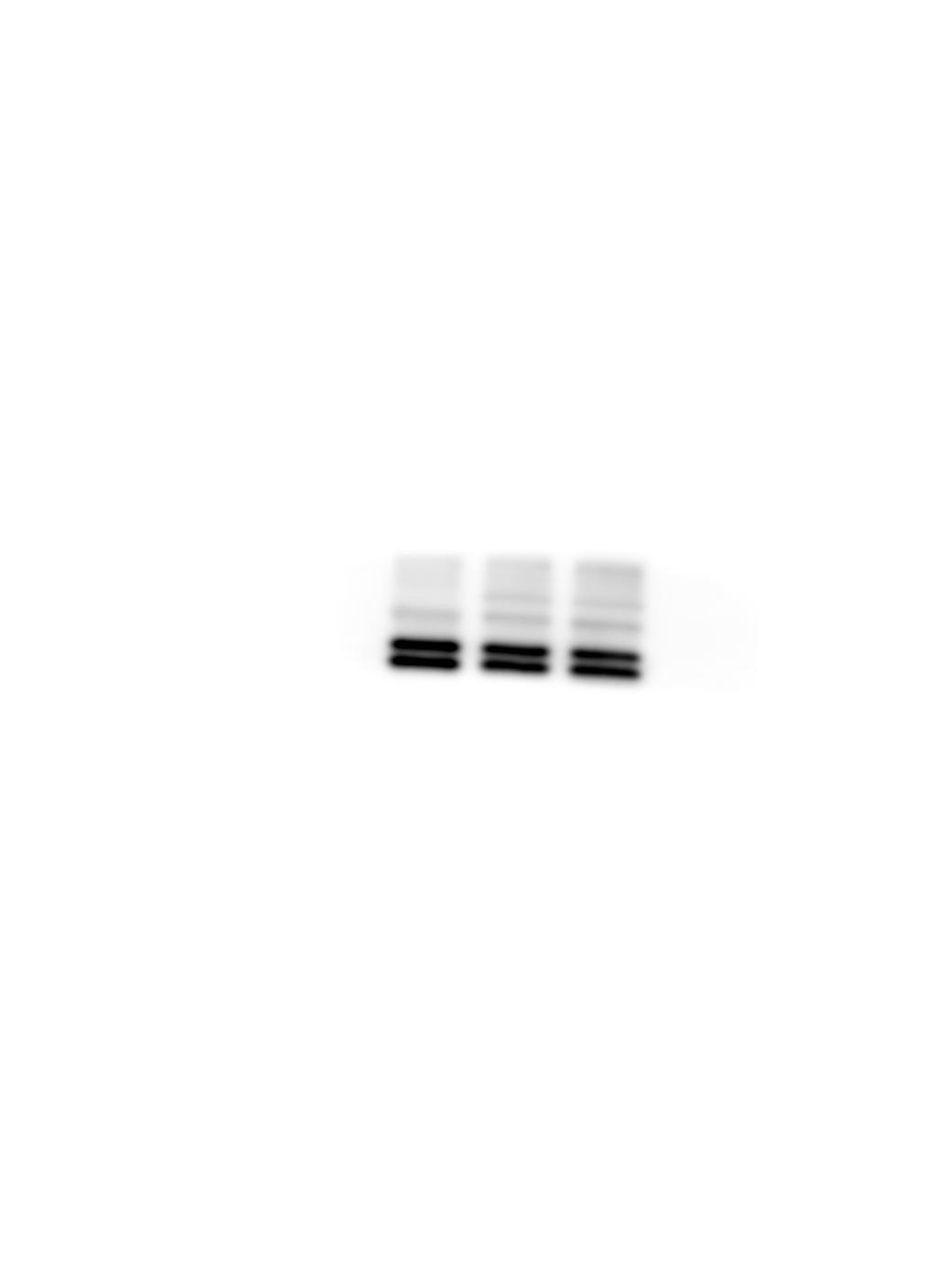

Supplement: Supplemental Information 2 — The CDF files for Fig. 2 are the data of analyzing the fingerprints of 10 batches of the rhizome of M. dauricum Polysaccharide. The software to open this file is the Similarity Evaluation System for Chromatographic Fingerprint of Traditional Chinese Medicine, and the software installation package is available in the Figure 2 folder. The OPJU file in the Figure 3 folder is the data of PCA analysis, and the open software is Origin, which can be downloaded and used for a fee from the Origin official website. [file peerj-10-13946-s002.zip › Raw date/Fig.6/p-ERK/2/ERK.jpg]

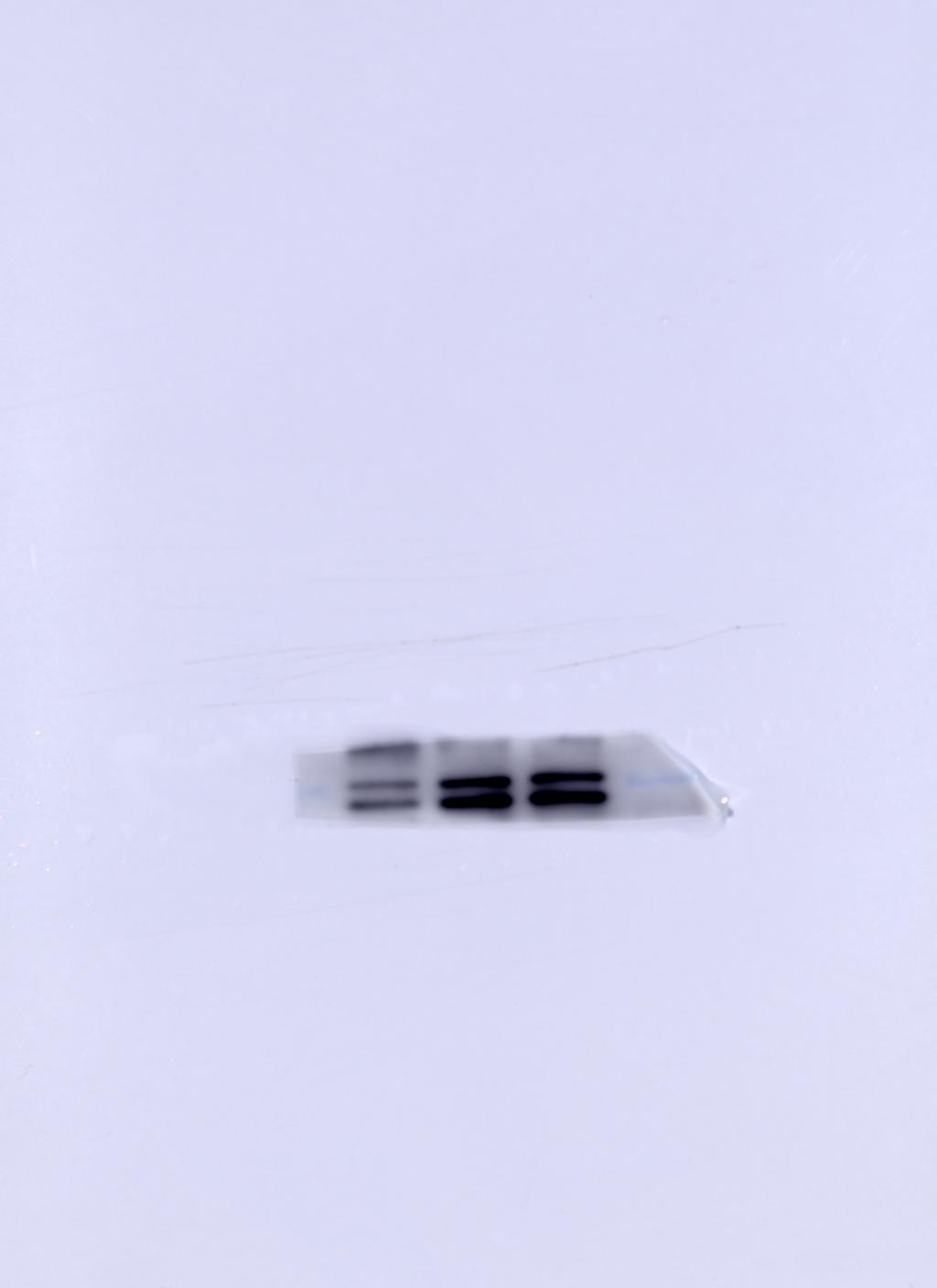

Supplement: Supplemental Information 2 — The CDF files for Fig. 2 are the data of analyzing the fingerprints of 10 batches of the rhizome of M. dauricum Polysaccharide. The software to open this file is the Similarity Evaluation System for Chromatographic Fingerprint of Traditional Chinese Medicine, and the software installation package is available in the Figure 2 folder. The OPJU file in the Figure 3 folder is the data of PCA analysis, and the open software is Origin, which can be downloaded and used for a fee from the Origin official website. [file peerj-10-13946-s002.zip › Raw date/Fig.6/p-ERK/2/p-ERK+Marker.jpg]

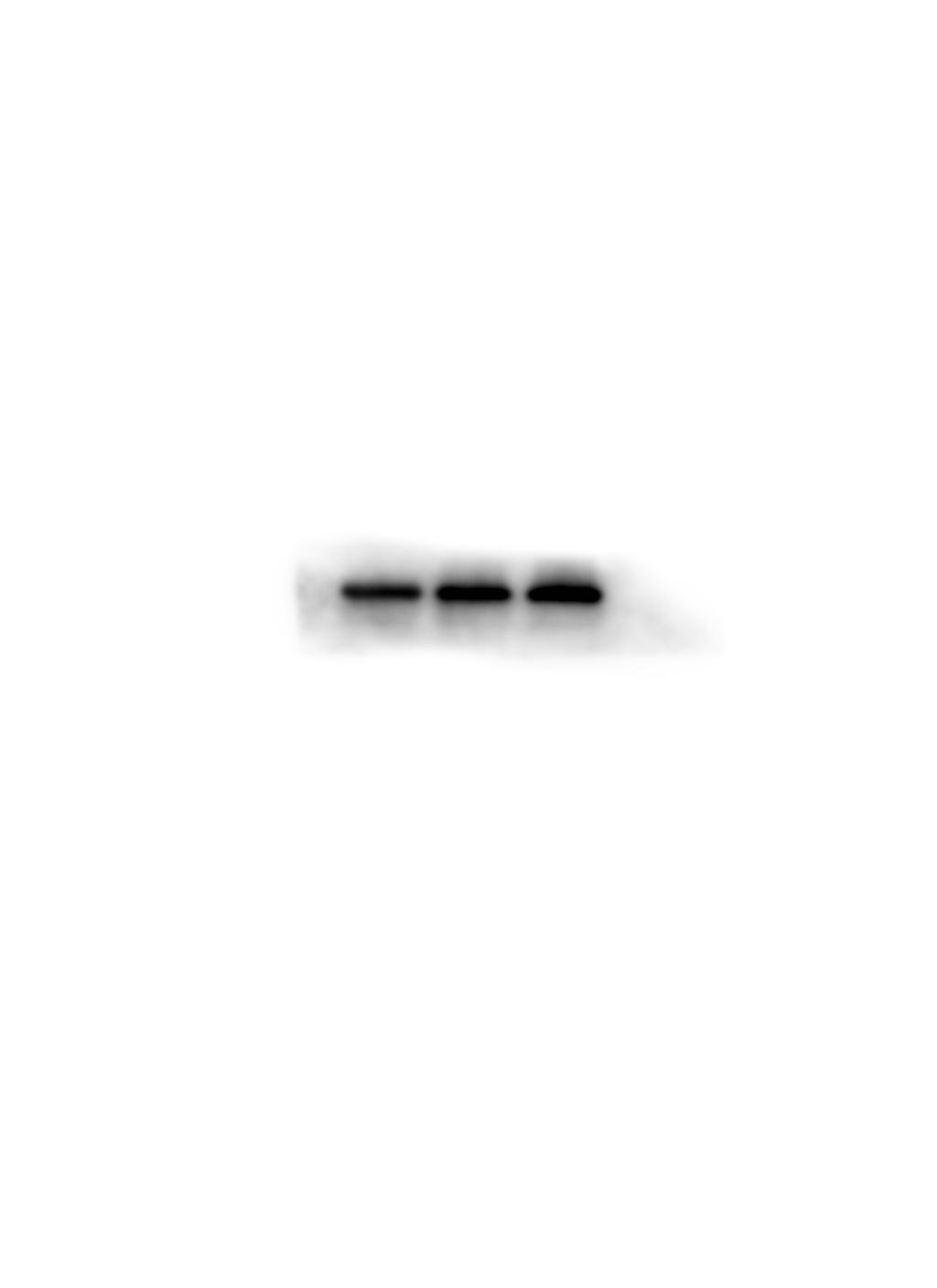

Supplement: Supplemental Information 2 — The CDF files for Fig. 2 are the data of analyzing the fingerprints of 10 batches of the rhizome of M. dauricum Polysaccharide. The software to open this file is the Similarity Evaluation System for Chromatographic Fingerprint of Traditional Chinese Medicine, and the software installation package is available in the Figure 2 folder. The OPJU file in the Figure 3 folder is the data of PCA analysis, and the open software is Origin, which can be downloaded and used for a fee from the Origin official website. [file peerj-10-13946-s002.zip › Raw date/Fig.6/p-ERK/2/p-ERK-GAPDH.jpg]

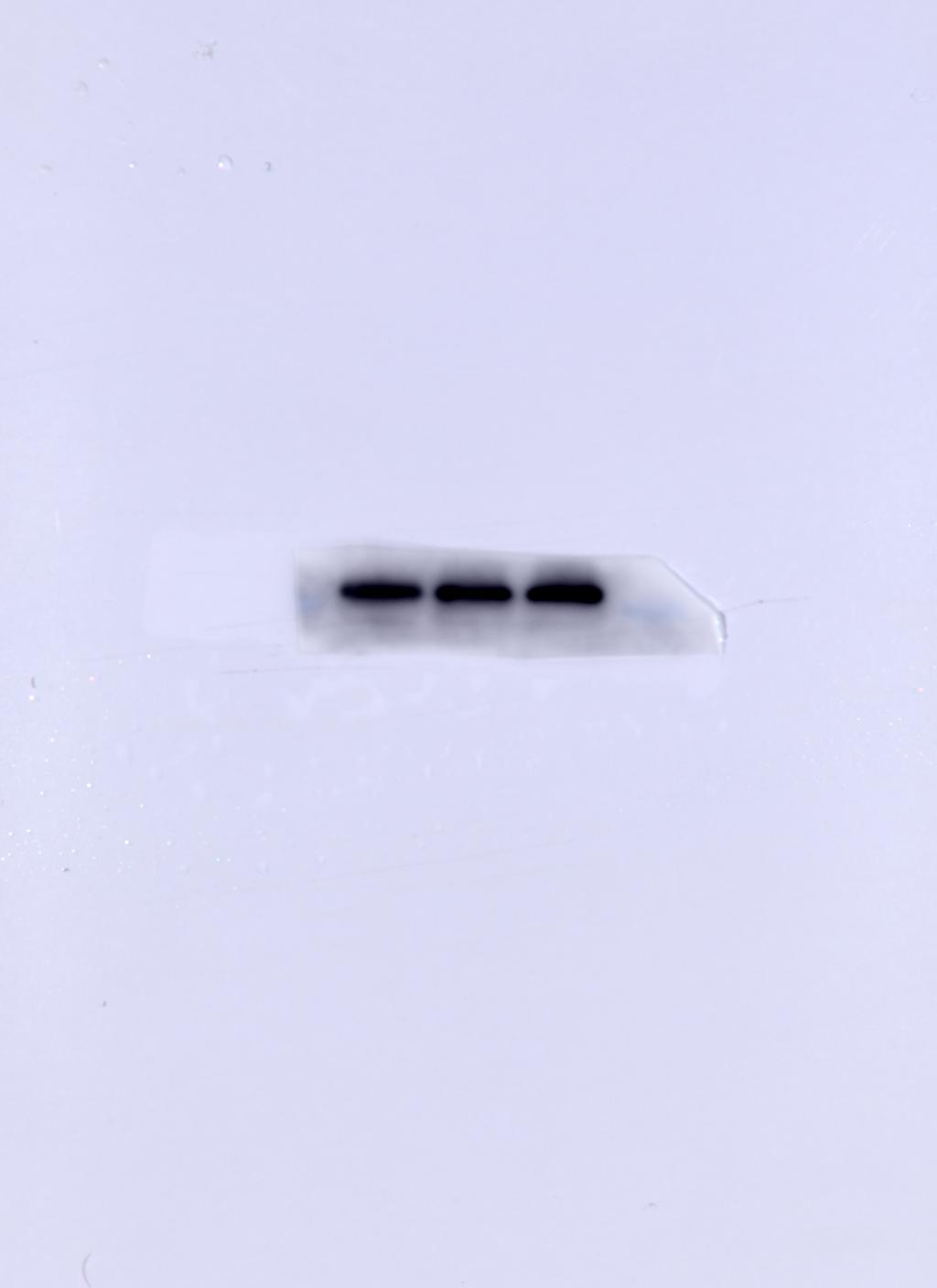

Supplement: Supplemental Information 2 — The CDF files for Fig. 2 are the data of analyzing the fingerprints of 10 batches of the rhizome of M. dauricum Polysaccharide. The software to open this file is the Similarity Evaluation System for Chromatographic Fingerprint of Traditional Chinese Medicine, and the software installation package is available in the Figure 2 folder. The OPJU file in the Figure 3 folder is the data of PCA analysis, and the open software is Origin, which can be downloaded and used for a fee from the Origin official website. [file peerj-10-13946-s002.zip › Raw date/Fig.6/p-ERK/2/p-ERK-GAPDH5+Marker.jpg]

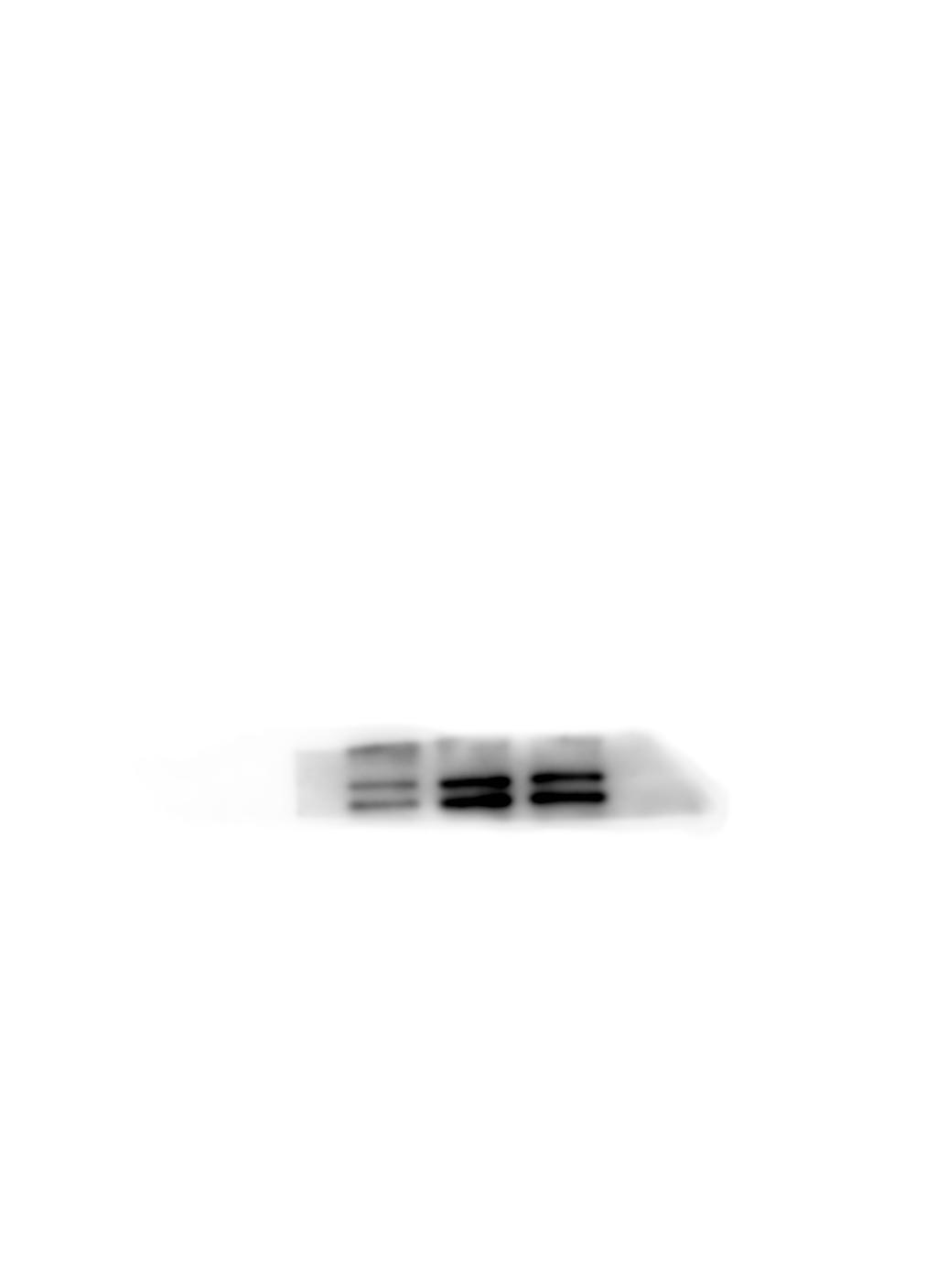

Supplement: Supplemental Information 2 — The CDF files for Fig. 2 are the data of analyzing the fingerprints of 10 batches of the rhizome of M. dauricum Polysaccharide. The software to open this file is the Similarity Evaluation System for Chromatographic Fingerprint of Traditional Chinese Medicine, and the software installation package is available in the Figure 2 folder. The OPJU file in the Figure 3 folder is the data of PCA analysis, and the open software is Origin, which can be downloaded and used for a fee from the Origin official website. [file peerj-10-13946-s002.zip › Raw date/Fig.6/p-ERK/2/p-ERK.jpg]

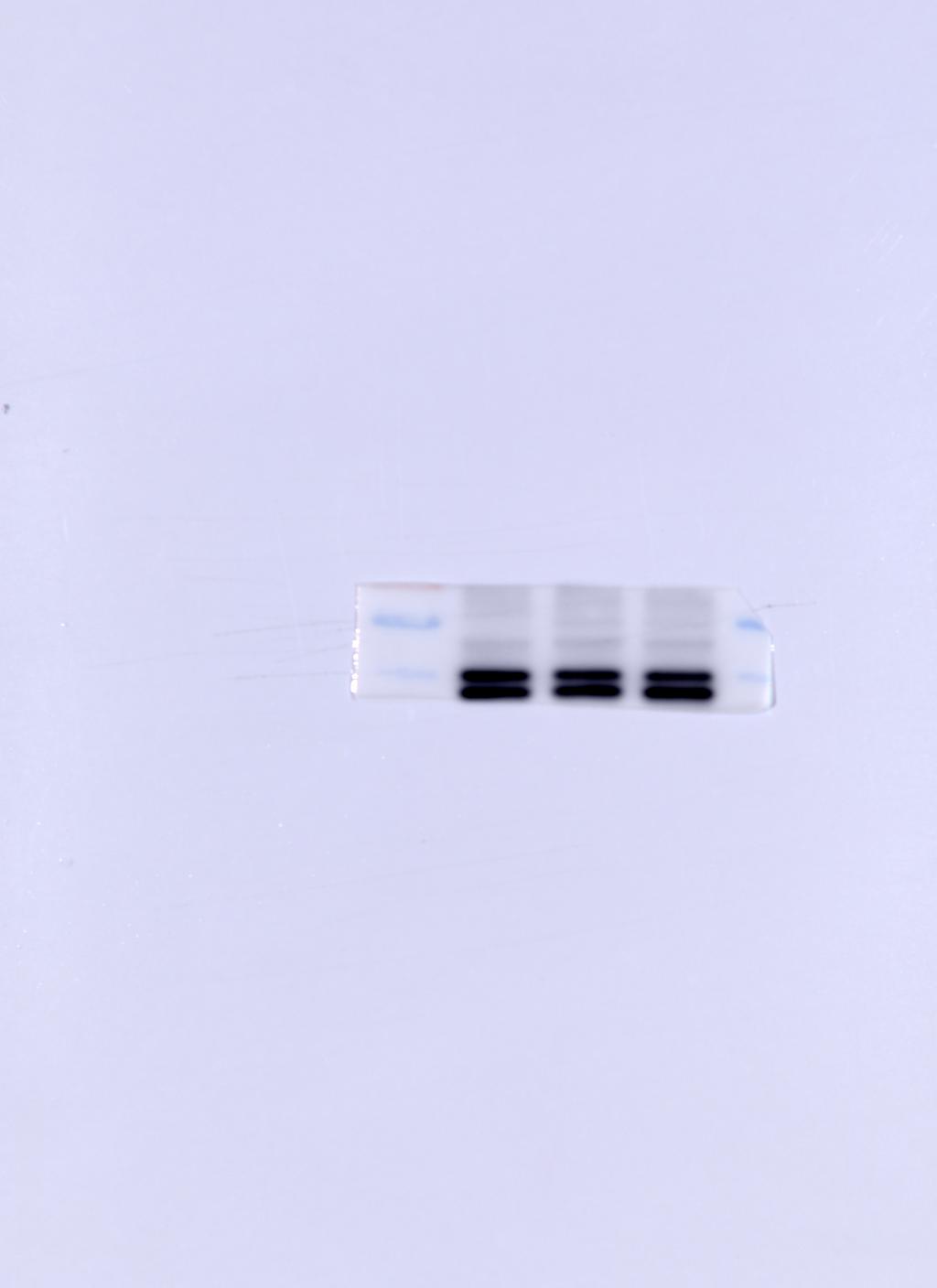

Supplement: Supplemental Information 2 — The CDF files for Fig. 2 are the data of analyzing the fingerprints of 10 batches of the rhizome of M. dauricum Polysaccharide. The software to open this file is the Similarity Evaluation System for Chromatographic Fingerprint of Traditional Chinese Medicine, and the software installation package is available in the Figure 2 folder. The OPJU file in the Figure 3 folder is the data of PCA analysis, and the open software is Origin, which can be downloaded and used for a fee from the Origin official website. [file peerj-10-13946-s002.zip › Raw date/Fig.6/p-ERK/3/ERK+Marker.jpg]

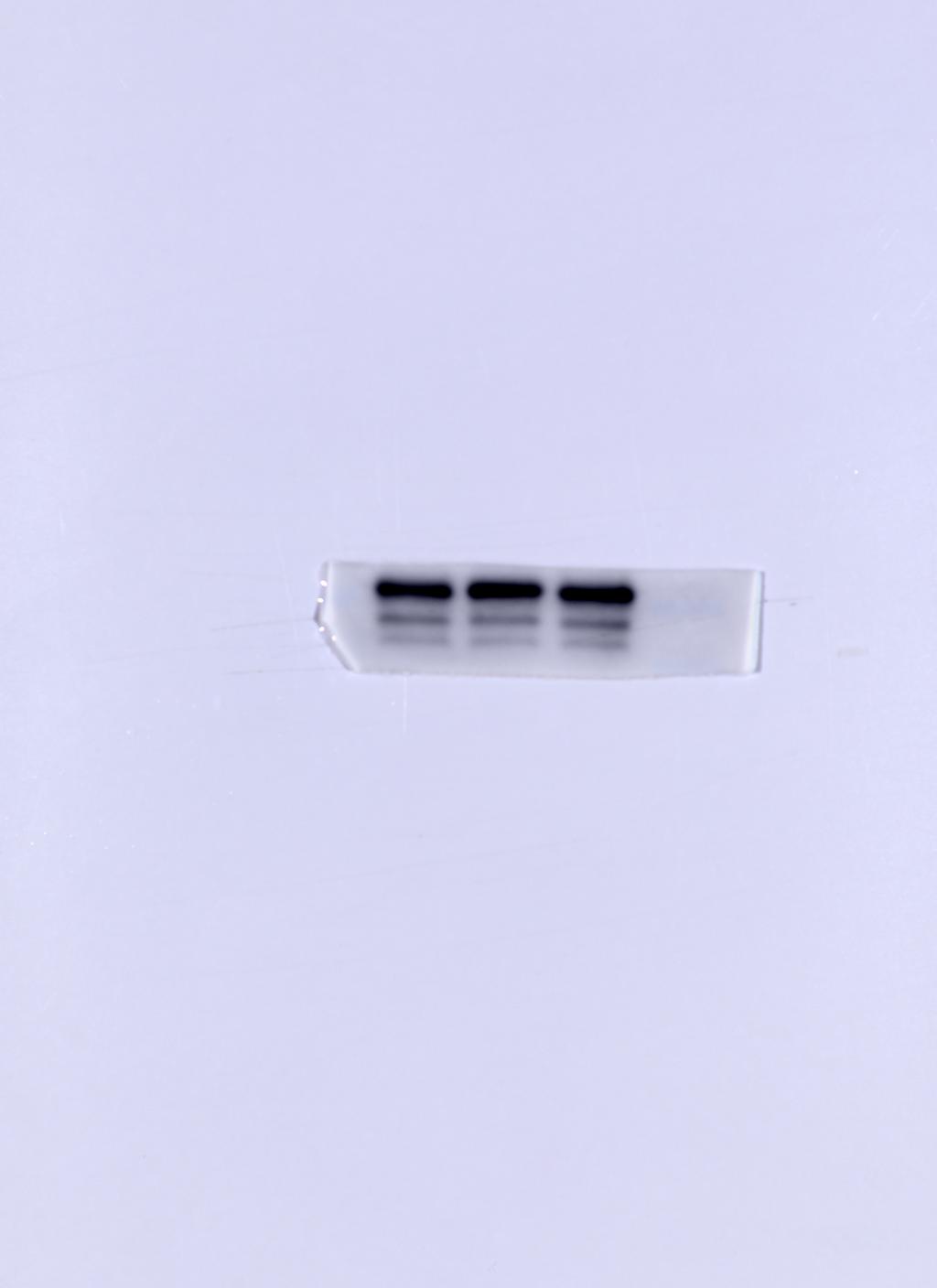

Supplement: Supplemental Information 2 — The CDF files for Fig. 2 are the data of analyzing the fingerprints of 10 batches of the rhizome of M. dauricum Polysaccharide. The software to open this file is the Similarity Evaluation System for Chromatographic Fingerprint of Traditional Chinese Medicine, and the software installation package is available in the Figure 2 folder. The OPJU file in the Figure 3 folder is the data of PCA analysis, and the open software is Origin, which can be downloaded and used for a fee from the Origin official website. [file peerj-10-13946-s002.zip › Raw date/Fig.6/p-ERK/3/ERK-GAPDH+Marker.jpg]

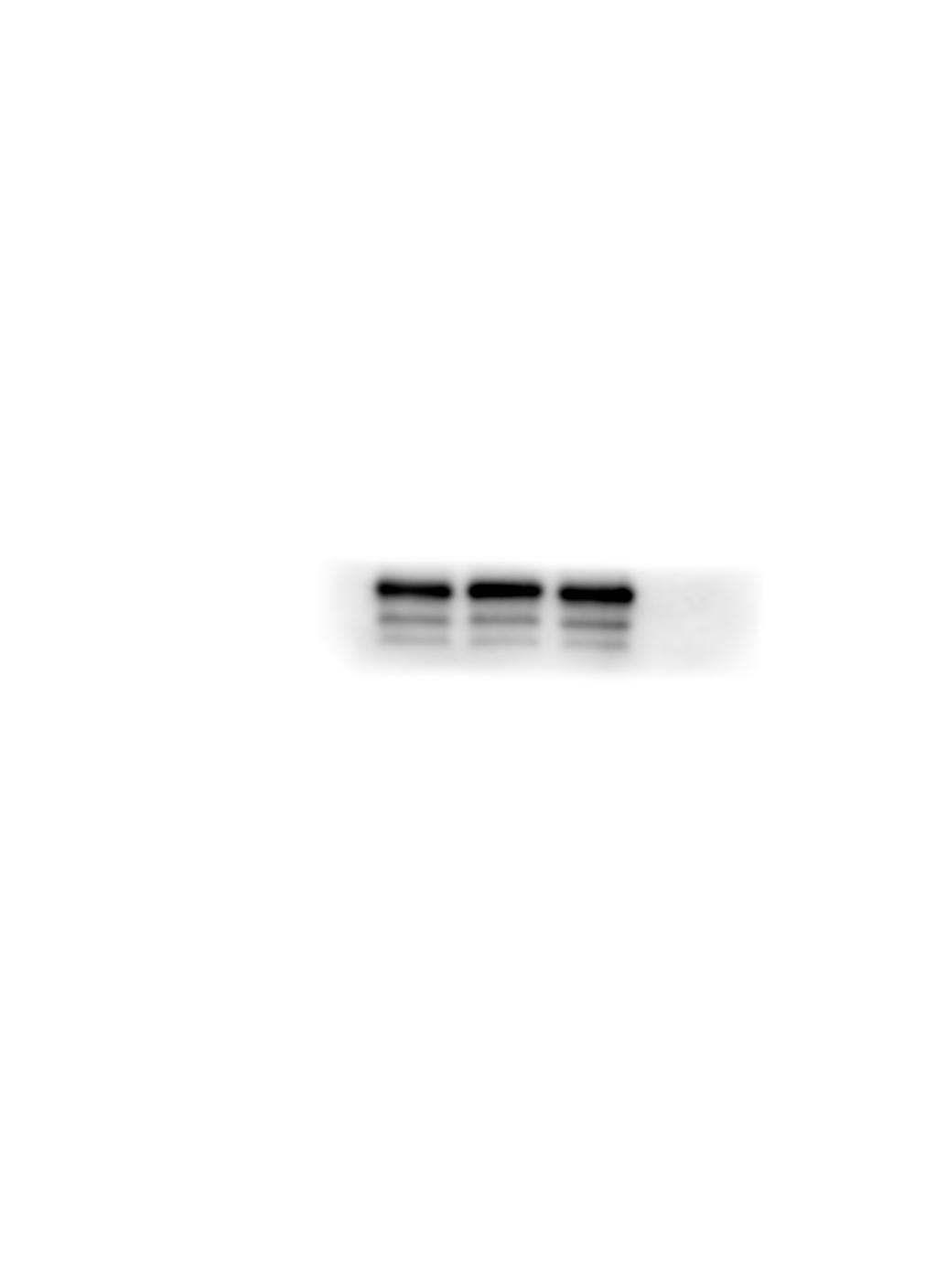

Supplement: Supplemental Information 2 — The CDF files for Fig. 2 are the data of analyzing the fingerprints of 10 batches of the rhizome of M. dauricum Polysaccharide. The software to open this file is the Similarity Evaluation System for Chromatographic Fingerprint of Traditional Chinese Medicine, and the software installation package is available in the Figure 2 folder. The OPJU file in the Figure 3 folder is the data of PCA analysis, and the open software is Origin, which can be downloaded and used for a fee from the Origin official website. [file peerj-10-13946-s002.zip › Raw date/Fig.6/p-ERK/3/ERK-GAPDH.jpg]

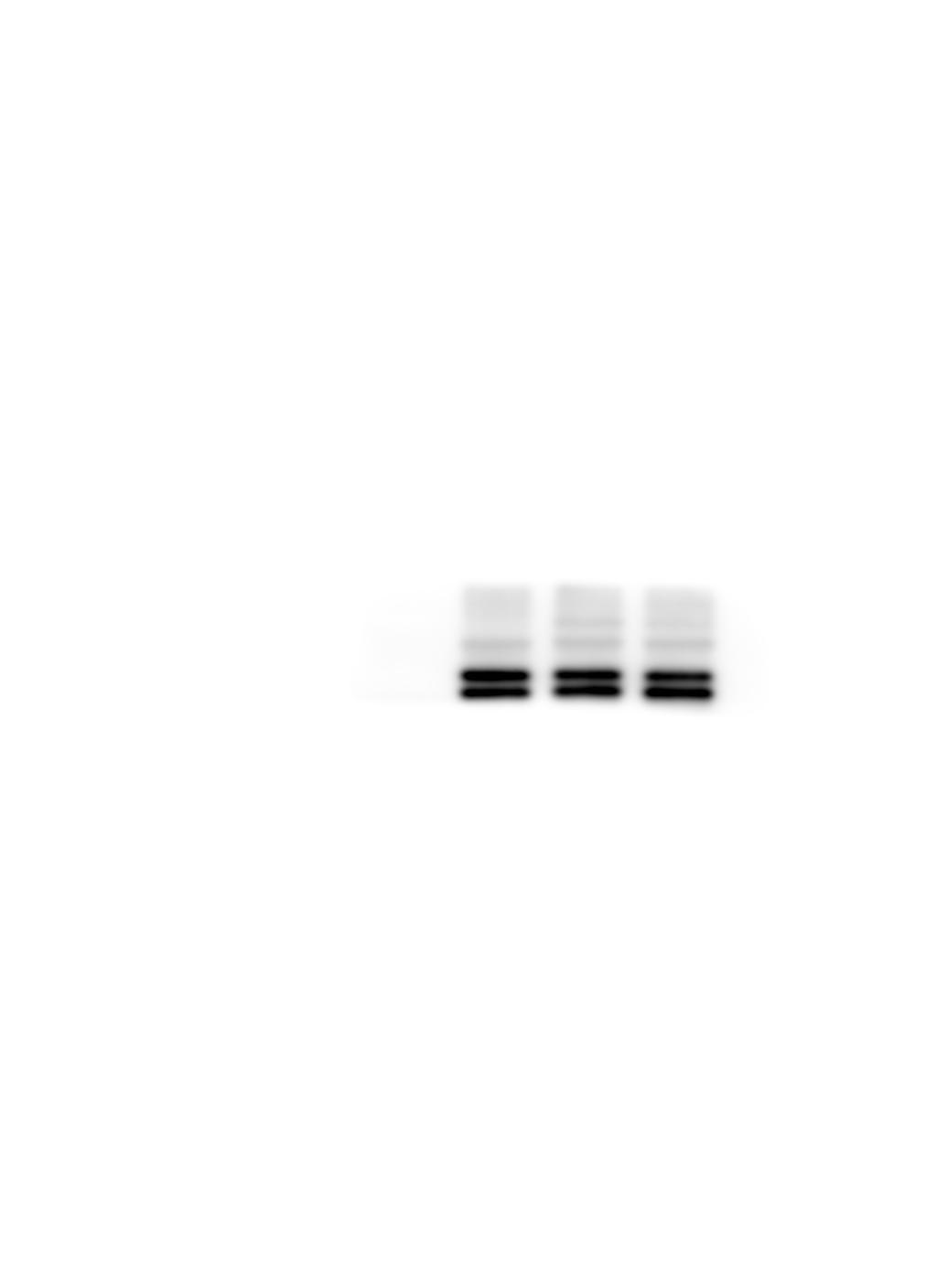

Supplement: Supplemental Information 2 — The CDF files for Fig. 2 are the data of analyzing the fingerprints of 10 batches of the rhizome of M. dauricum Polysaccharide. The software to open this file is the Similarity Evaluation System for Chromatographic Fingerprint of Traditional Chinese Medicine, and the software installation package is available in the Figure 2 folder. The OPJU file in the Figure 3 folder is the data of PCA analysis, and the open software is Origin, which can be downloaded and used for a fee from the Origin official website. [file peerj-10-13946-s002.zip › Raw date/Fig.6/p-ERK/3/ERK.jpg]

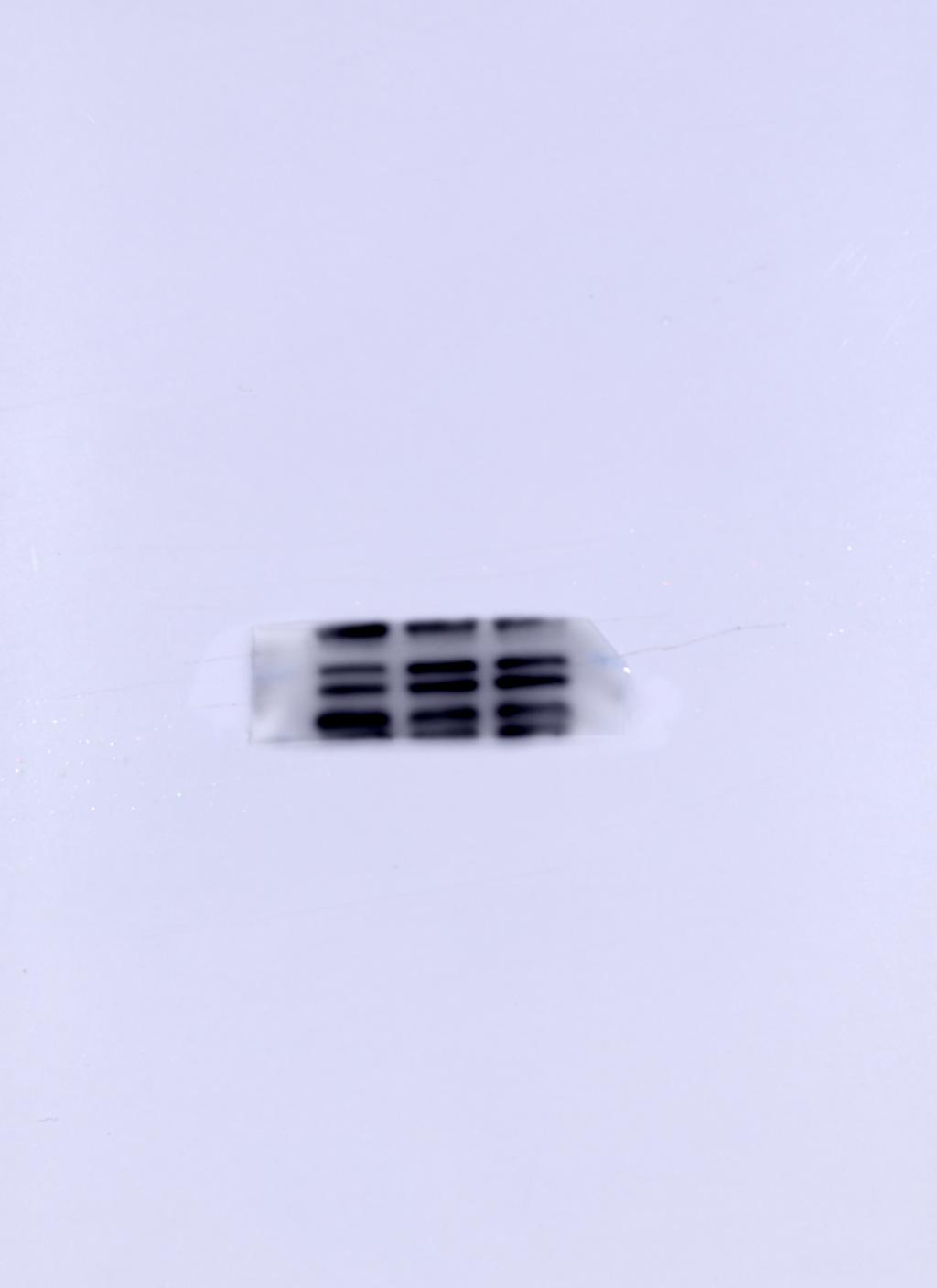

Supplement: Supplemental Information 2 — The CDF files for Fig. 2 are the data of analyzing the fingerprints of 10 batches of the rhizome of M. dauricum Polysaccharide. The software to open this file is the Similarity Evaluation System for Chromatographic Fingerprint of Traditional Chinese Medicine, and the software installation package is available in the Figure 2 folder. The OPJU file in the Figure 3 folder is the data of PCA analysis, and the open software is Origin, which can be downloaded and used for a fee from the Origin official website. [file peerj-10-13946-s002.zip › Raw date/Fig.6/p-ERK/3/p-ERK+Marker.jpg]

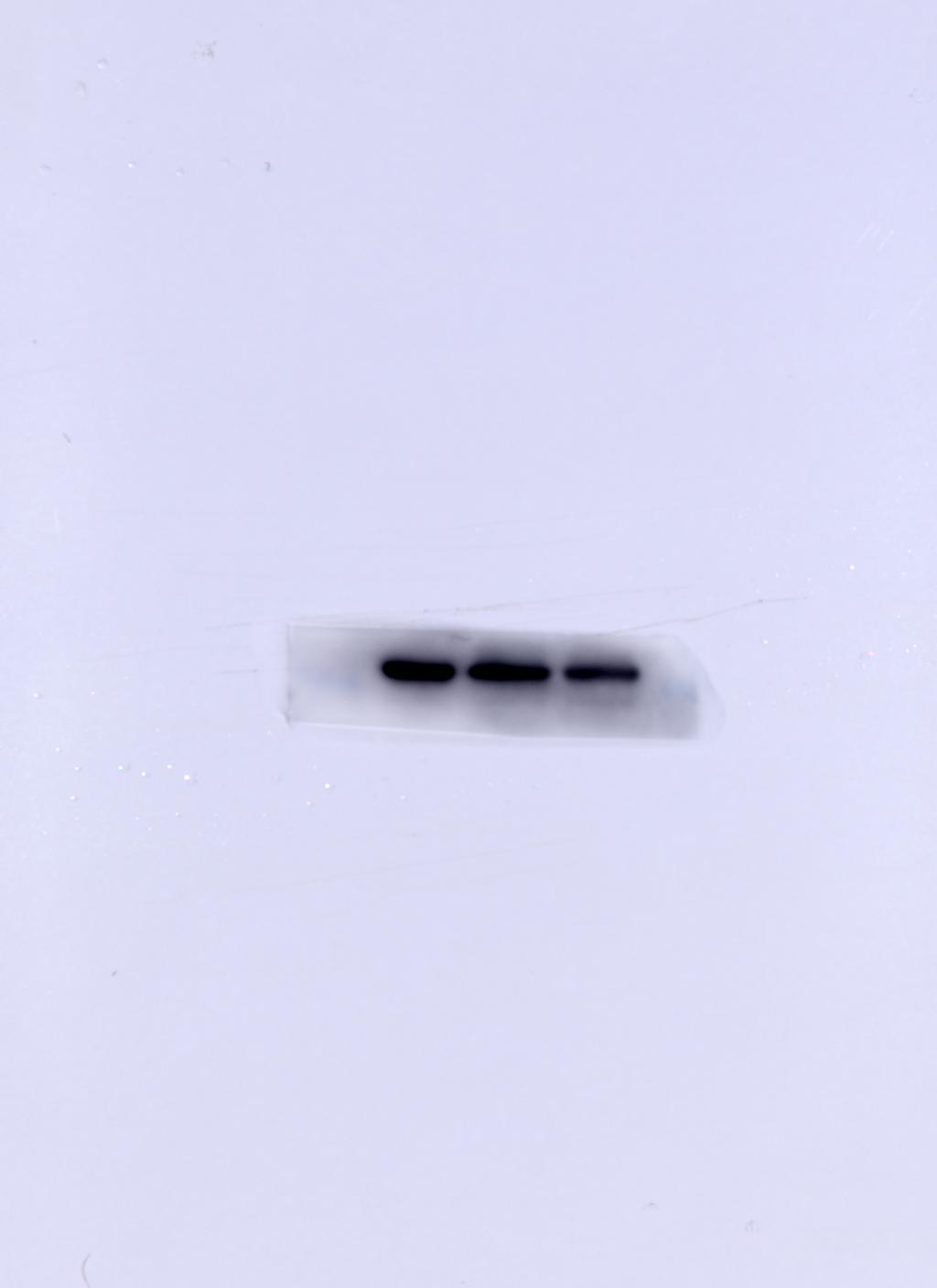

Supplement: Supplemental Information 2 — The CDF files for Fig. 2 are the data of analyzing the fingerprints of 10 batches of the rhizome of M. dauricum Polysaccharide. The software to open this file is the Similarity Evaluation System for Chromatographic Fingerprint of Traditional Chinese Medicine, and the software installation package is available in the Figure 2 folder. The OPJU file in the Figure 3 folder is the data of PCA analysis, and the open software is Origin, which can be downloaded and used for a fee from the Origin official website. [file peerj-10-13946-s002.zip › Raw date/Fig.6/p-ERK/3/p-ERK-GAPDH+Marker.jpg]

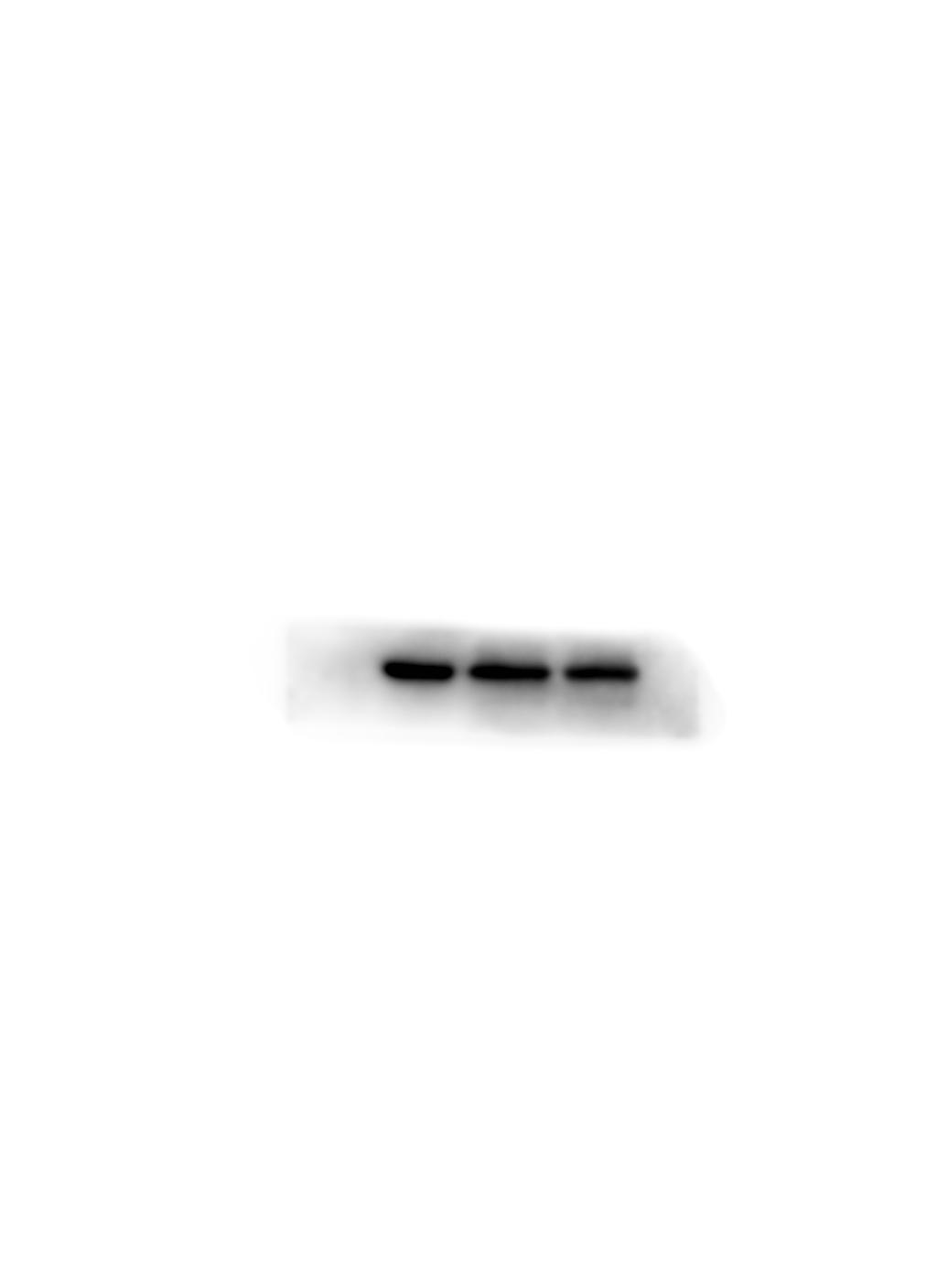

Supplement: Supplemental Information 2 — The CDF files for Fig. 2 are the data of analyzing the fingerprints of 10 batches of the rhizome of M. dauricum Polysaccharide. The software to open this file is the Similarity Evaluation System for Chromatographic Fingerprint of Traditional Chinese Medicine, and the software installation package is available in the Figure 2 folder. The OPJU file in the Figure 3 folder is the data of PCA analysis, and the open software is Origin, which can be downloaded and used for a fee from the Origin official website. [file peerj-10-13946-s002.zip › Raw date/Fig.6/p-ERK/3/p-ERK-GAPDH.jpg]

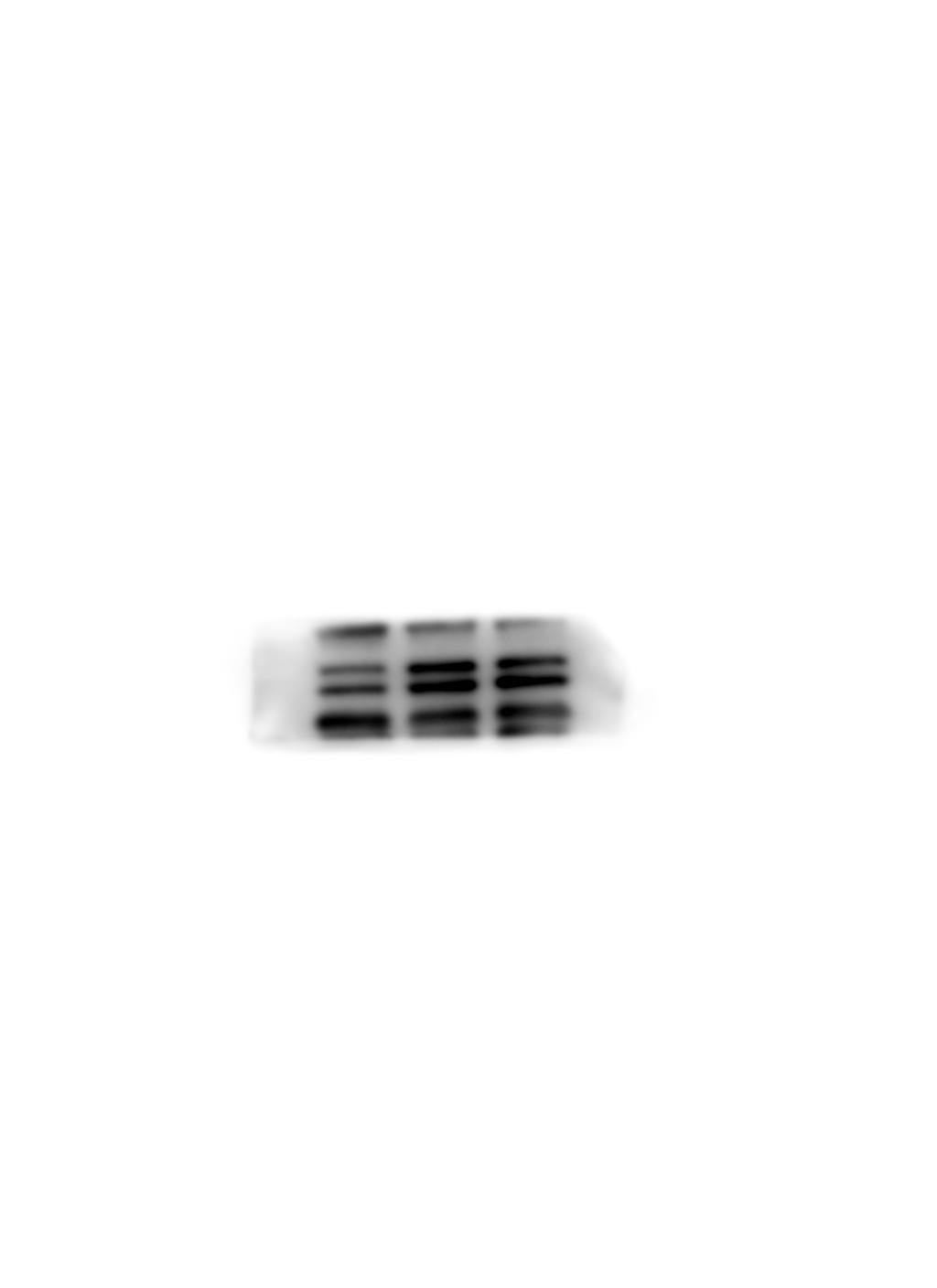

Supplement: Supplemental Information 2 — The CDF files for Fig. 2 are the data of analyzing the fingerprints of 10 batches of the rhizome of M. dauricum Polysaccharide. The software to open this file is the Similarity Evaluation System for Chromatographic Fingerprint of Traditional Chinese Medicine, and the software installation package is available in the Figure 2 folder. The OPJU file in the Figure 3 folder is the data of PCA analysis, and the open software is Origin, which can be downloaded and used for a fee from the Origin official website. [file peerj-10-13946-s002.zip › Raw date/Fig.6/p-ERK/3/p-ERK.jpg]

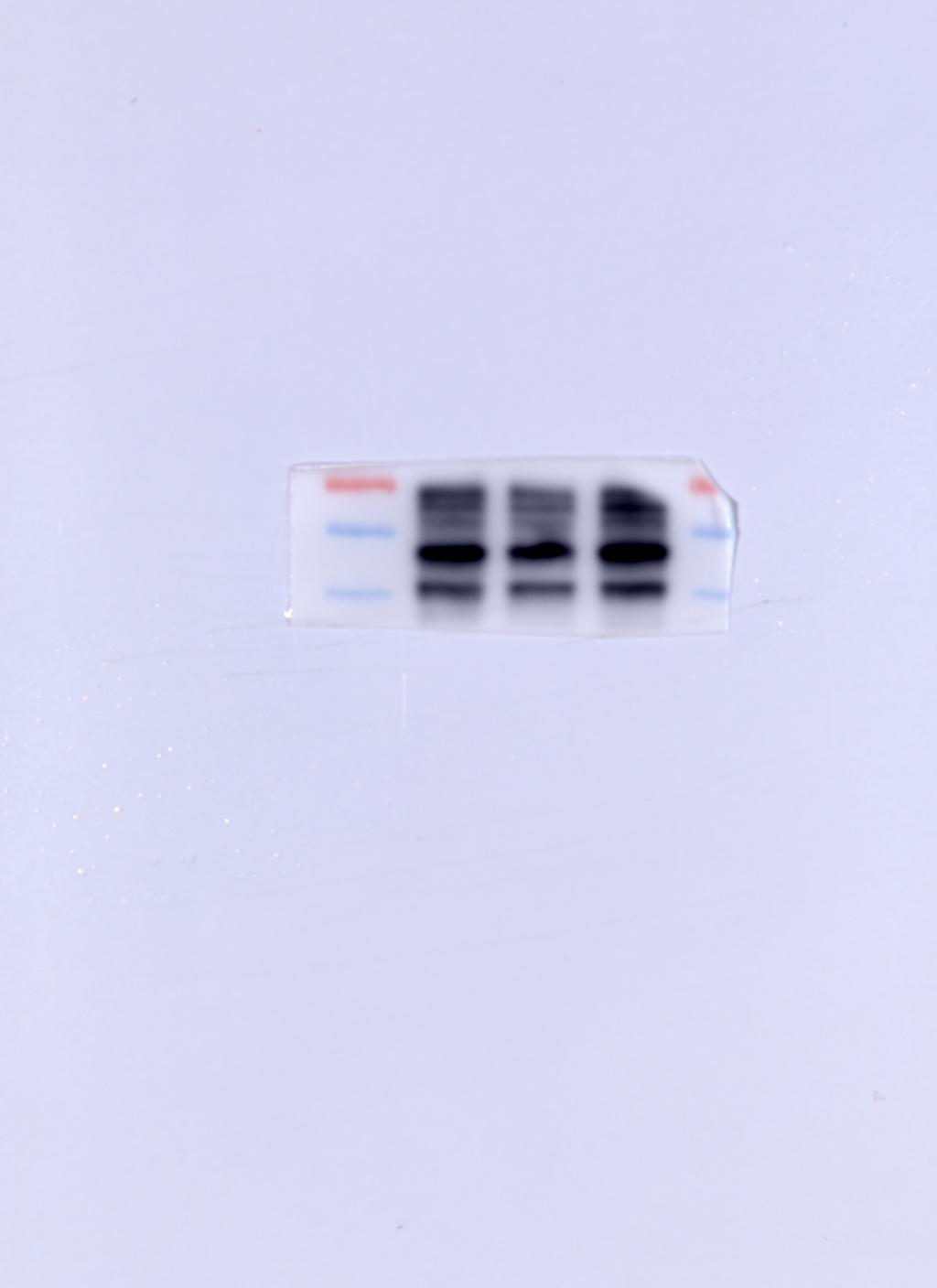

Supplement: Supplemental Information 2 — The CDF files for Fig. 2 are the data of analyzing the fingerprints of 10 batches of the rhizome of M. dauricum Polysaccharide. The software to open this file is the Similarity Evaluation System for Chromatographic Fingerprint of Traditional Chinese Medicine, and the software installation package is available in the Figure 2 folder. The OPJU file in the Figure 3 folder is the data of PCA analysis, and the open software is Origin, which can be downloaded and used for a fee from the Origin official website. [file peerj-10-13946-s002.zip › Raw date/Fig.6/p-JNK/1/JNK+Marker.jpg]

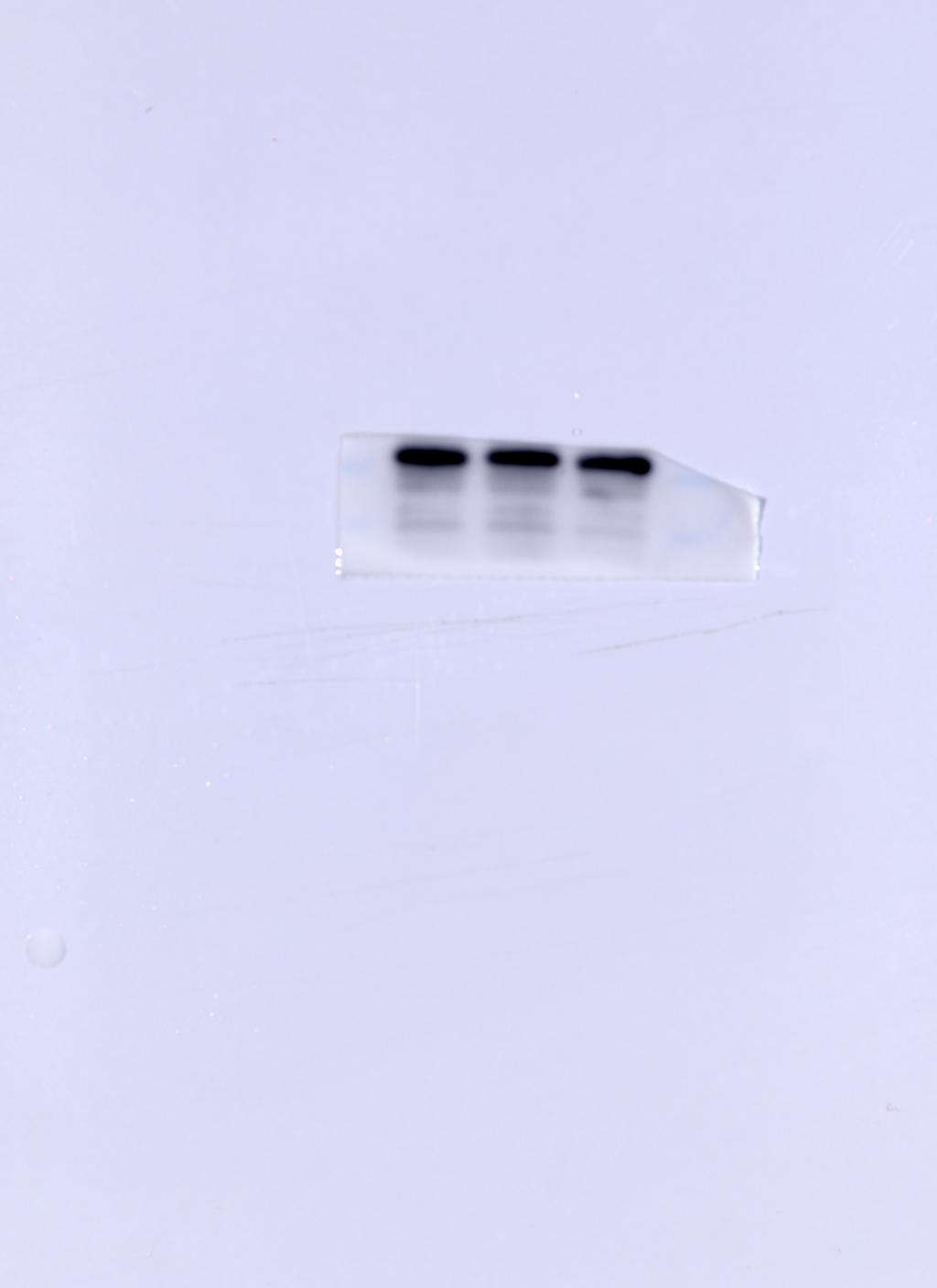

Supplement: Supplemental Information 2 — The CDF files for Fig. 2 are the data of analyzing the fingerprints of 10 batches of the rhizome of M. dauricum Polysaccharide. The software to open this file is the Similarity Evaluation System for Chromatographic Fingerprint of Traditional Chinese Medicine, and the software installation package is available in the Figure 2 folder. The OPJU file in the Figure 3 folder is the data of PCA analysis, and the open software is Origin, which can be downloaded and used for a fee from the Origin official website. [file peerj-10-13946-s002.zip › Raw date/Fig.6/p-JNK/1/JNK-GAPDH+Marker.jpg]

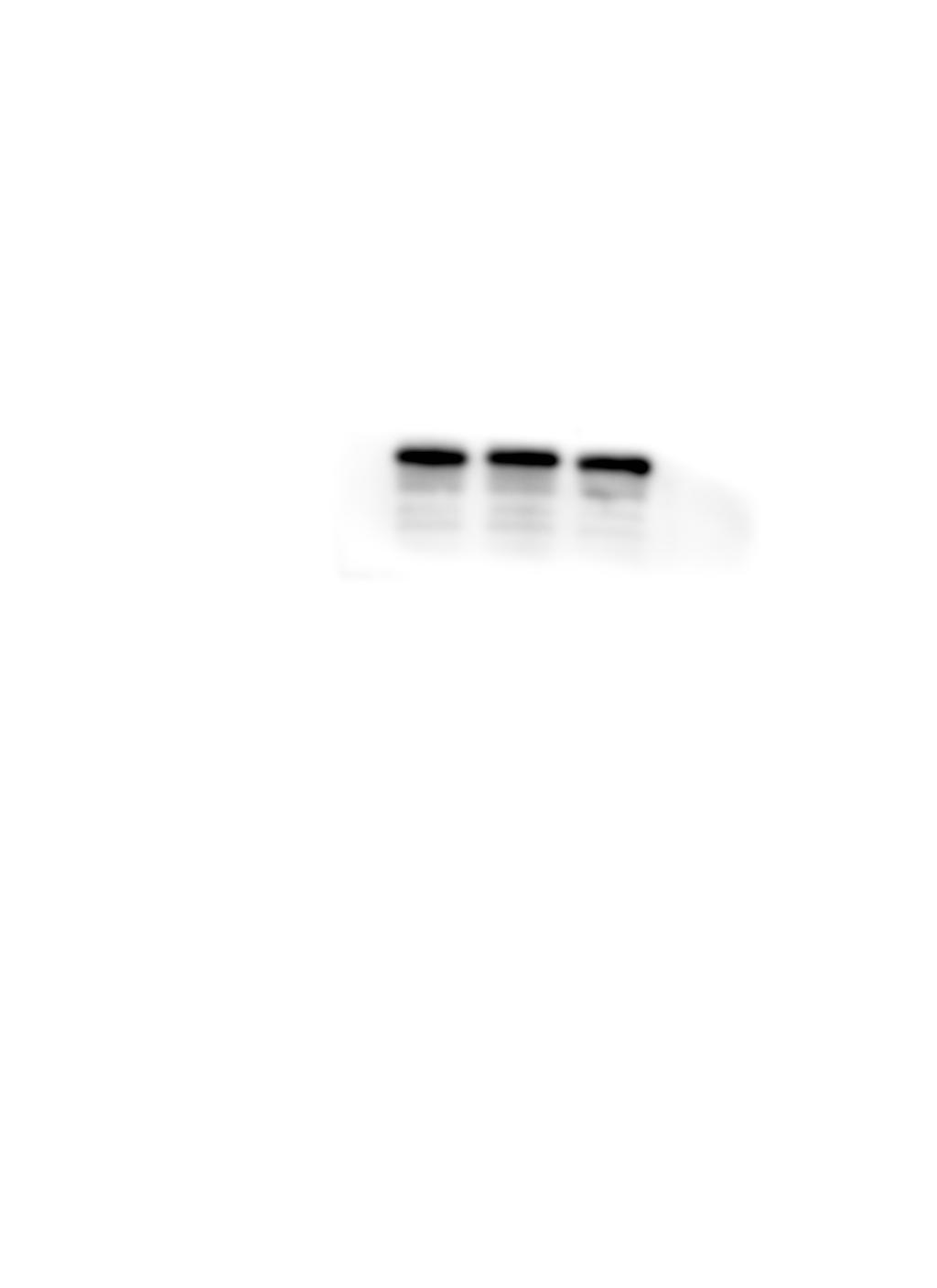

Supplement: Supplemental Information 2 — The CDF files for Fig. 2 are the data of analyzing the fingerprints of 10 batches of the rhizome of M. dauricum Polysaccharide. The software to open this file is the Similarity Evaluation System for Chromatographic Fingerprint of Traditional Chinese Medicine, and the software installation package is available in the Figure 2 folder. The OPJU file in the Figure 3 folder is the data of PCA analysis, and the open software is Origin, which can be downloaded and used for a fee from the Origin official website. [file peerj-10-13946-s002.zip › Raw date/Fig.6/p-JNK/1/JNK-GAPDH.jpg]

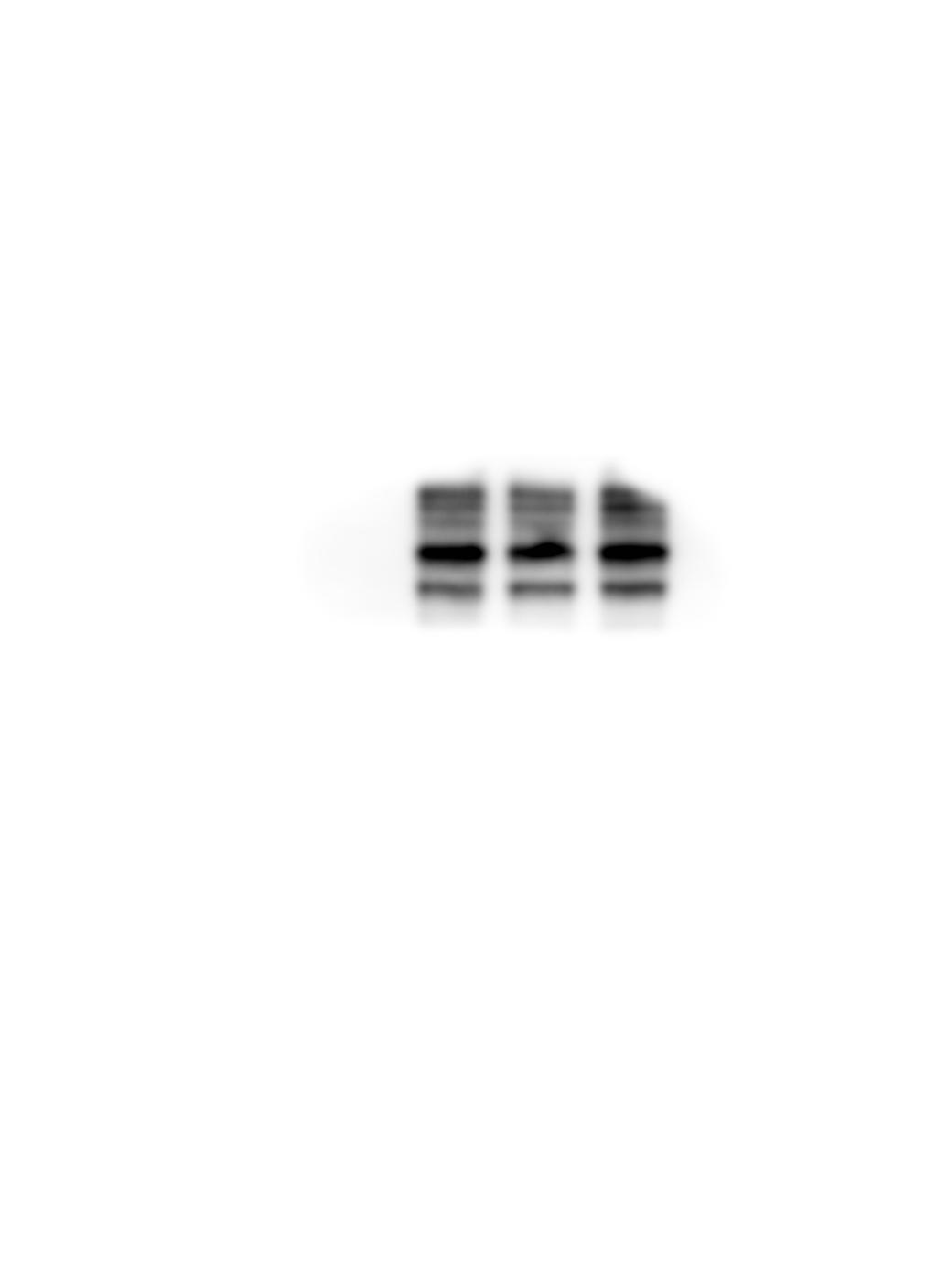

Supplement: Supplemental Information 2 — The CDF files for Fig. 2 are the data of analyzing the fingerprints of 10 batches of the rhizome of M. dauricum Polysaccharide. The software to open this file is the Similarity Evaluation System for Chromatographic Fingerprint of Traditional Chinese Medicine, and the software installation package is available in the Figure 2 folder. The OPJU file in the Figure 3 folder is the data of PCA analysis, and the open software is Origin, which can be downloaded and used for a fee from the Origin official website. [file peerj-10-13946-s002.zip › Raw date/Fig.6/p-JNK/1/JNK.jpg]

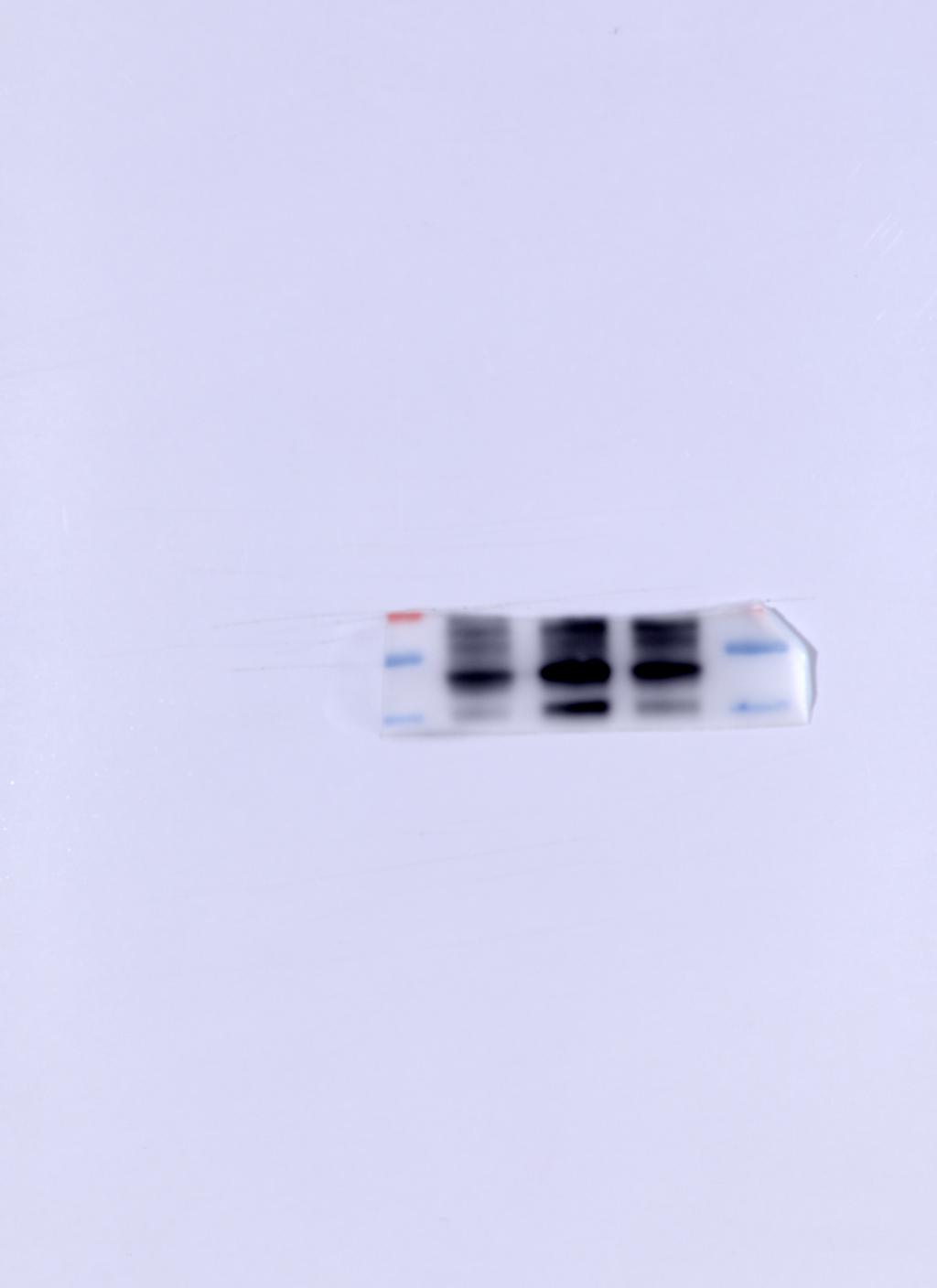

Supplement: Supplemental Information 2 — The CDF files for Fig. 2 are the data of analyzing the fingerprints of 10 batches of the rhizome of M. dauricum Polysaccharide. The software to open this file is the Similarity Evaluation System for Chromatographic Fingerprint of Traditional Chinese Medicine, and the software installation package is available in the Figure 2 folder. The OPJU file in the Figure 3 folder is the data of PCA analysis, and the open software is Origin, which can be downloaded and used for a fee from the Origin official website. [file peerj-10-13946-s002.zip › Raw date/Fig.6/p-JNK/1/p-JNK+Marker.jpg]

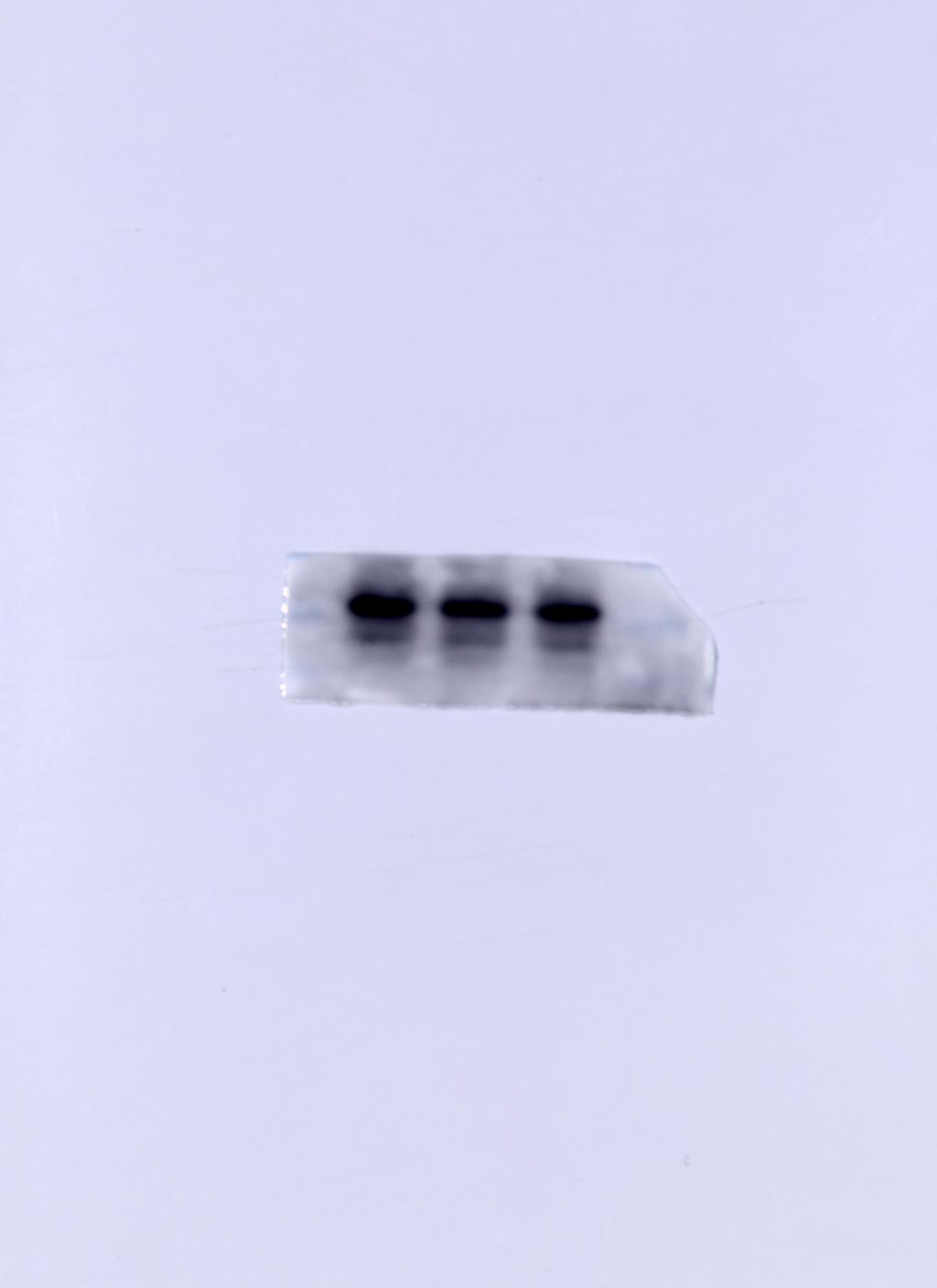

Supplement: Supplemental Information 2 — The CDF files for Fig. 2 are the data of analyzing the fingerprints of 10 batches of the rhizome of M. dauricum Polysaccharide. The software to open this file is the Similarity Evaluation System for Chromatographic Fingerprint of Traditional Chinese Medicine, and the software installation package is available in the Figure 2 folder. The OPJU file in the Figure 3 folder is the data of PCA analysis, and the open software is Origin, which can be downloaded and used for a fee from the Origin official website. [file peerj-10-13946-s002.zip › Raw date/Fig.6/p-JNK/1/p-JNK-GAPDH+Marker.jpg]

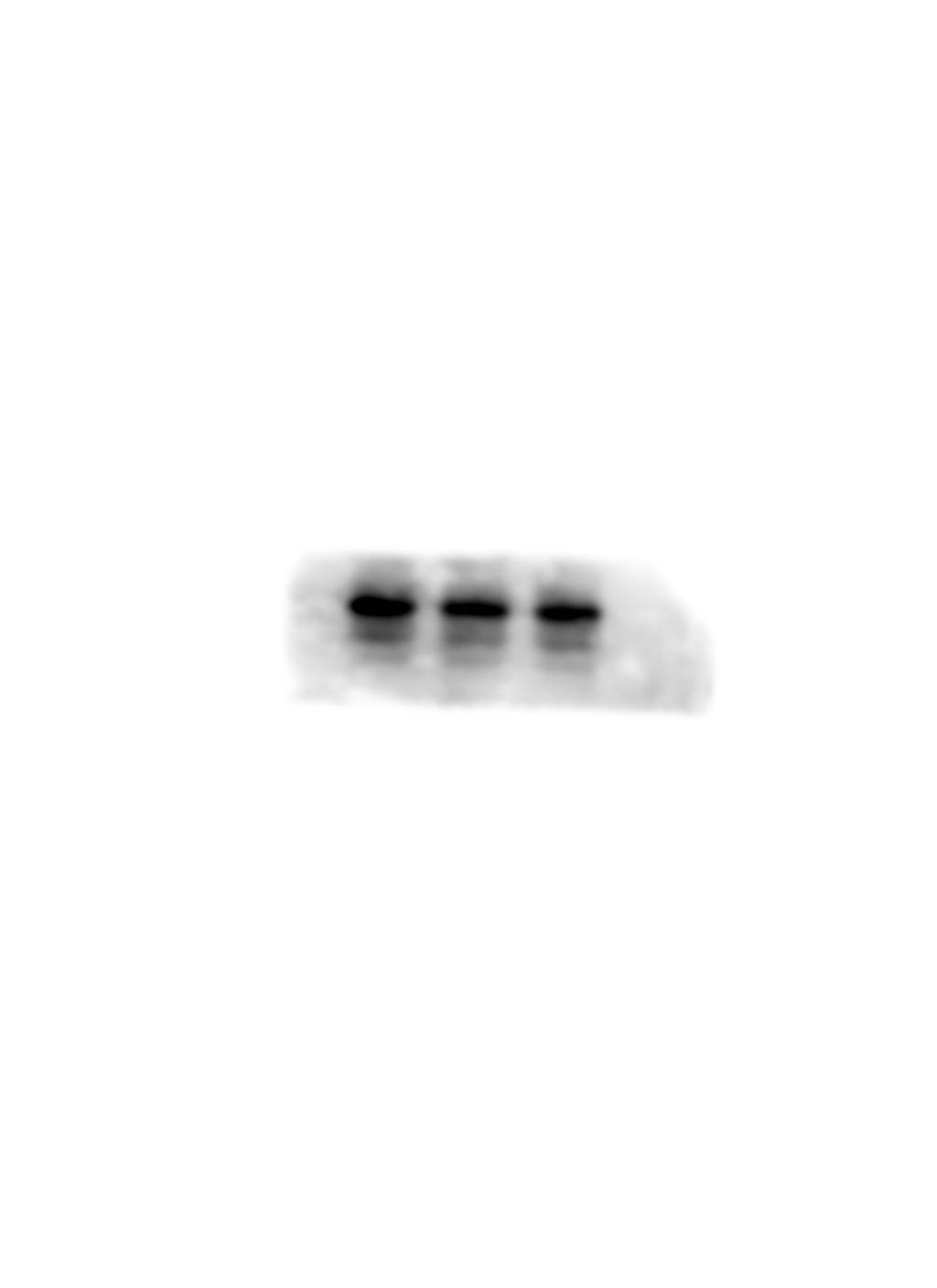

Supplement: Supplemental Information 2 — The CDF files for Fig. 2 are the data of analyzing the fingerprints of 10 batches of the rhizome of M. dauricum Polysaccharide. The software to open this file is the Similarity Evaluation System for Chromatographic Fingerprint of Traditional Chinese Medicine, and the software installation package is available in the Figure 2 folder. The OPJU file in the Figure 3 folder is the data of PCA analysis, and the open software is Origin, which can be downloaded and used for a fee from the Origin official website. [file peerj-10-13946-s002.zip › Raw date/Fig.6/p-JNK/1/p-JNK-GAPDH.jpg]

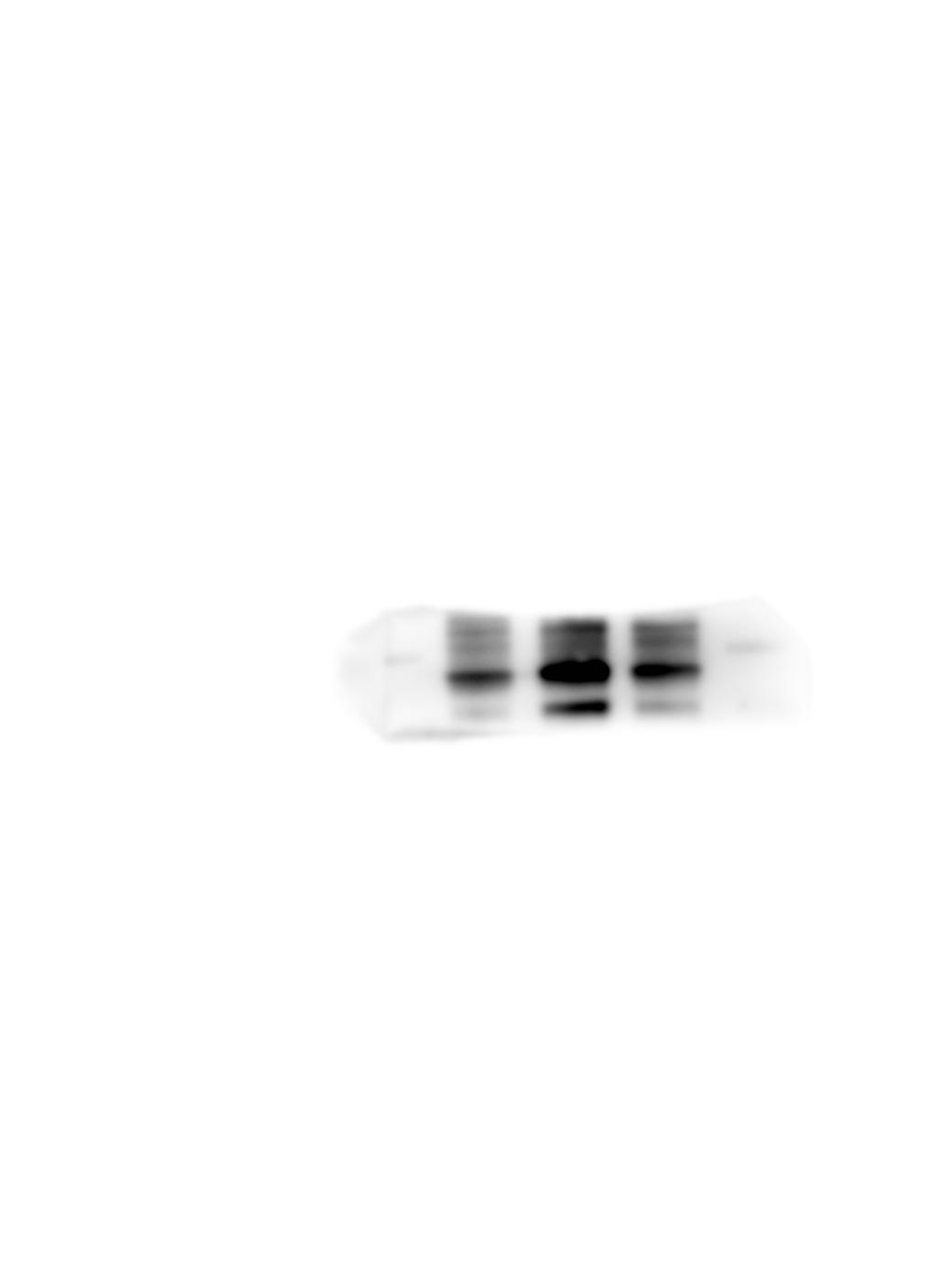

Supplement: Supplemental Information 2 — The CDF files for Fig. 2 are the data of analyzing the fingerprints of 10 batches of the rhizome of M. dauricum Polysaccharide. The software to open this file is the Similarity Evaluation System for Chromatographic Fingerprint of Traditional Chinese Medicine, and the software installation package is available in the Figure 2 folder. The OPJU file in the Figure 3 folder is the data of PCA analysis, and the open software is Origin, which can be downloaded and used for a fee from the Origin official website. [file peerj-10-13946-s002.zip › Raw date/Fig.6/p-JNK/1/p-JNK.jpg]

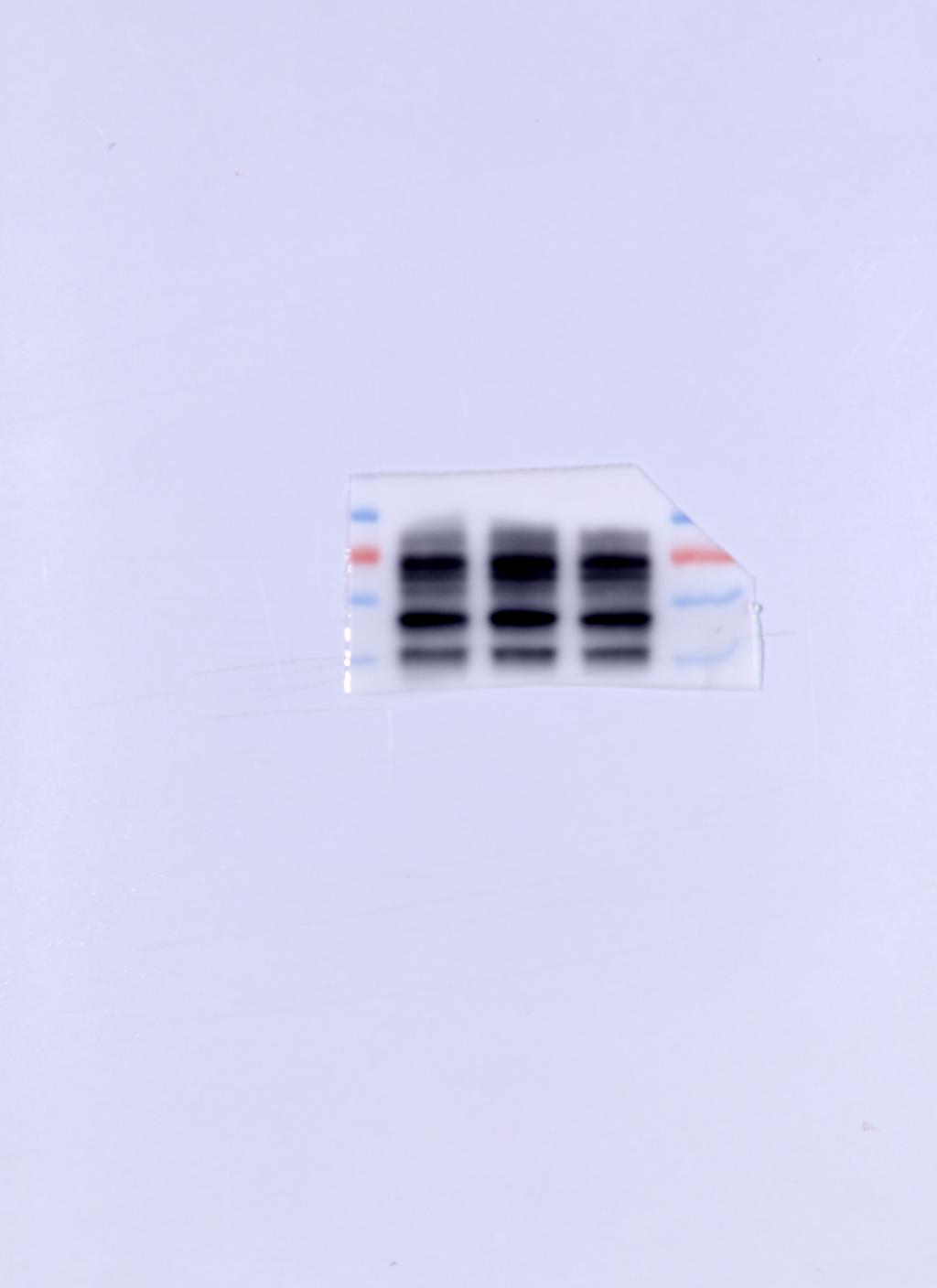

Supplement: Supplemental Information 2 — The CDF files for Fig. 2 are the data of analyzing the fingerprints of 10 batches of the rhizome of M. dauricum Polysaccharide. The software to open this file is the Similarity Evaluation System for Chromatographic Fingerprint of Traditional Chinese Medicine, and the software installation package is available in the Figure 2 folder. The OPJU file in the Figure 3 folder is the data of PCA analysis, and the open software is Origin, which can be downloaded and used for a fee from the Origin official website. [file peerj-10-13946-s002.zip › Raw date/Fig.6/p-JNK/2/JNK+Marker.jpg]

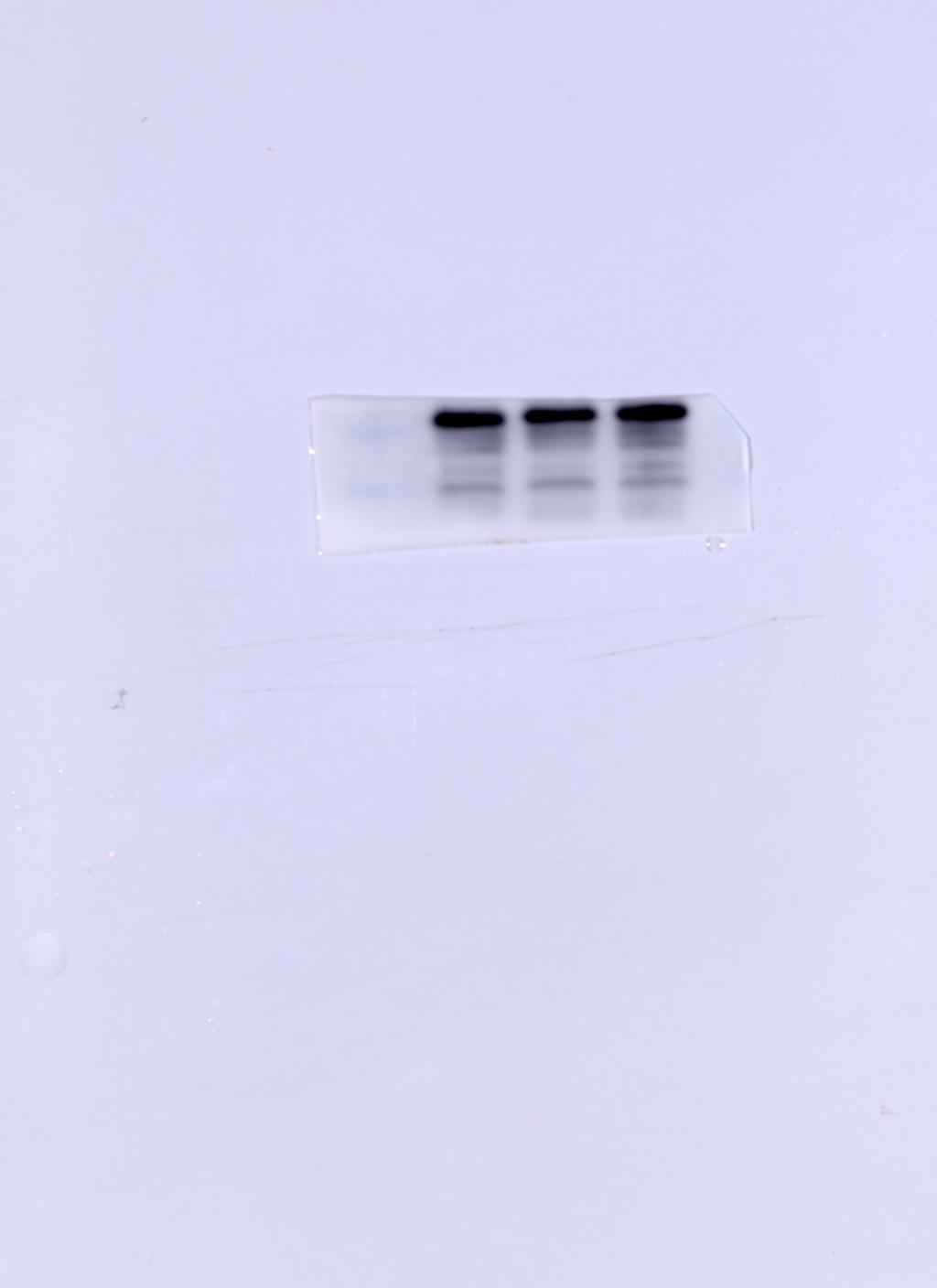

Supplement: Supplemental Information 2 — The CDF files for Fig. 2 are the data of analyzing the fingerprints of 10 batches of the rhizome of M. dauricum Polysaccharide. The software to open this file is the Similarity Evaluation System for Chromatographic Fingerprint of Traditional Chinese Medicine, and the software installation package is available in the Figure 2 folder. The OPJU file in the Figure 3 folder is the data of PCA analysis, and the open software is Origin, which can be downloaded and used for a fee from the Origin official website. [file peerj-10-13946-s002.zip › Raw date/Fig.6/p-JNK/2/JNK-GAPDH+Marker.jpg]

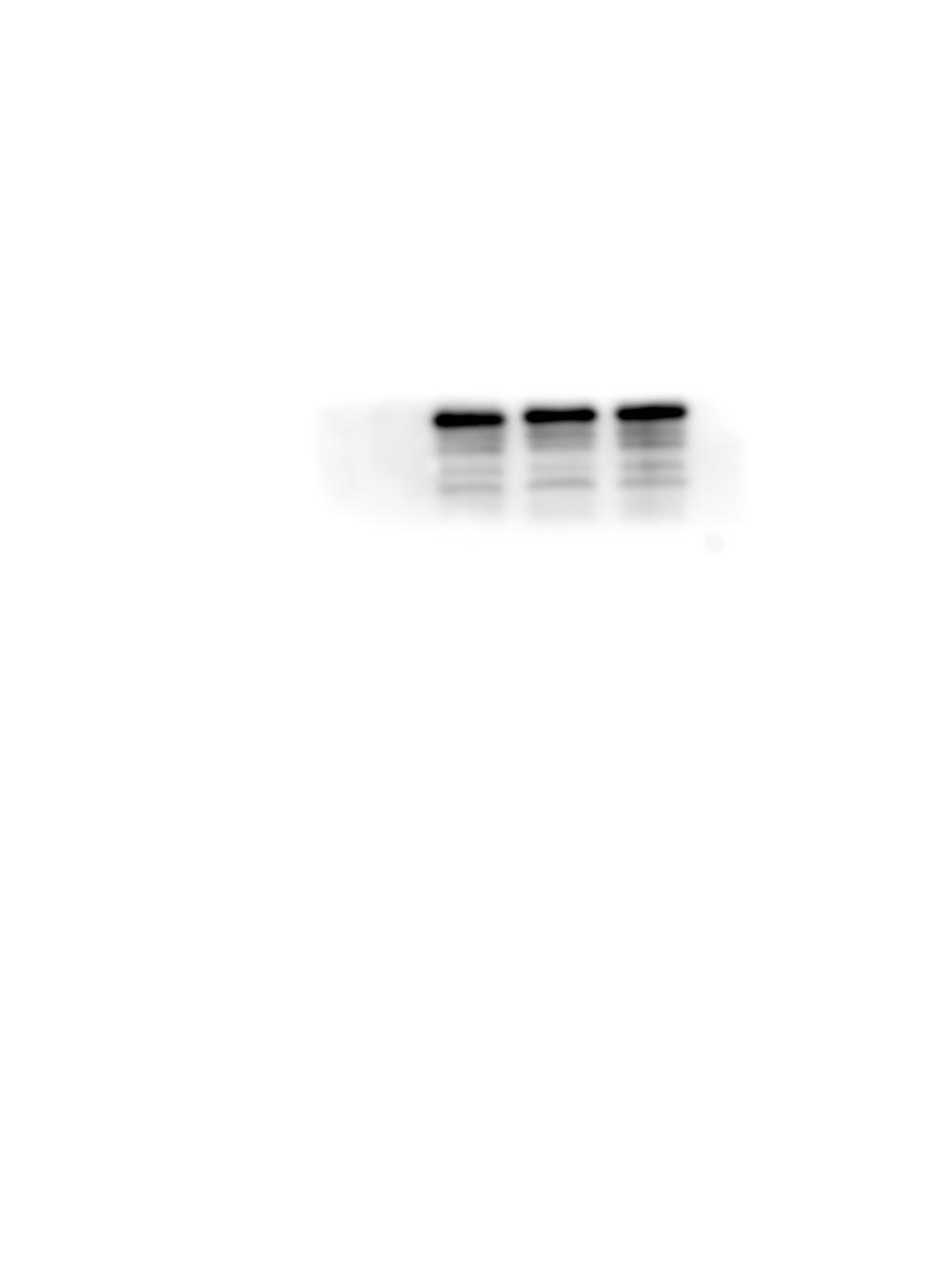

Supplement: Supplemental Information 2 — The CDF files for Fig. 2 are the data of analyzing the fingerprints of 10 batches of the rhizome of M. dauricum Polysaccharide. The software to open this file is the Similarity Evaluation System for Chromatographic Fingerprint of Traditional Chinese Medicine, and the software installation package is available in the Figure 2 folder. The OPJU file in the Figure 3 folder is the data of PCA analysis, and the open software is Origin, which can be downloaded and used for a fee from the Origin official website. [file peerj-10-13946-s002.zip › Raw date/Fig.6/p-JNK/2/JNK-GAPDH.jpg]

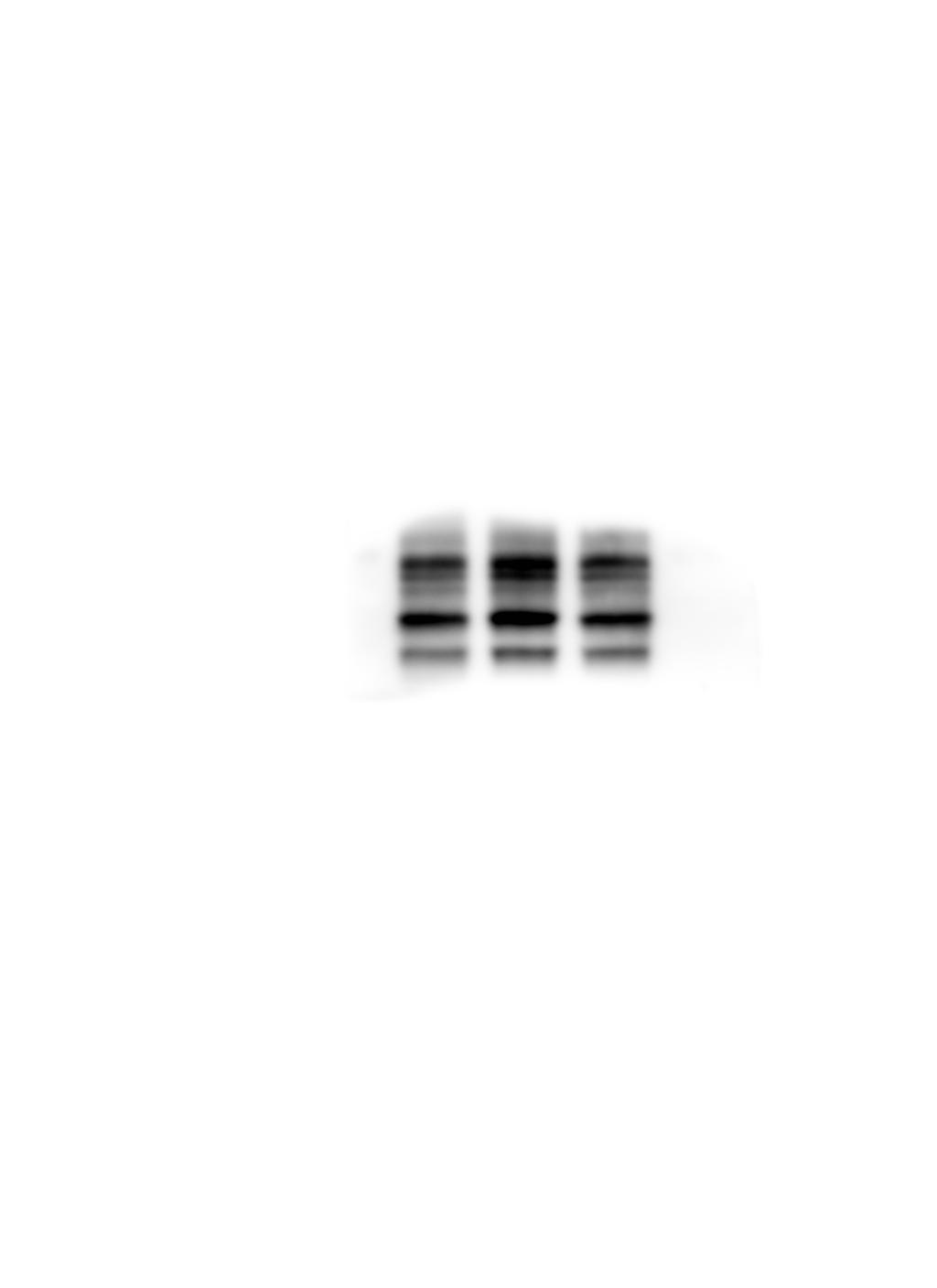

Supplement: Supplemental Information 2 — The CDF files for Fig. 2 are the data of analyzing the fingerprints of 10 batches of the rhizome of M. dauricum Polysaccharide. The software to open this file is the Similarity Evaluation System for Chromatographic Fingerprint of Traditional Chinese Medicine, and the software installation package is available in the Figure 2 folder. The OPJU file in the Figure 3 folder is the data of PCA analysis, and the open software is Origin, which can be downloaded and used for a fee from the Origin official website. [file peerj-10-13946-s002.zip › Raw date/Fig.6/p-JNK/2/JNK.jpg]

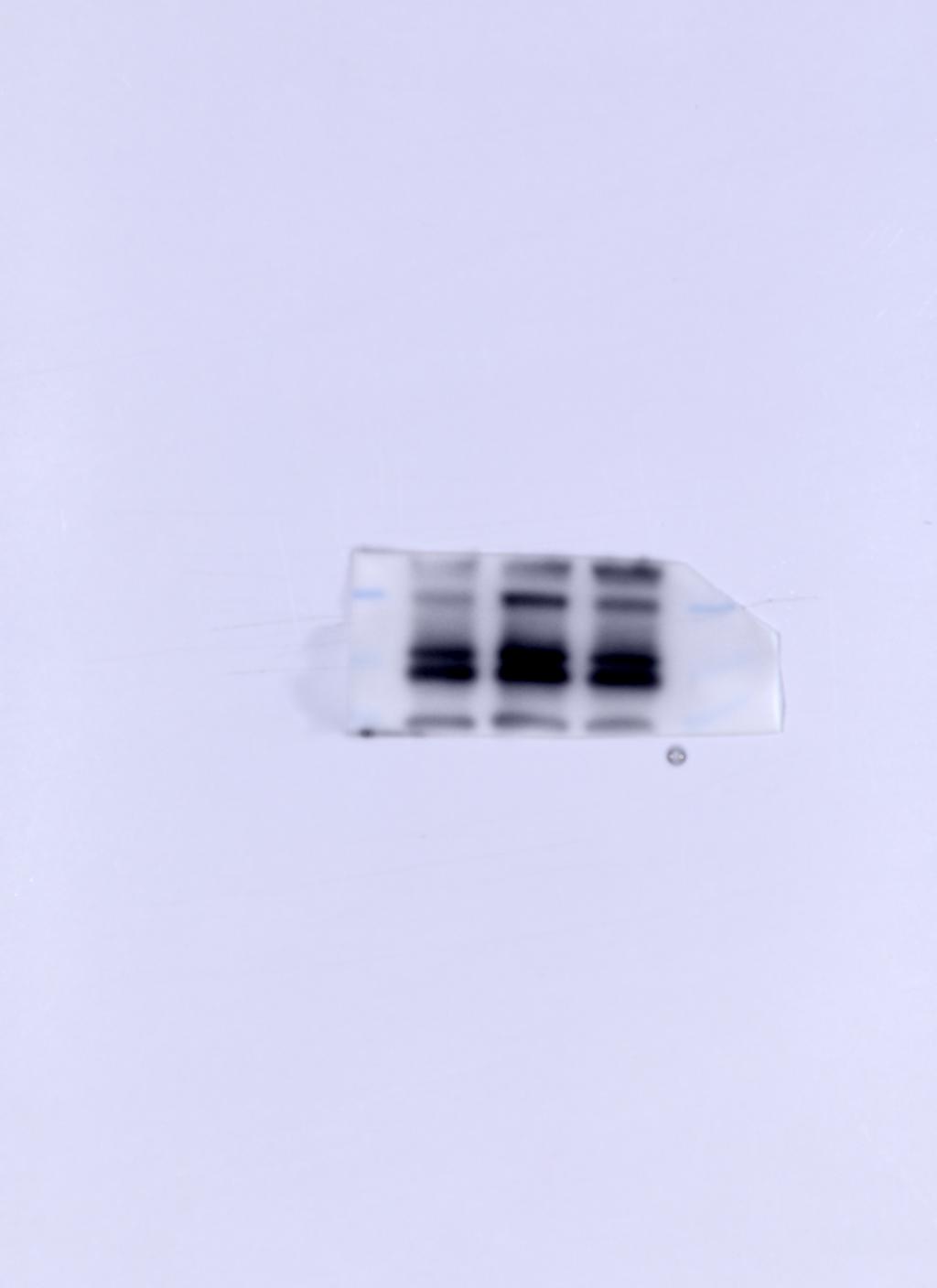

Supplement: Supplemental Information 2 — The CDF files for Fig. 2 are the data of analyzing the fingerprints of 10 batches of the rhizome of M. dauricum Polysaccharide. The software to open this file is the Similarity Evaluation System for Chromatographic Fingerprint of Traditional Chinese Medicine, and the software installation package is available in the Figure 2 folder. The OPJU file in the Figure 3 folder is the data of PCA analysis, and the open software is Origin, which can be downloaded and used for a fee from the Origin official website. [file peerj-10-13946-s002.zip › Raw date/Fig.6/p-JNK/2/p-JNK+Marker.jpg]

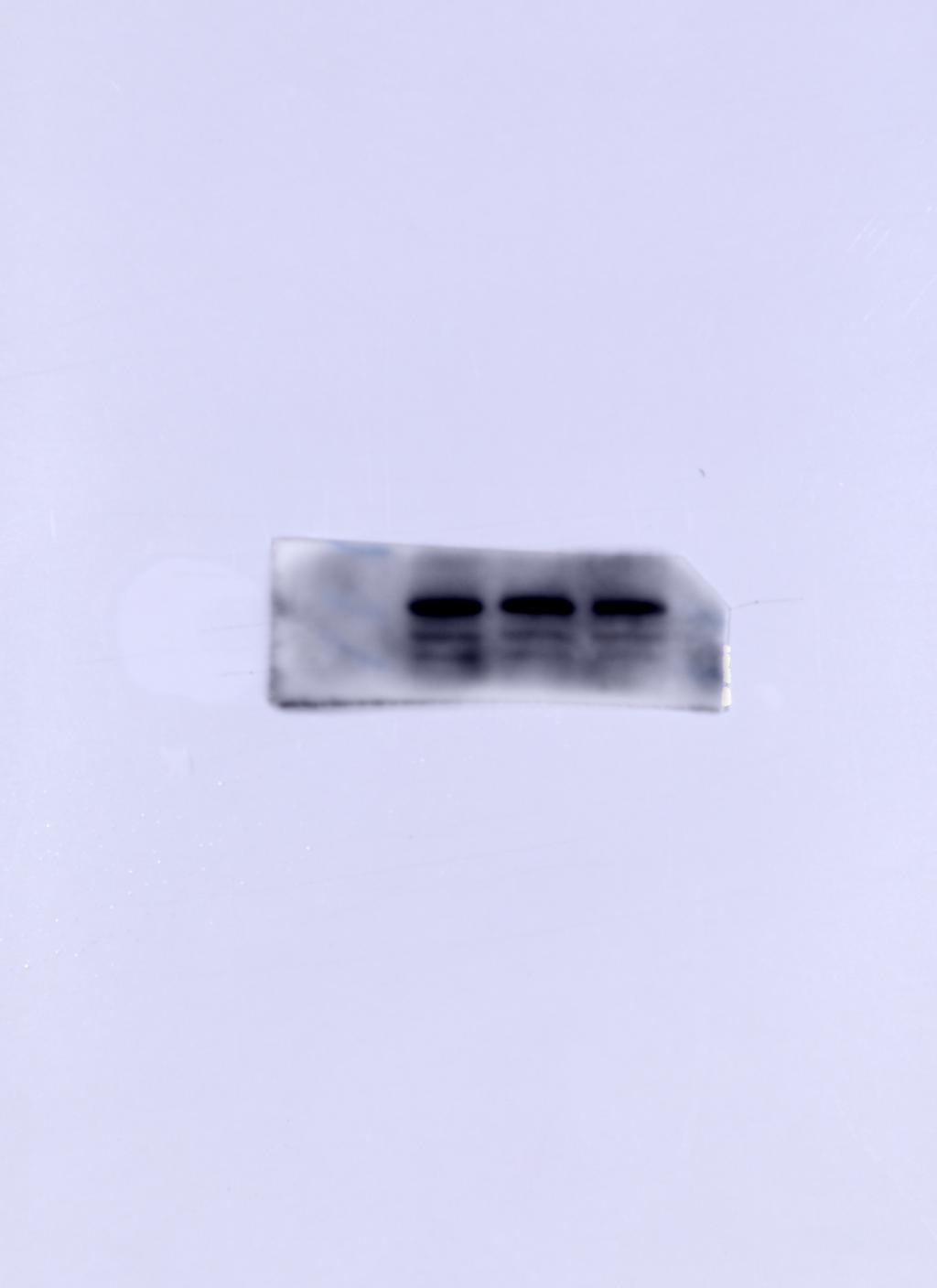

Supplement: Supplemental Information 2 — The CDF files for Fig. 2 are the data of analyzing the fingerprints of 10 batches of the rhizome of M. dauricum Polysaccharide. The software to open this file is the Similarity Evaluation System for Chromatographic Fingerprint of Traditional Chinese Medicine, and the software installation package is available in the Figure 2 folder. The OPJU file in the Figure 3 folder is the data of PCA analysis, and the open software is Origin, which can be downloaded and used for a fee from the Origin official website. [file peerj-10-13946-s002.zip › Raw date/Fig.6/p-JNK/2/p-JNK-GAPDH+Marker.jpg]

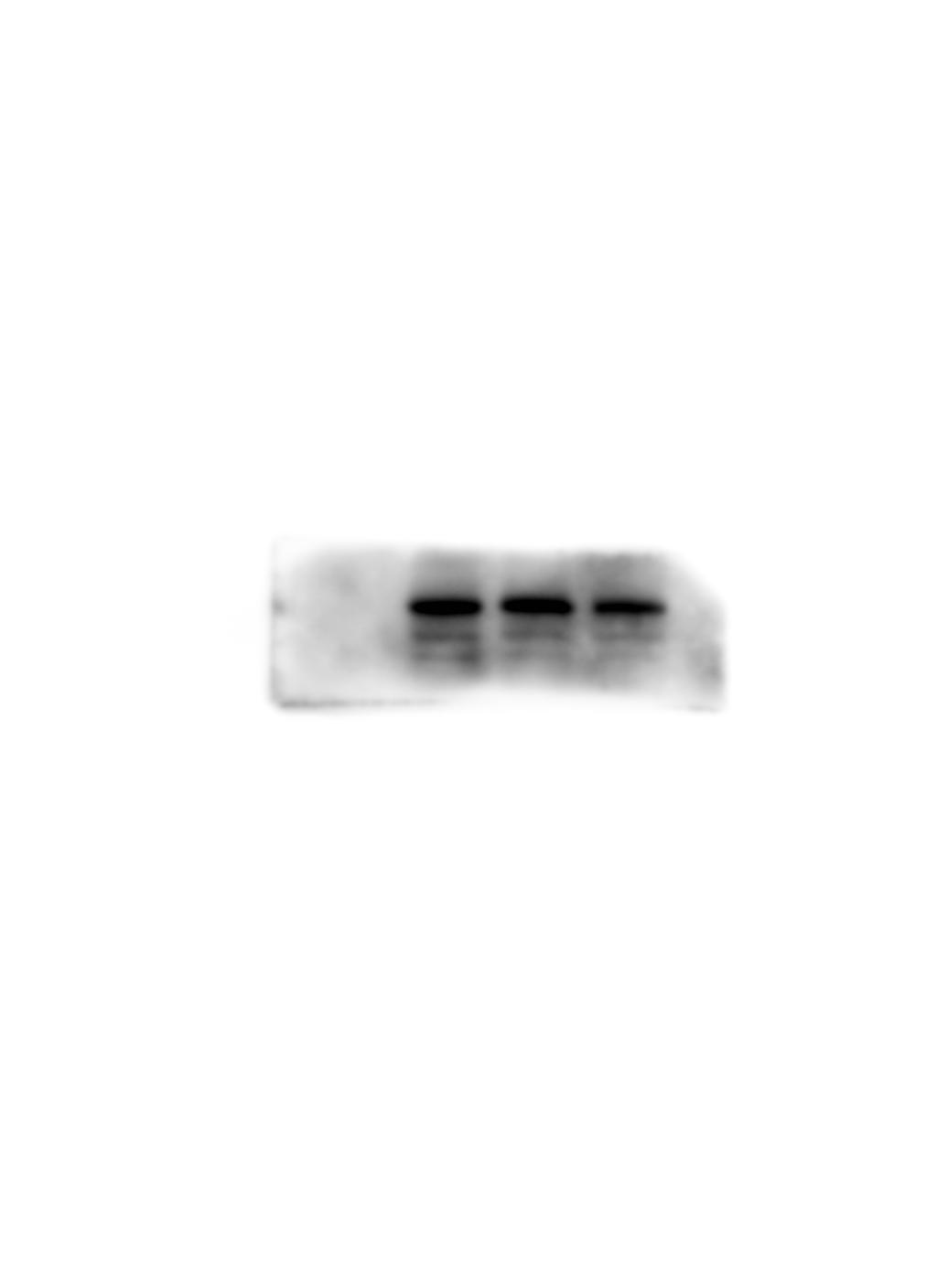

Supplement: Supplemental Information 2 — The CDF files for Fig. 2 are the data of analyzing the fingerprints of 10 batches of the rhizome of M. dauricum Polysaccharide. The software to open this file is the Similarity Evaluation System for Chromatographic Fingerprint of Traditional Chinese Medicine, and the software installation package is available in the Figure 2 folder. The OPJU file in the Figure 3 folder is the data of PCA analysis, and the open software is Origin, which can be downloaded and used for a fee from the Origin official website. [file peerj-10-13946-s002.zip › Raw date/Fig.6/p-JNK/2/p-JNK-GAPDH.jpg]

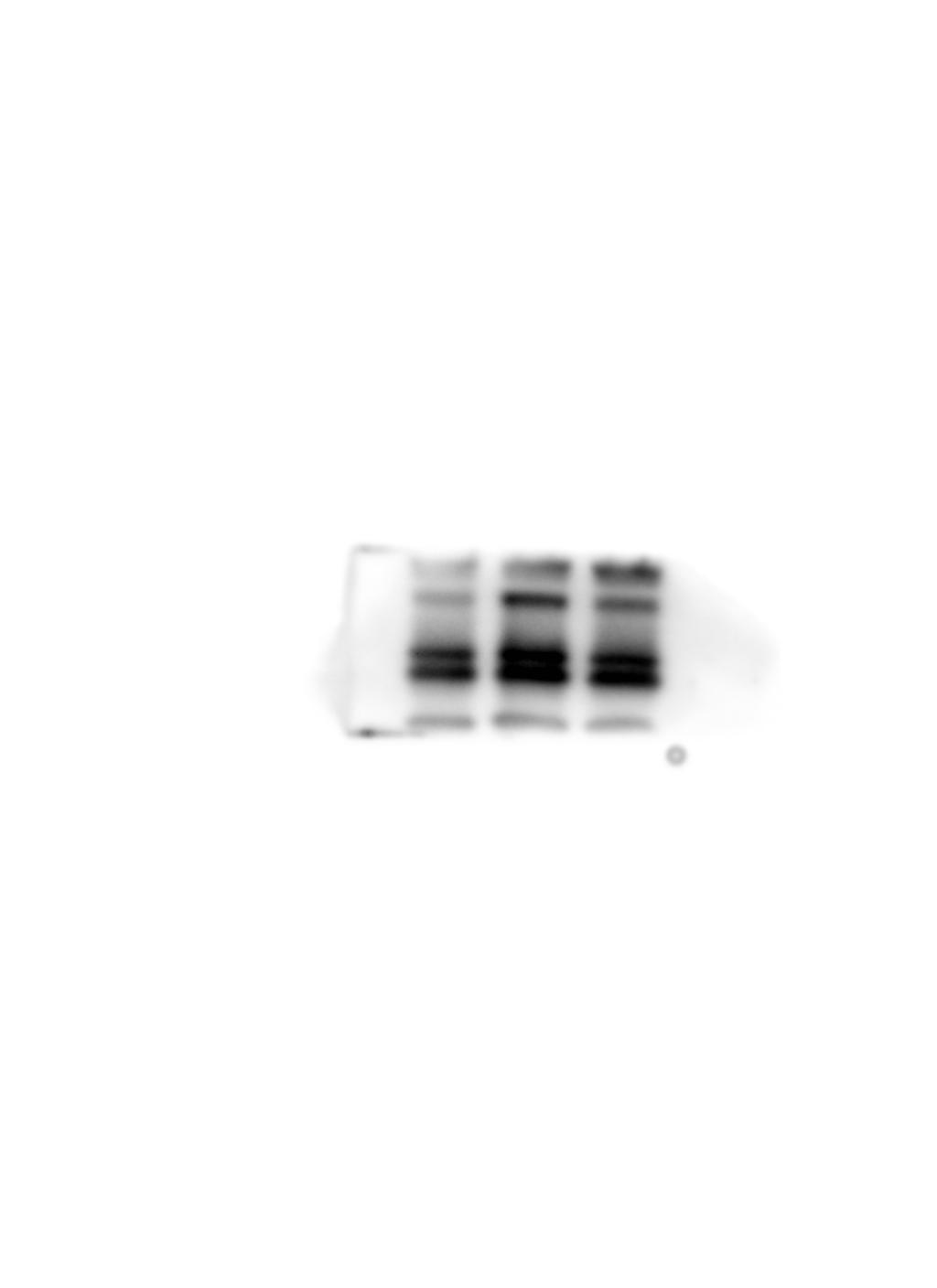

Supplement: Supplemental Information 2 — The CDF files for Fig. 2 are the data of analyzing the fingerprints of 10 batches of the rhizome of M. dauricum Polysaccharide. The software to open this file is the Similarity Evaluation System for Chromatographic Fingerprint of Traditional Chinese Medicine, and the software installation package is available in the Figure 2 folder. The OPJU file in the Figure 3 folder is the data of PCA analysis, and the open software is Origin, which can be downloaded and used for a fee from the Origin official website. [file peerj-10-13946-s002.zip › Raw date/Fig.6/p-JNK/2/p-JNK.jpg]

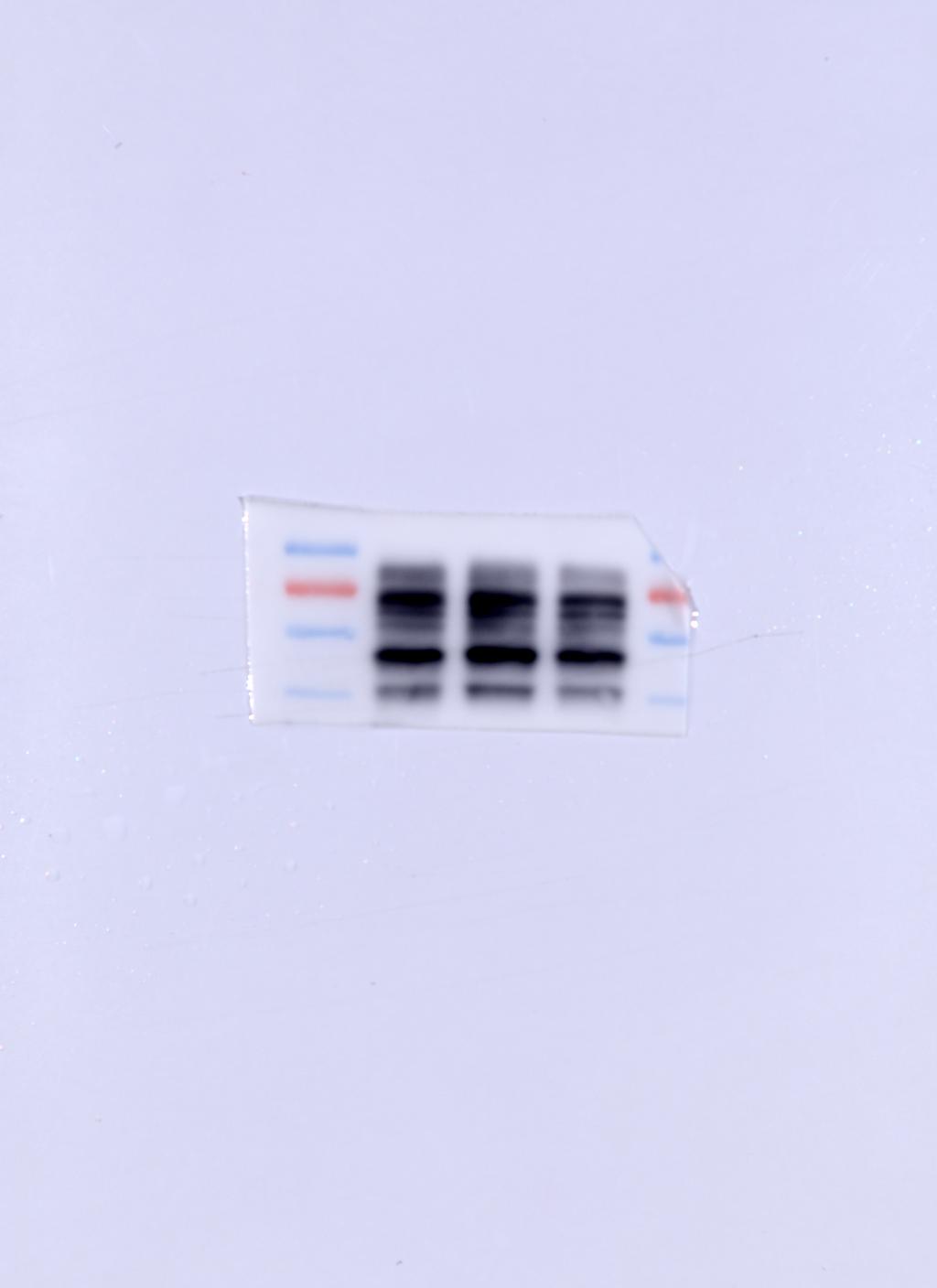

Supplement: Supplemental Information 2 — The CDF files for Fig. 2 are the data of analyzing the fingerprints of 10 batches of the rhizome of M. dauricum Polysaccharide. The software to open this file is the Similarity Evaluation System for Chromatographic Fingerprint of Traditional Chinese Medicine, and the software installation package is available in the Figure 2 folder. The OPJU file in the Figure 3 folder is the data of PCA analysis, and the open software is Origin, which can be downloaded and used for a fee from the Origin official website. [file peerj-10-13946-s002.zip › Raw date/Fig.6/p-JNK/3/JNK+Marker.jpg]

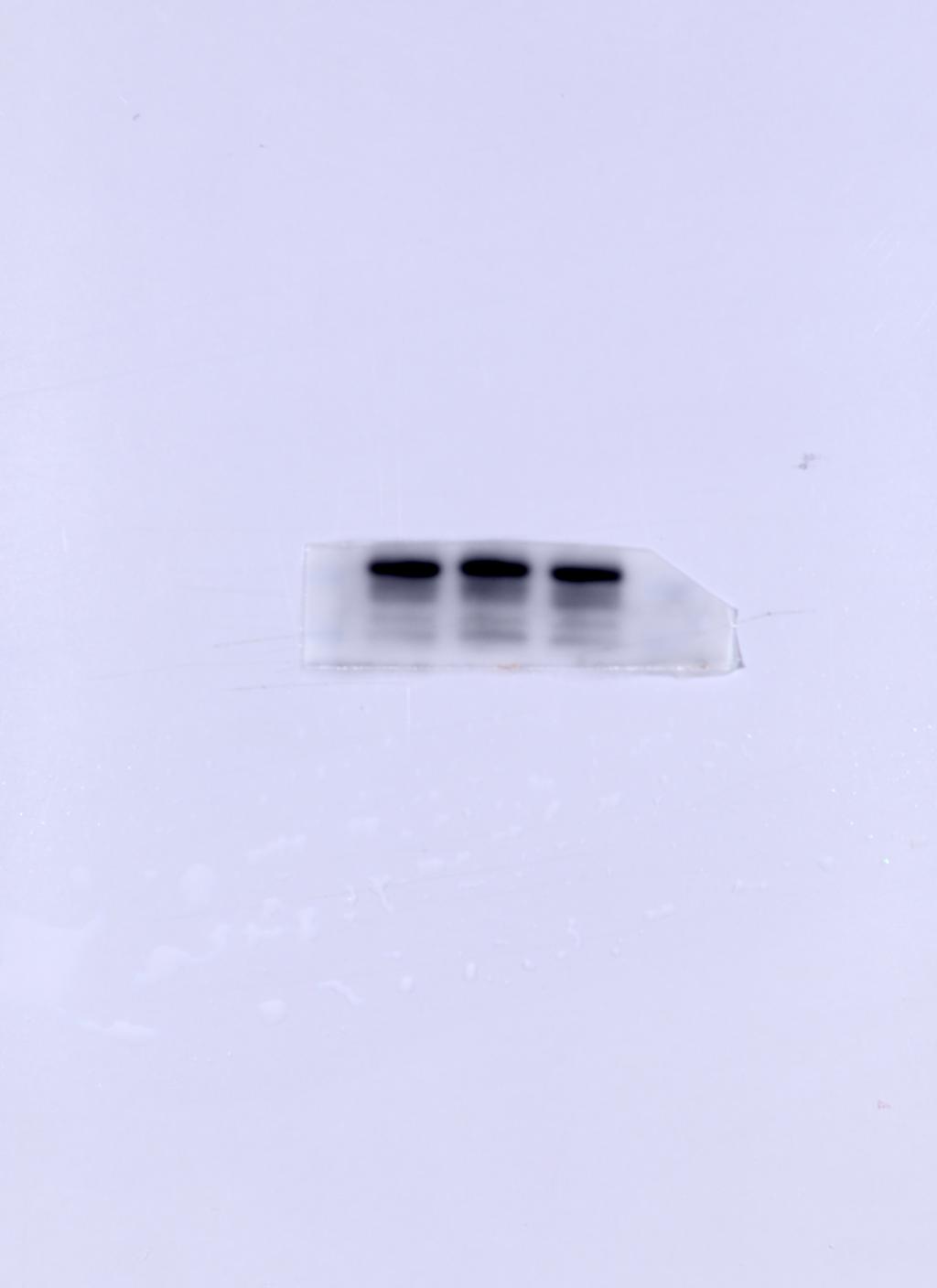

Supplement: Supplemental Information 2 — The CDF files for Fig. 2 are the data of analyzing the fingerprints of 10 batches of the rhizome of M. dauricum Polysaccharide. The software to open this file is the Similarity Evaluation System for Chromatographic Fingerprint of Traditional Chinese Medicine, and the software installation package is available in the Figure 2 folder. The OPJU file in the Figure 3 folder is the data of PCA analysis, and the open software is Origin, which can be downloaded and used for a fee from the Origin official website. [file peerj-10-13946-s002.zip › Raw date/Fig.6/p-JNK/3/JNK-GAPDH+Marker.jpg]

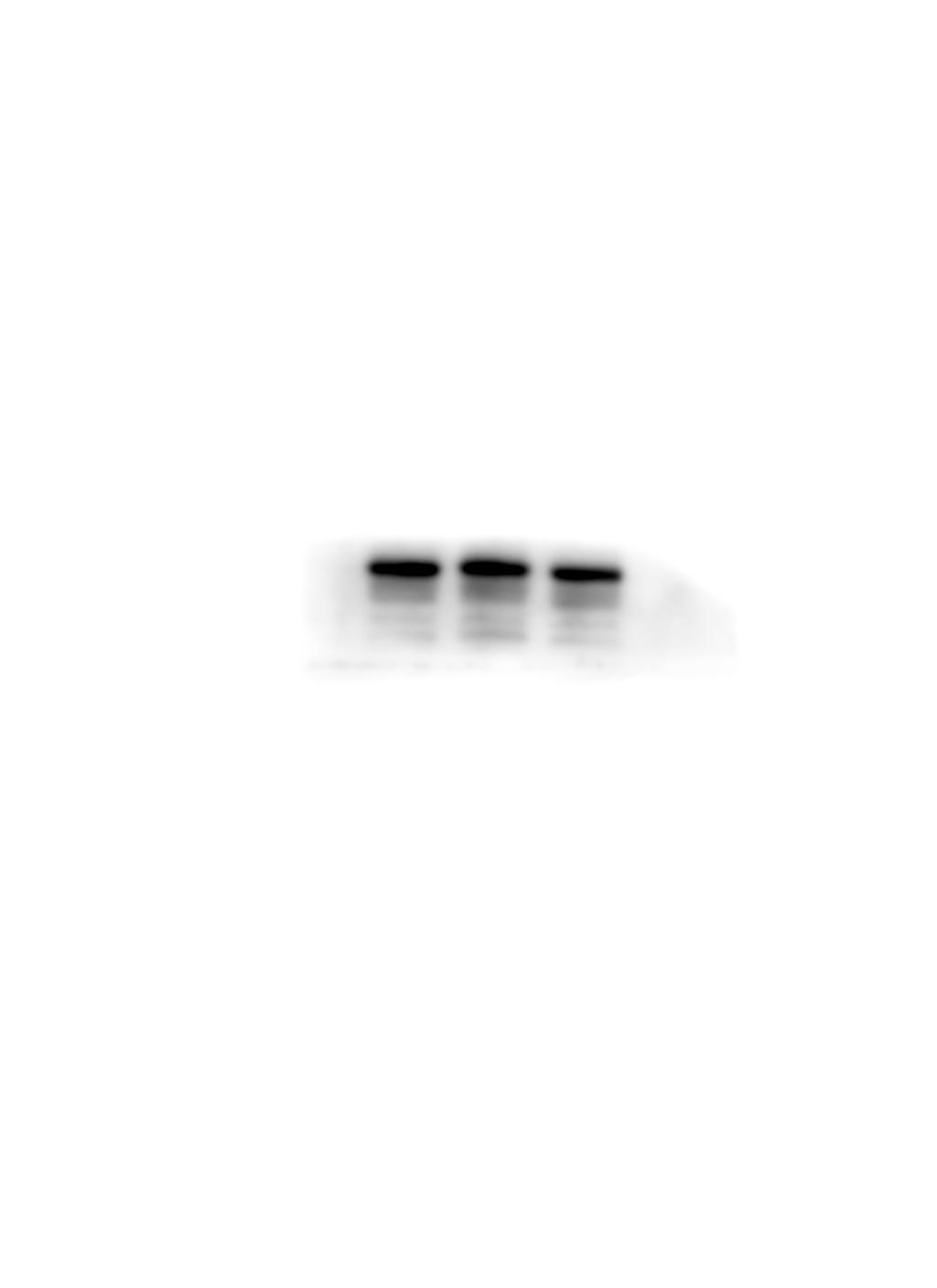

Supplement: Supplemental Information 2 — The CDF files for Fig. 2 are the data of analyzing the fingerprints of 10 batches of the rhizome of M. dauricum Polysaccharide. The software to open this file is the Similarity Evaluation System for Chromatographic Fingerprint of Traditional Chinese Medicine, and the software installation package is available in the Figure 2 folder. The OPJU file in the Figure 3 folder is the data of PCA analysis, and the open software is Origin, which can be downloaded and used for a fee from the Origin official website. [file peerj-10-13946-s002.zip › Raw date/Fig.6/p-JNK/3/JNK-GAPDH.jpg]

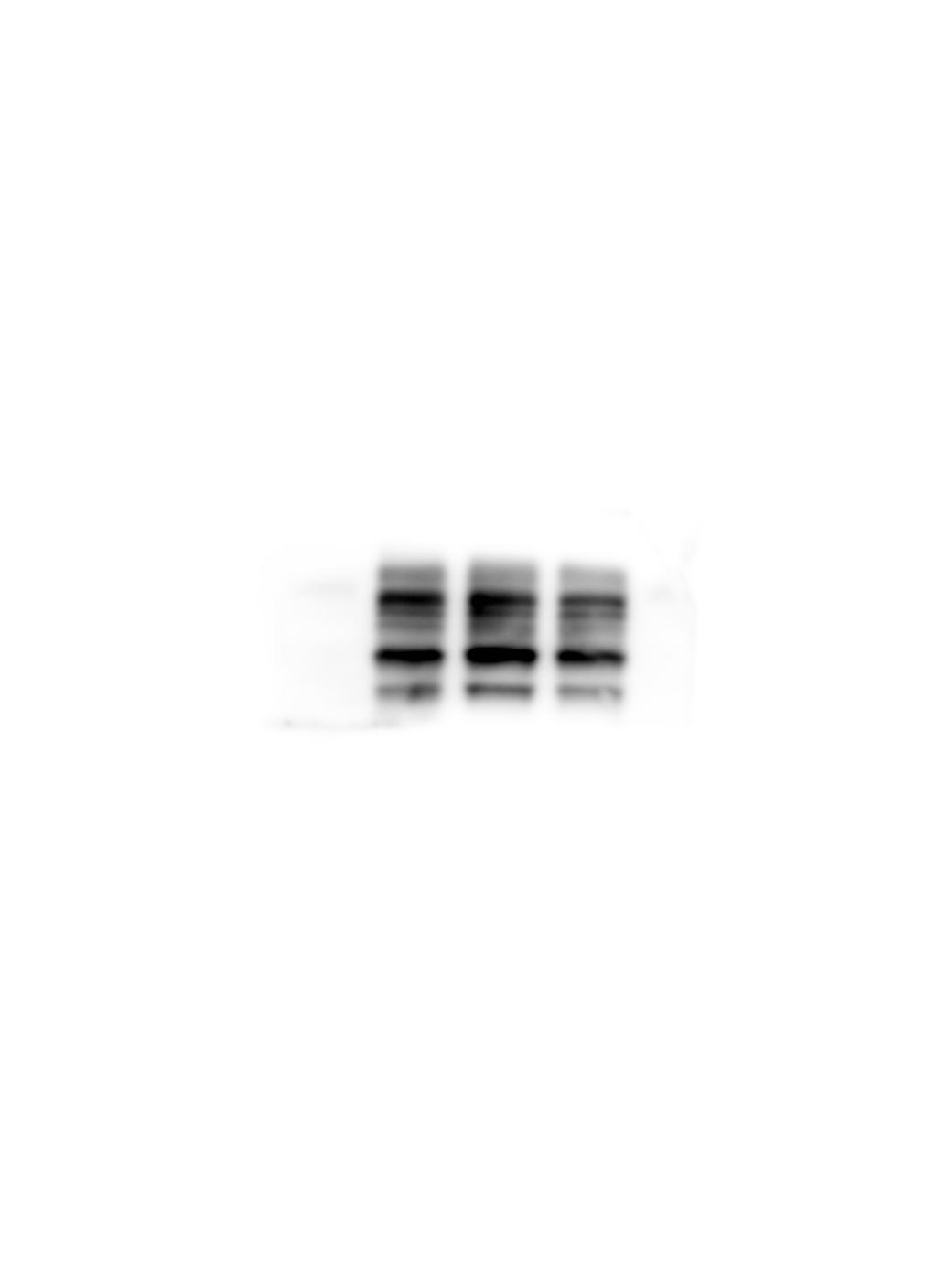

Supplement: Supplemental Information 2 — The CDF files for Fig. 2 are the data of analyzing the fingerprints of 10 batches of the rhizome of M. dauricum Polysaccharide. The software to open this file is the Similarity Evaluation System for Chromatographic Fingerprint of Traditional Chinese Medicine, and the software installation package is available in the Figure 2 folder. The OPJU file in the Figure 3 folder is the data of PCA analysis, and the open software is Origin, which can be downloaded and used for a fee from the Origin official website. [file peerj-10-13946-s002.zip › Raw date/Fig.6/p-JNK/3/JNK.jpg]

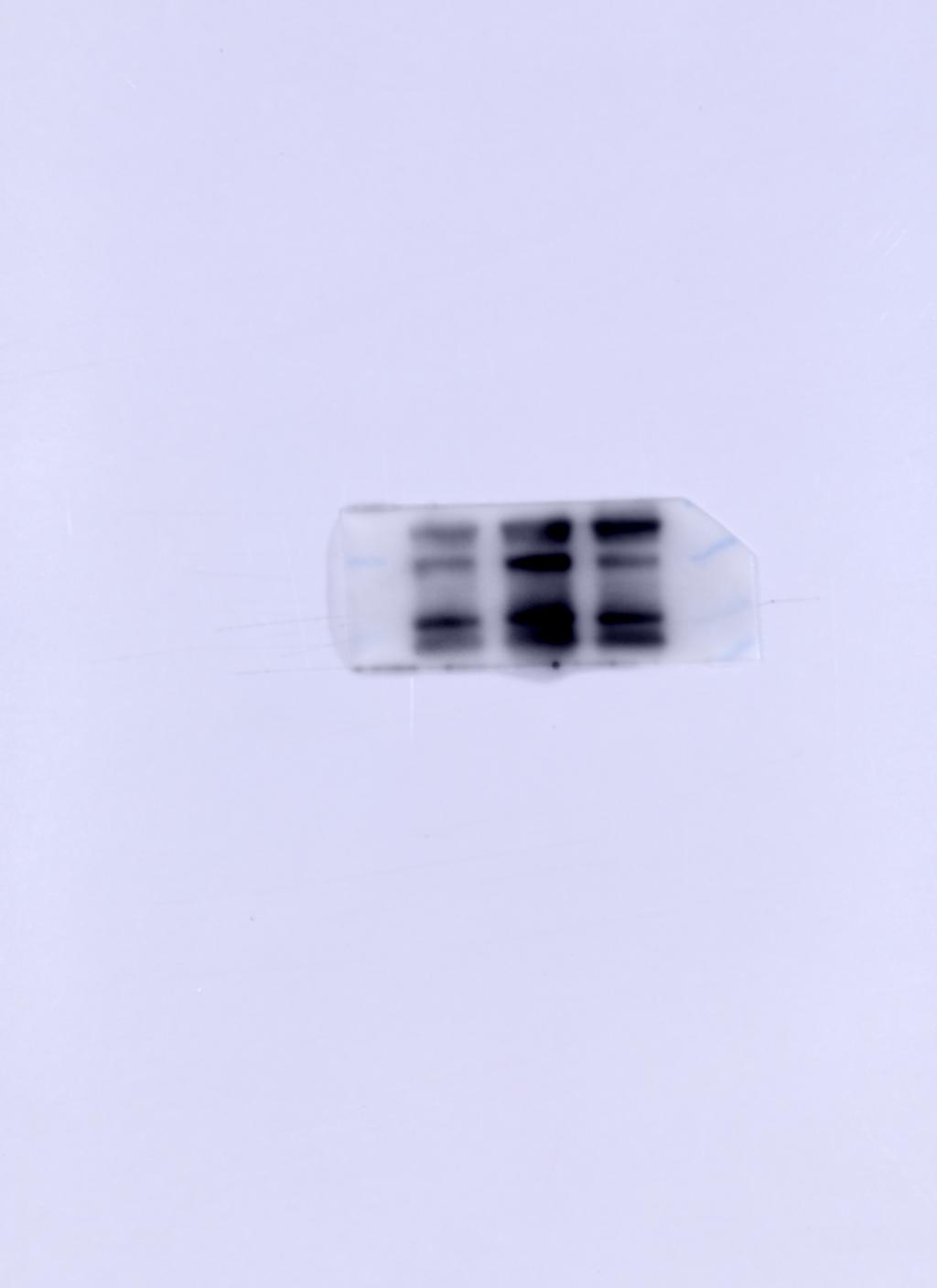

Supplement: Supplemental Information 2 — The CDF files for Fig. 2 are the data of analyzing the fingerprints of 10 batches of the rhizome of M. dauricum Polysaccharide. The software to open this file is the Similarity Evaluation System for Chromatographic Fingerprint of Traditional Chinese Medicine, and the software installation package is available in the Figure 2 folder. The OPJU file in the Figure 3 folder is the data of PCA analysis, and the open software is Origin, which can be downloaded and used for a fee from the Origin official website. [file peerj-10-13946-s002.zip › Raw date/Fig.6/p-JNK/3/p-JNK+Marker.jpg]

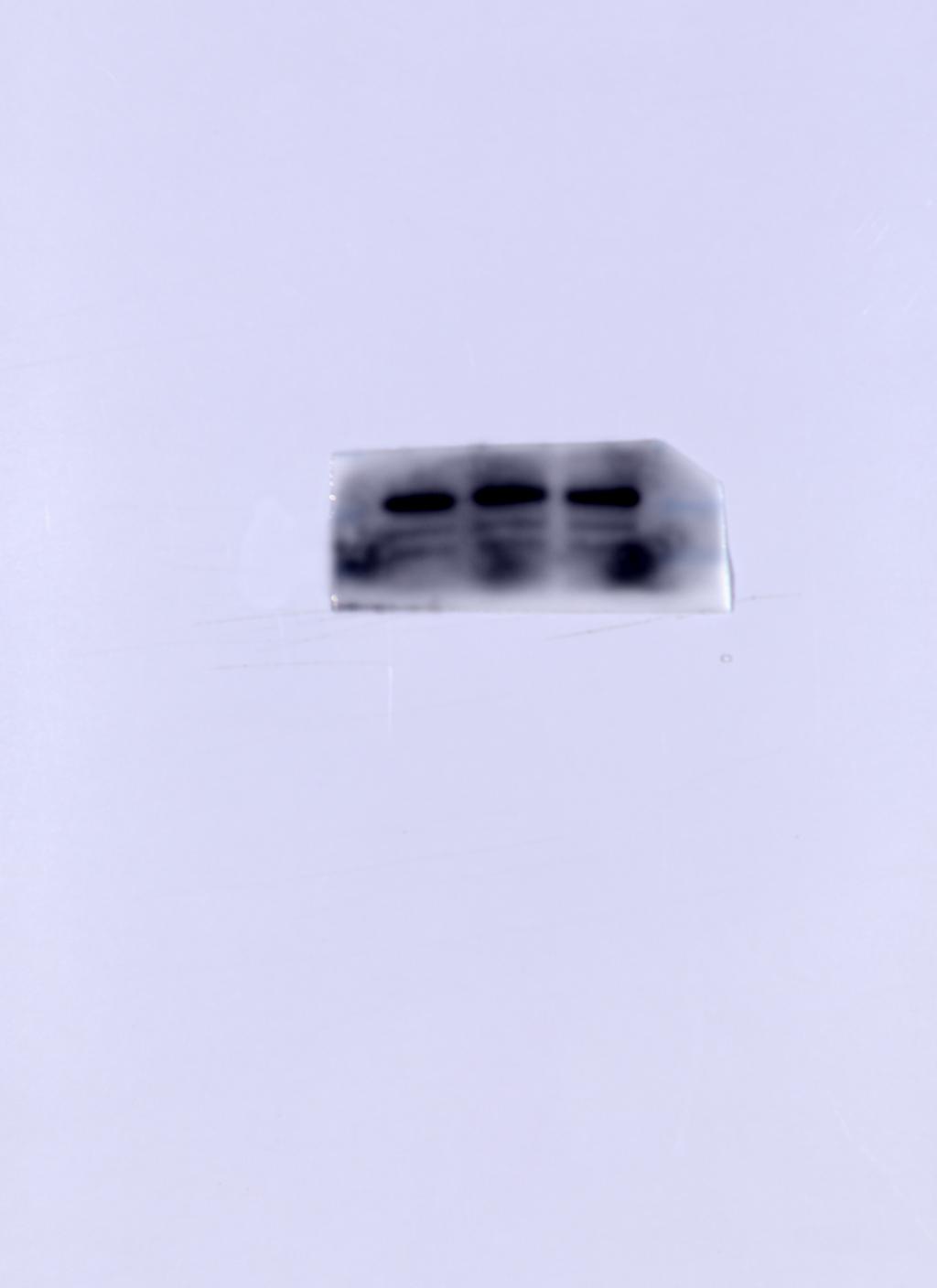

Supplement: Supplemental Information 2 — The CDF files for Fig. 2 are the data of analyzing the fingerprints of 10 batches of the rhizome of M. dauricum Polysaccharide. The software to open this file is the Similarity Evaluation System for Chromatographic Fingerprint of Traditional Chinese Medicine, and the software installation package is available in the Figure 2 folder. The OPJU file in the Figure 3 folder is the data of PCA analysis, and the open software is Origin, which can be downloaded and used for a fee from the Origin official website. [file peerj-10-13946-s002.zip › Raw date/Fig.6/p-JNK/3/p-JNK-GAPDH+Marker.jpg]

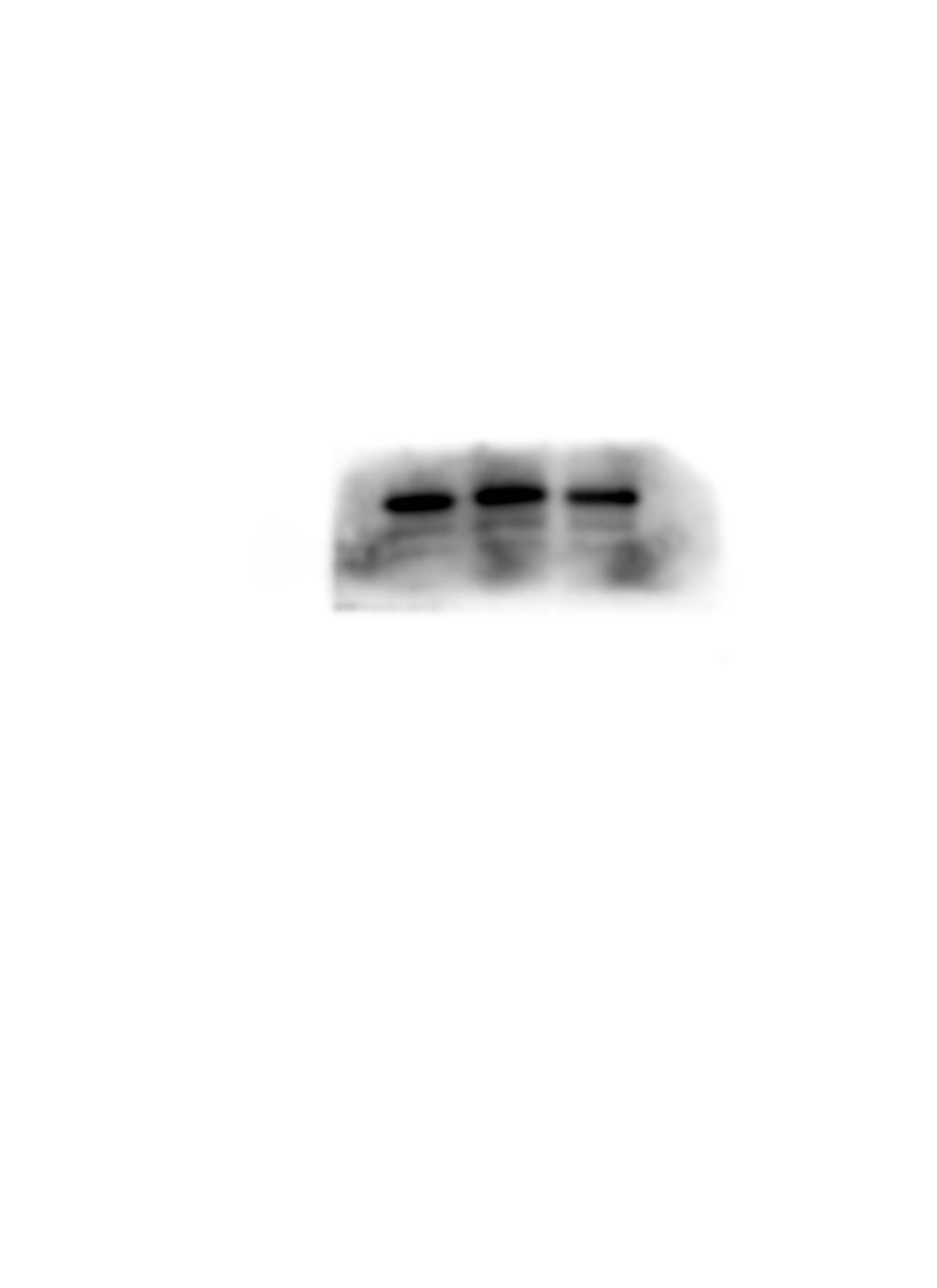

Supplement: Supplemental Information 2 — The CDF files for Fig. 2 are the data of analyzing the fingerprints of 10 batches of the rhizome of M. dauricum Polysaccharide. The software to open this file is the Similarity Evaluation System for Chromatographic Fingerprint of Traditional Chinese Medicine, and the software installation package is available in the Figure 2 folder. The OPJU file in the Figure 3 folder is the data of PCA analysis, and the open software is Origin, which can be downloaded and used for a fee from the Origin official website. [file peerj-10-13946-s002.zip › Raw date/Fig.6/p-JNK/3/p-JNK-GAPDH.jpg]

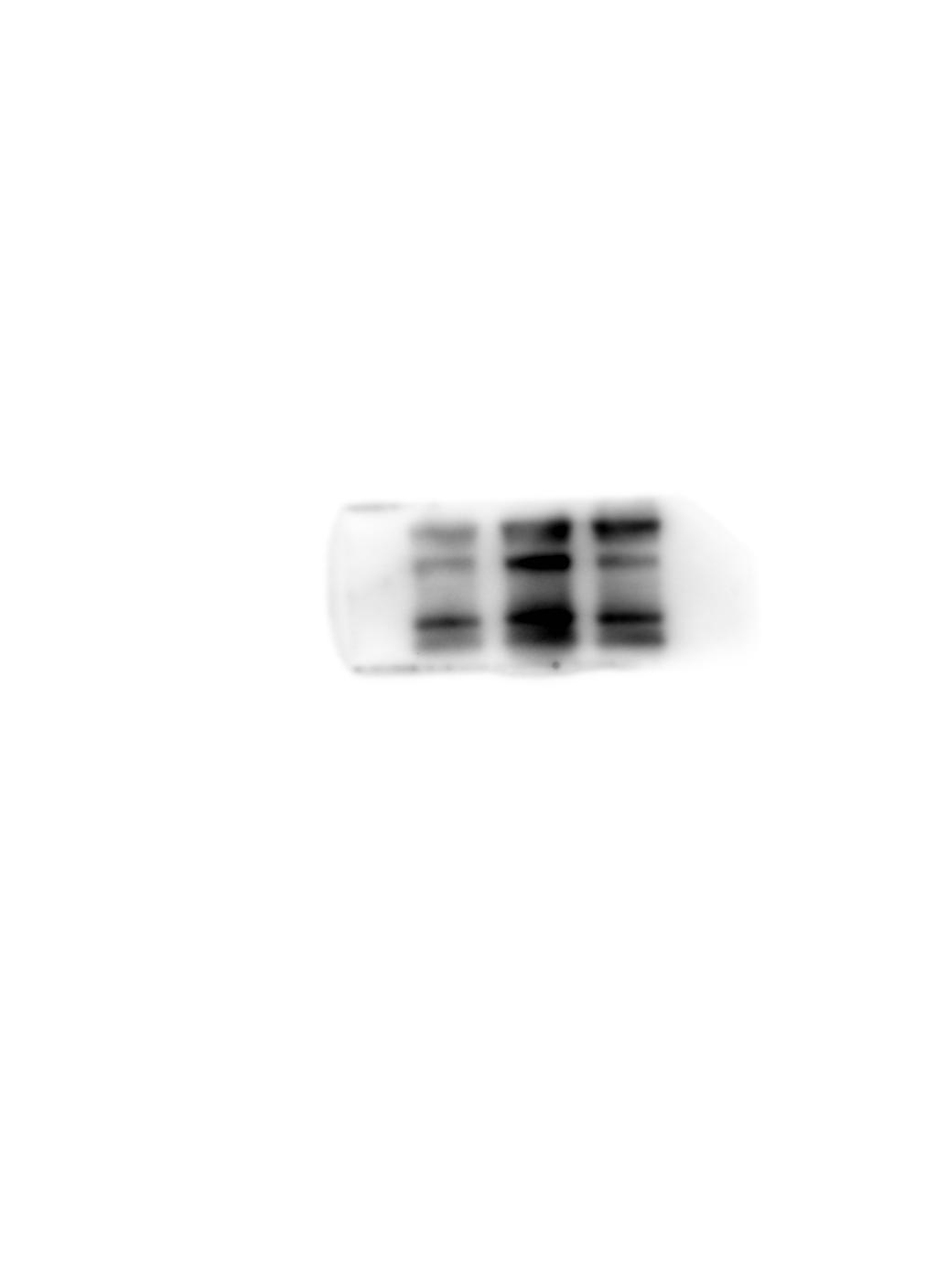

Supplement: Supplemental Information 2 — The CDF files for Fig. 2 are the data of analyzing the fingerprints of 10 batches of the rhizome of M. dauricum Polysaccharide. The software to open this file is the Similarity Evaluation System for Chromatographic Fingerprint of Traditional Chinese Medicine, and the software installation package is available in the Figure 2 folder. The OPJU file in the Figure 3 folder is the data of PCA analysis, and the open software is Origin, which can be downloaded and used for a fee from the Origin official website. [file peerj-10-13946-s002.zip › Raw date/Fig.6/p-JNK/3/p-JNK.jpg]

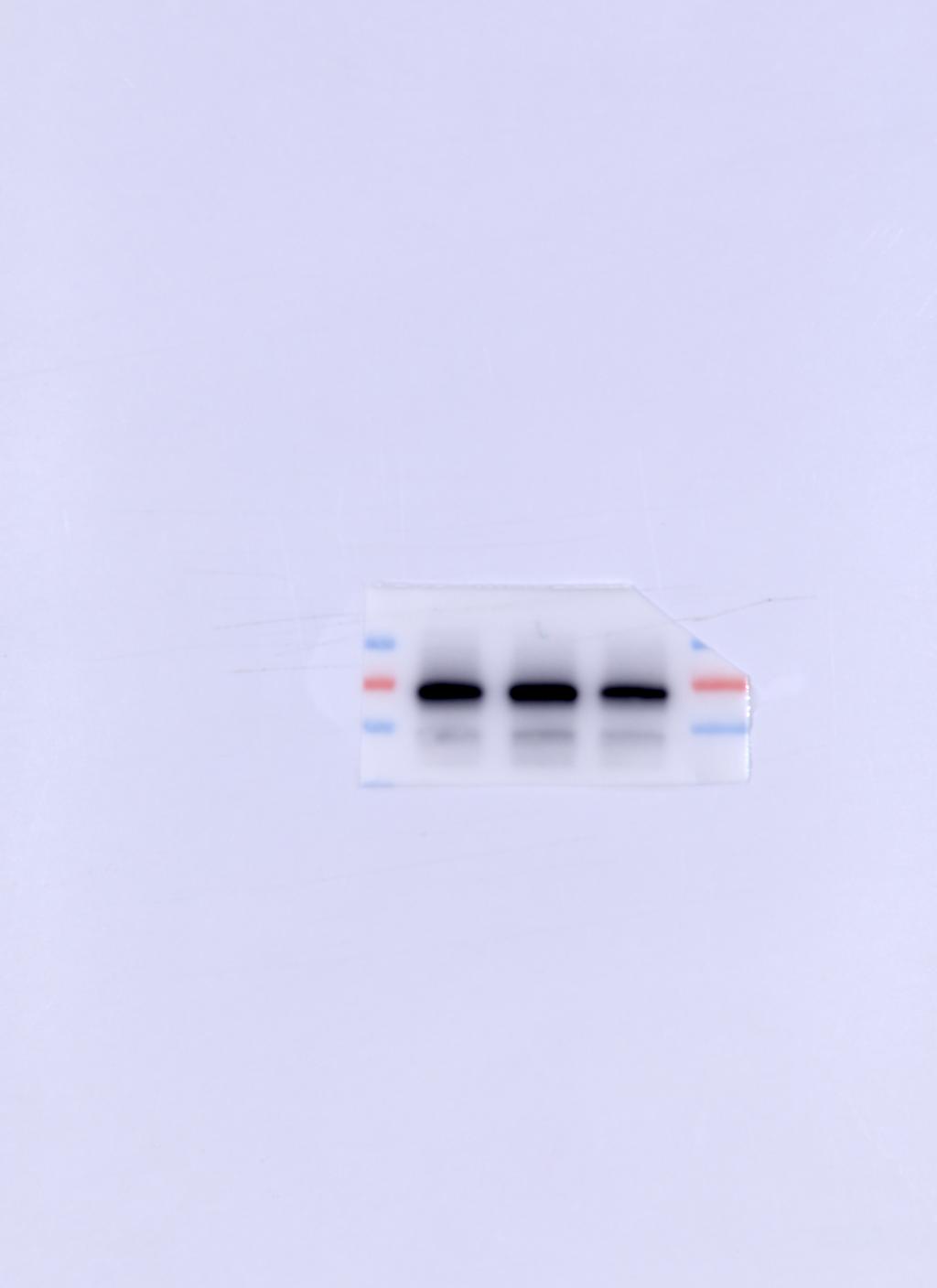

Supplement: Supplemental Information 2 — The CDF files for Fig. 2 are the data of analyzing the fingerprints of 10 batches of the rhizome of M. dauricum Polysaccharide. The software to open this file is the Similarity Evaluation System for Chromatographic Fingerprint of Traditional Chinese Medicine, and the software installation package is available in the Figure 2 folder. The OPJU file in the Figure 3 folder is the data of PCA analysis, and the open software is Origin, which can be downloaded and used for a fee from the Origin official website. [file peerj-10-13946-s002.zip › Raw date/Fig.6/p-NFKB/1/NFκB+Marker.jpg]

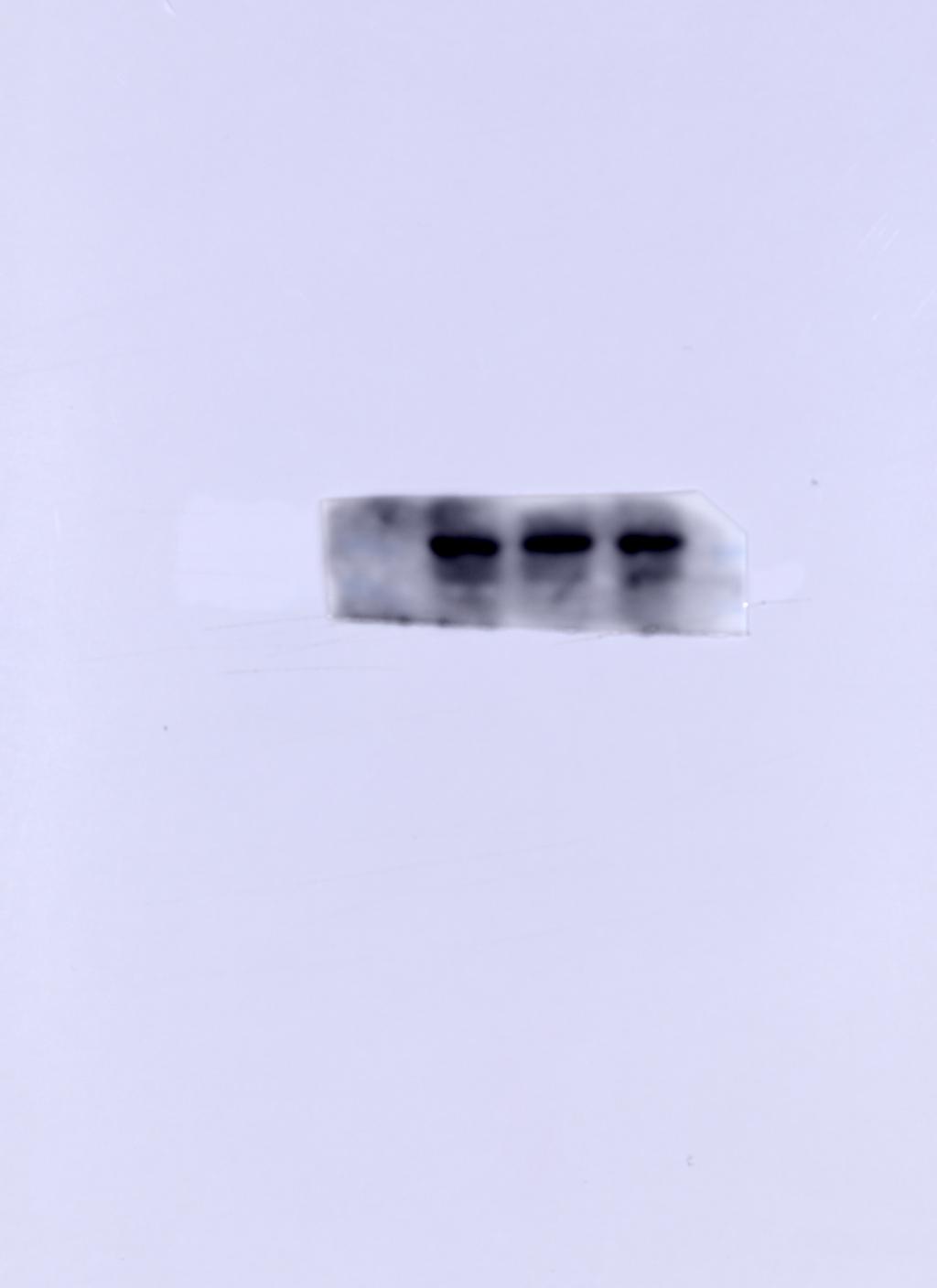

Supplement: Supplemental Information 2 — The CDF files for Fig. 2 are the data of analyzing the fingerprints of 10 batches of the rhizome of M. dauricum Polysaccharide. The software to open this file is the Similarity Evaluation System for Chromatographic Fingerprint of Traditional Chinese Medicine, and the software installation package is available in the Figure 2 folder. The OPJU file in the Figure 3 folder is the data of PCA analysis, and the open software is Origin, which can be downloaded and used for a fee from the Origin official website. [file peerj-10-13946-s002.zip › Raw date/Fig.6/p-NFKB/1/NFκB-GAPDH+Marker.jpg]

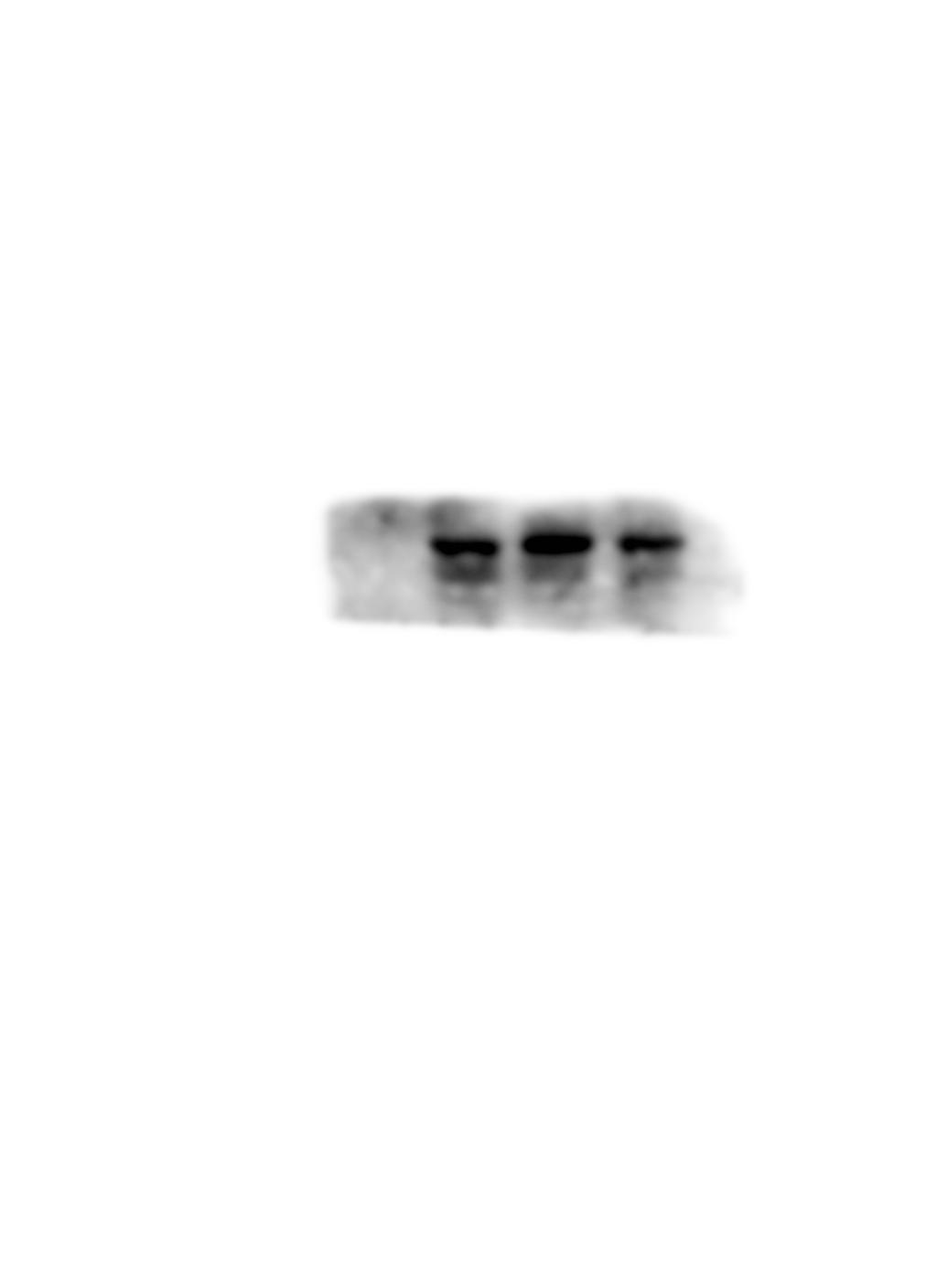

Supplement: Supplemental Information 2 — The CDF files for Fig. 2 are the data of analyzing the fingerprints of 10 batches of the rhizome of M. dauricum Polysaccharide. The software to open this file is the Similarity Evaluation System for Chromatographic Fingerprint of Traditional Chinese Medicine, and the software installation package is available in the Figure 2 folder. The OPJU file in the Figure 3 folder is the data of PCA analysis, and the open software is Origin, which can be downloaded and used for a fee from the Origin official website. [file peerj-10-13946-s002.zip › Raw date/Fig.6/p-NFKB/1/NFκB-GAPDH.jpg]

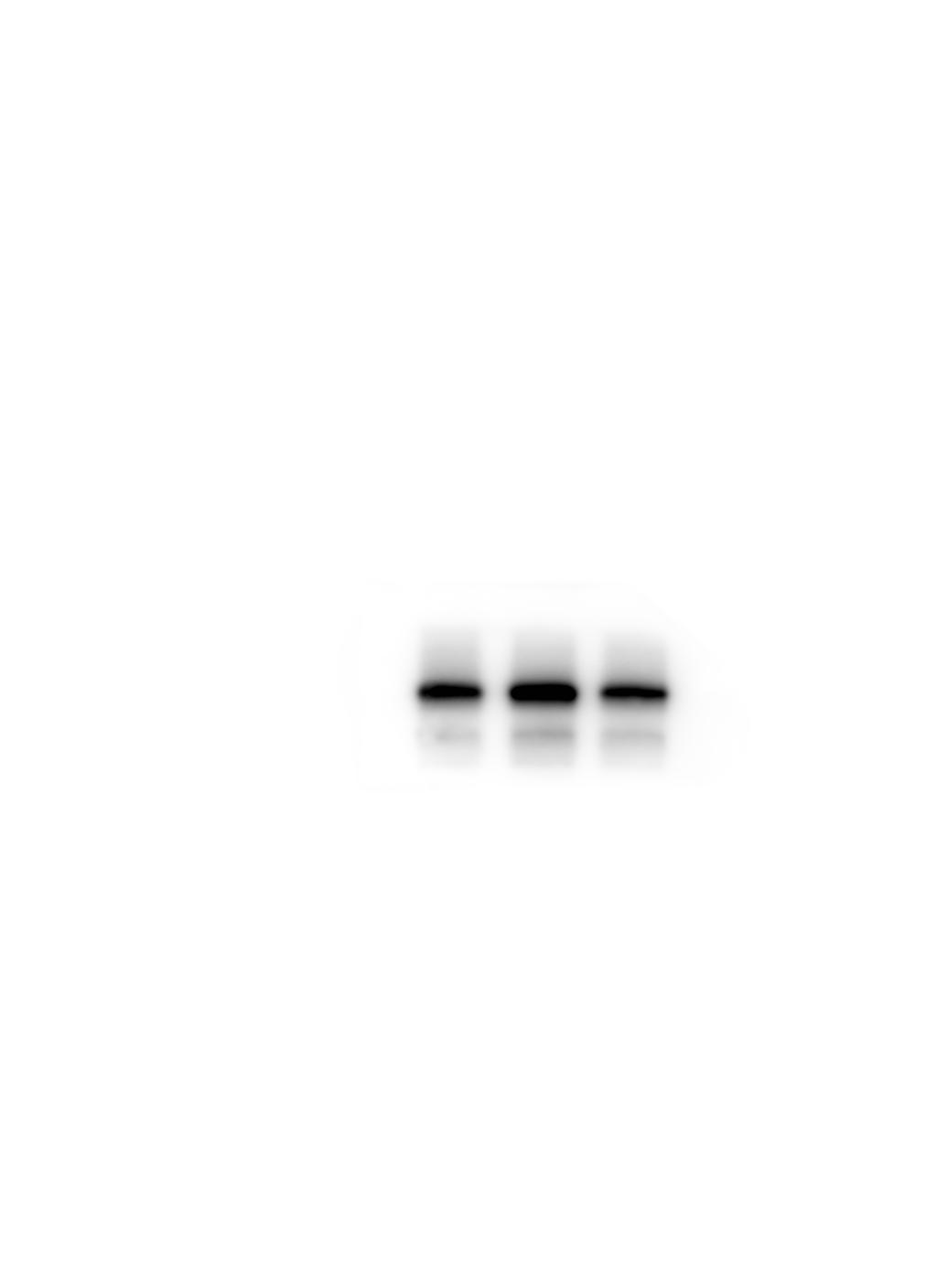

Supplement: Supplemental Information 2 — The CDF files for Fig. 2 are the data of analyzing the fingerprints of 10 batches of the rhizome of M. dauricum Polysaccharide. The software to open this file is the Similarity Evaluation System for Chromatographic Fingerprint of Traditional Chinese Medicine, and the software installation package is available in the Figure 2 folder. The OPJU file in the Figure 3 folder is the data of PCA analysis, and the open software is Origin, which can be downloaded and used for a fee from the Origin official website. [file peerj-10-13946-s002.zip › Raw date/Fig.6/p-NFKB/1/NFκB.jpg]

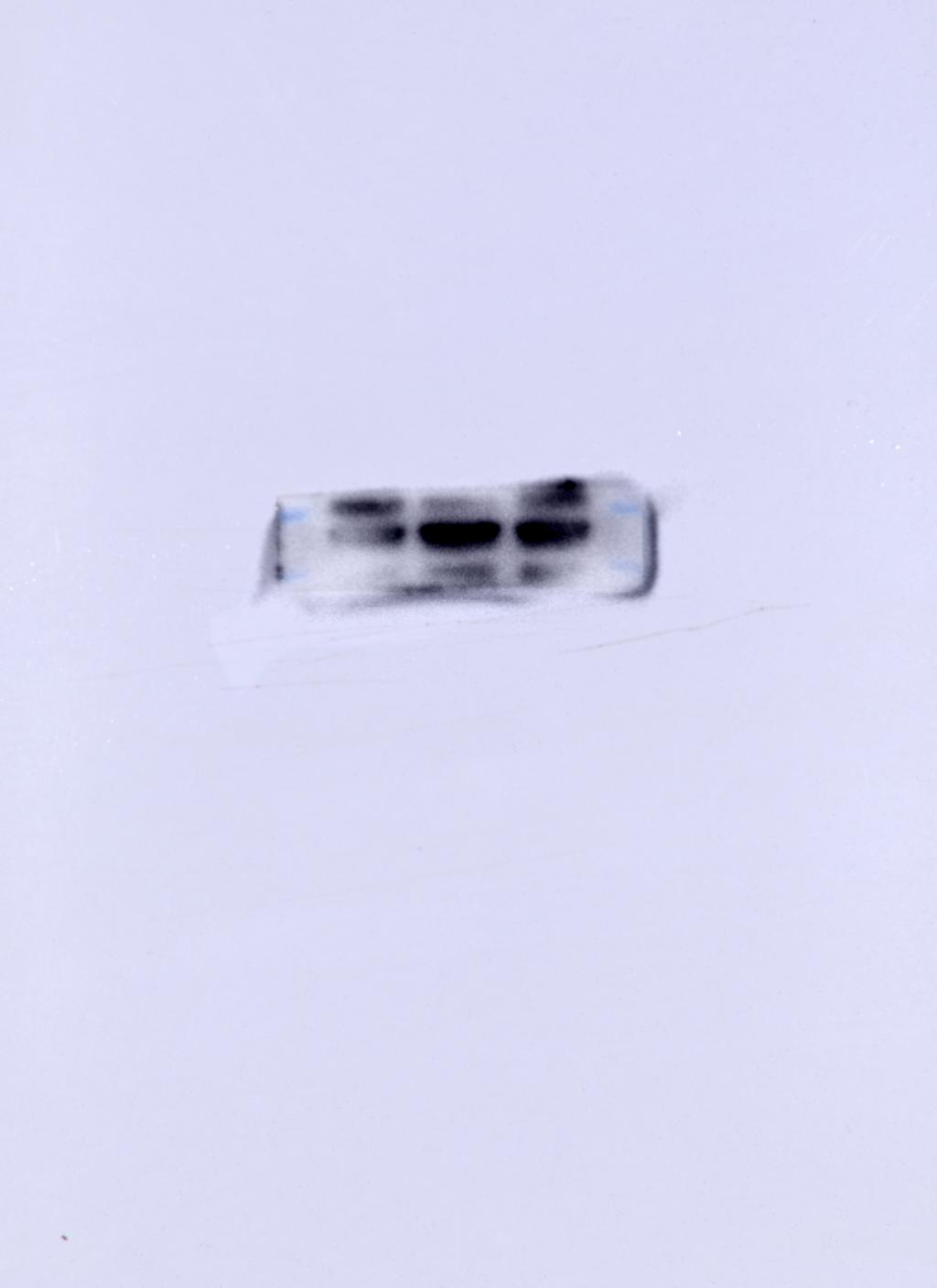

Supplement: Supplemental Information 2 — The CDF files for Fig. 2 are the data of analyzing the fingerprints of 10 batches of the rhizome of M. dauricum Polysaccharide. The software to open this file is the Similarity Evaluation System for Chromatographic Fingerprint of Traditional Chinese Medicine, and the software installation package is available in the Figure 2 folder. The OPJU file in the Figure 3 folder is the data of PCA analysis, and the open software is Origin, which can be downloaded and used for a fee from the Origin official website. [file peerj-10-13946-s002.zip › Raw date/Fig.6/p-NFKB/1/p-NFκB+Marker.jpg]

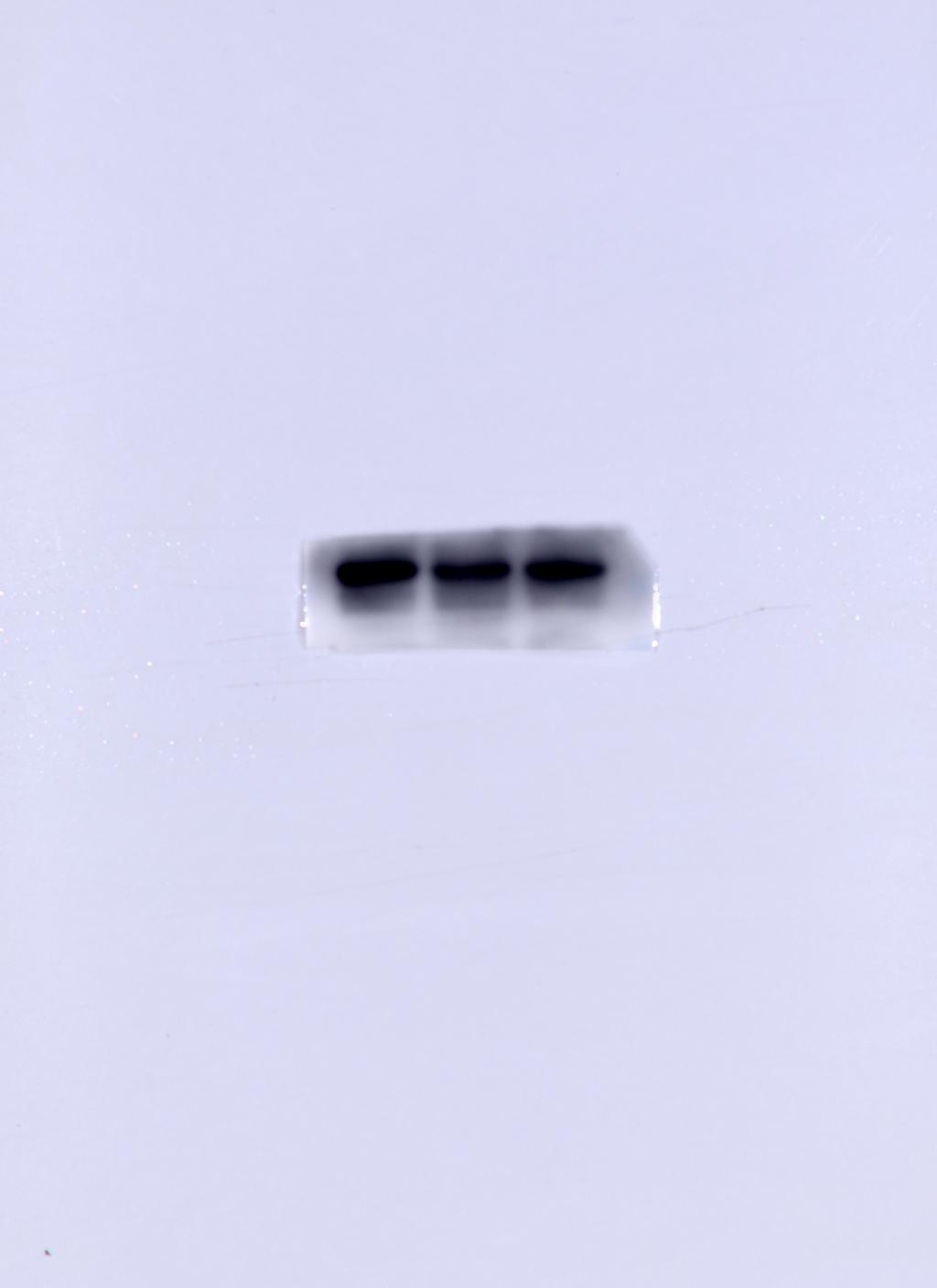

Supplement: Supplemental Information 2 — The CDF files for Fig. 2 are the data of analyzing the fingerprints of 10 batches of the rhizome of M. dauricum Polysaccharide. The software to open this file is the Similarity Evaluation System for Chromatographic Fingerprint of Traditional Chinese Medicine, and the software installation package is available in the Figure 2 folder. The OPJU file in the Figure 3 folder is the data of PCA analysis, and the open software is Origin, which can be downloaded and used for a fee from the Origin official website. [file peerj-10-13946-s002.zip › Raw date/Fig.6/p-NFKB/1/p-NFκB-GAPDH+Marker.jpg]

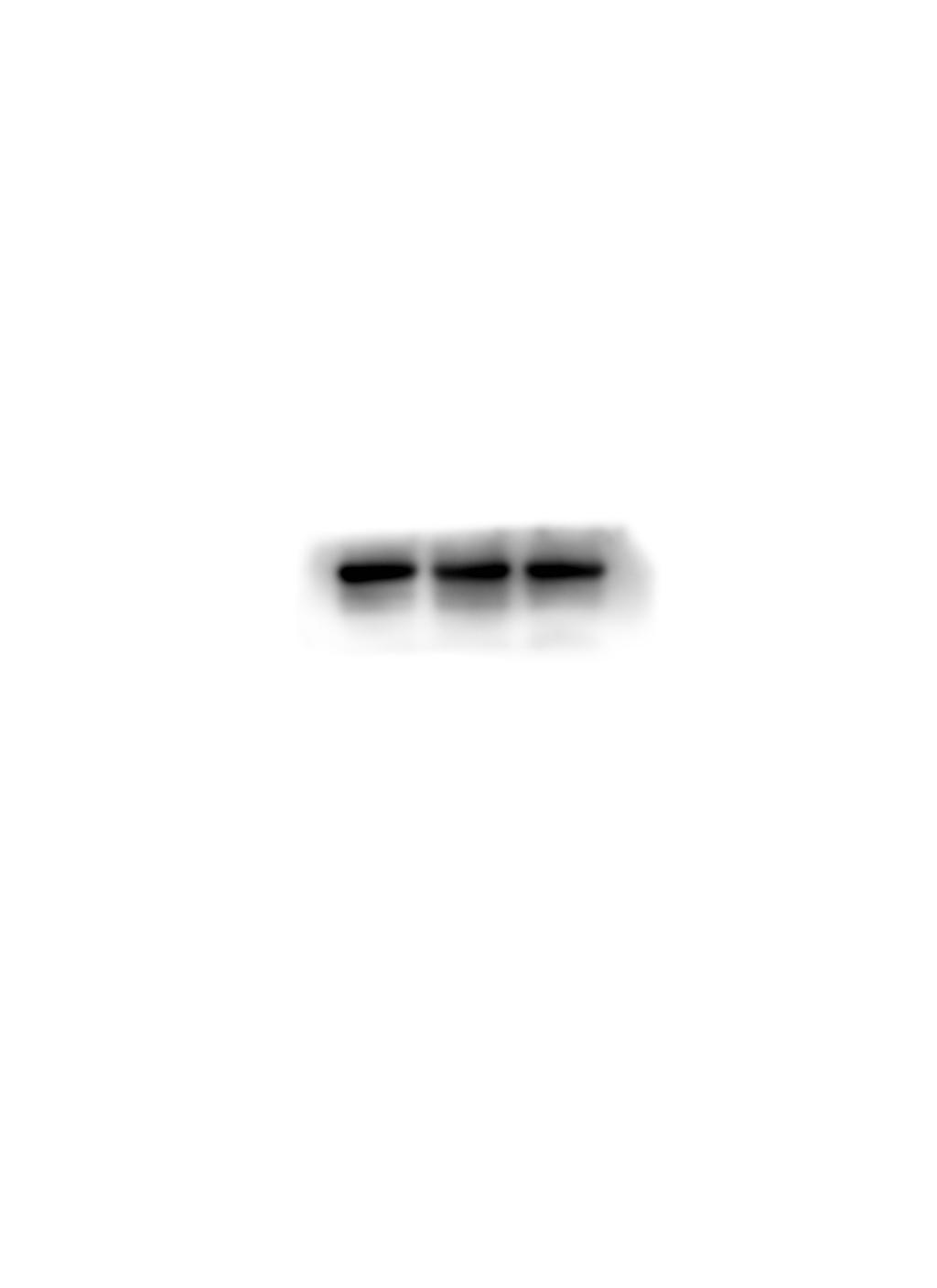

Supplement: Supplemental Information 2 — The CDF files for Fig. 2 are the data of analyzing the fingerprints of 10 batches of the rhizome of M. dauricum Polysaccharide. The software to open this file is the Similarity Evaluation System for Chromatographic Fingerprint of Traditional Chinese Medicine, and the software installation package is available in the Figure 2 folder. The OPJU file in the Figure 3 folder is the data of PCA analysis, and the open software is Origin, which can be downloaded and used for a fee from the Origin official website. [file peerj-10-13946-s002.zip › Raw date/Fig.6/p-NFKB/1/p-NFκB-GAPDH.jpg]

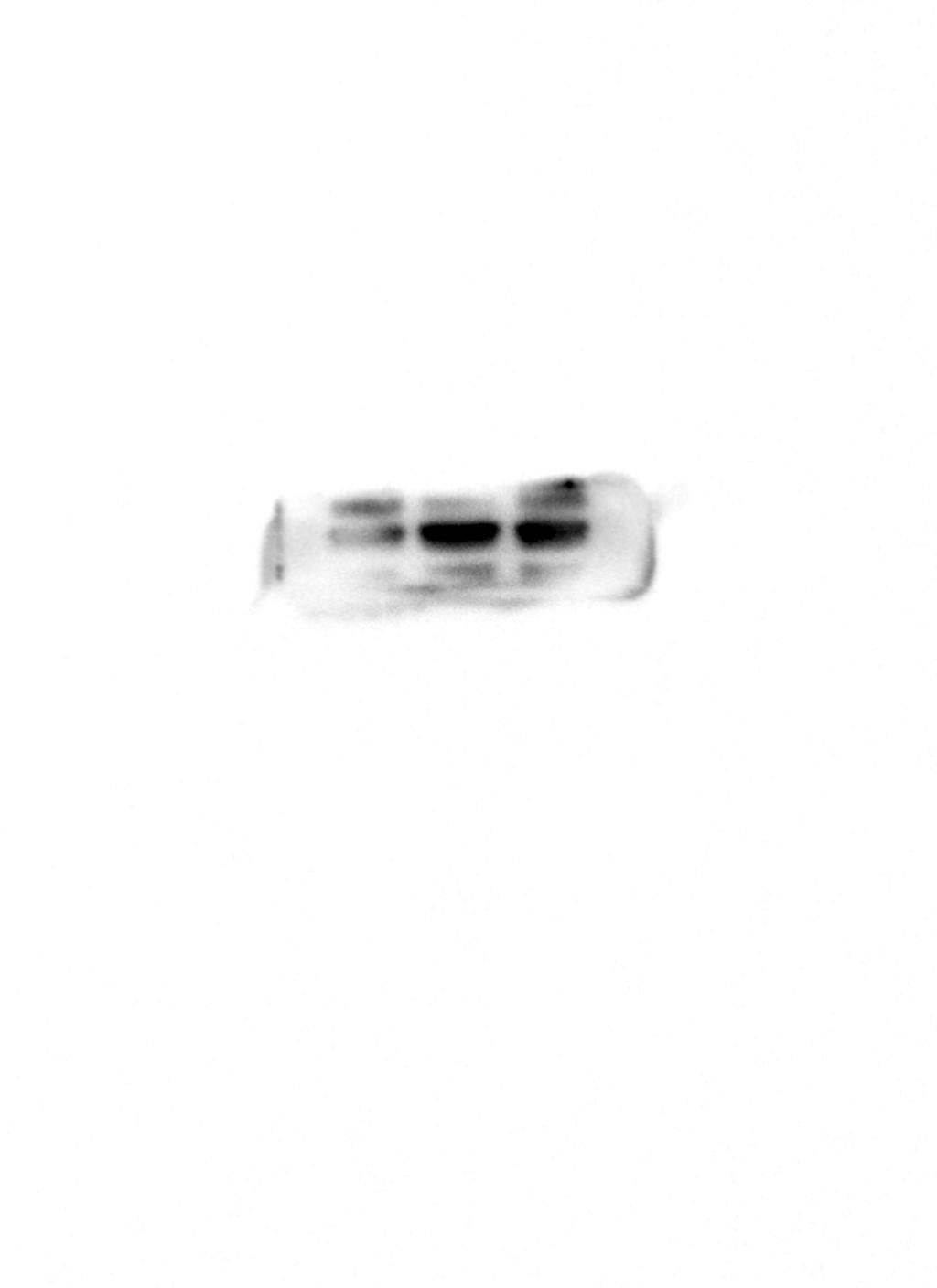

Supplement: Supplemental Information 2 — The CDF files for Fig. 2 are the data of analyzing the fingerprints of 10 batches of the rhizome of M. dauricum Polysaccharide. The software to open this file is the Similarity Evaluation System for Chromatographic Fingerprint of Traditional Chinese Medicine, and the software installation package is available in the Figure 2 folder. The OPJU file in the Figure 3 folder is the data of PCA analysis, and the open software is Origin, which can be downloaded and used for a fee from the Origin official website. [file peerj-10-13946-s002.zip › Raw date/Fig.6/p-NFKB/1/p-NFκB.jpg]

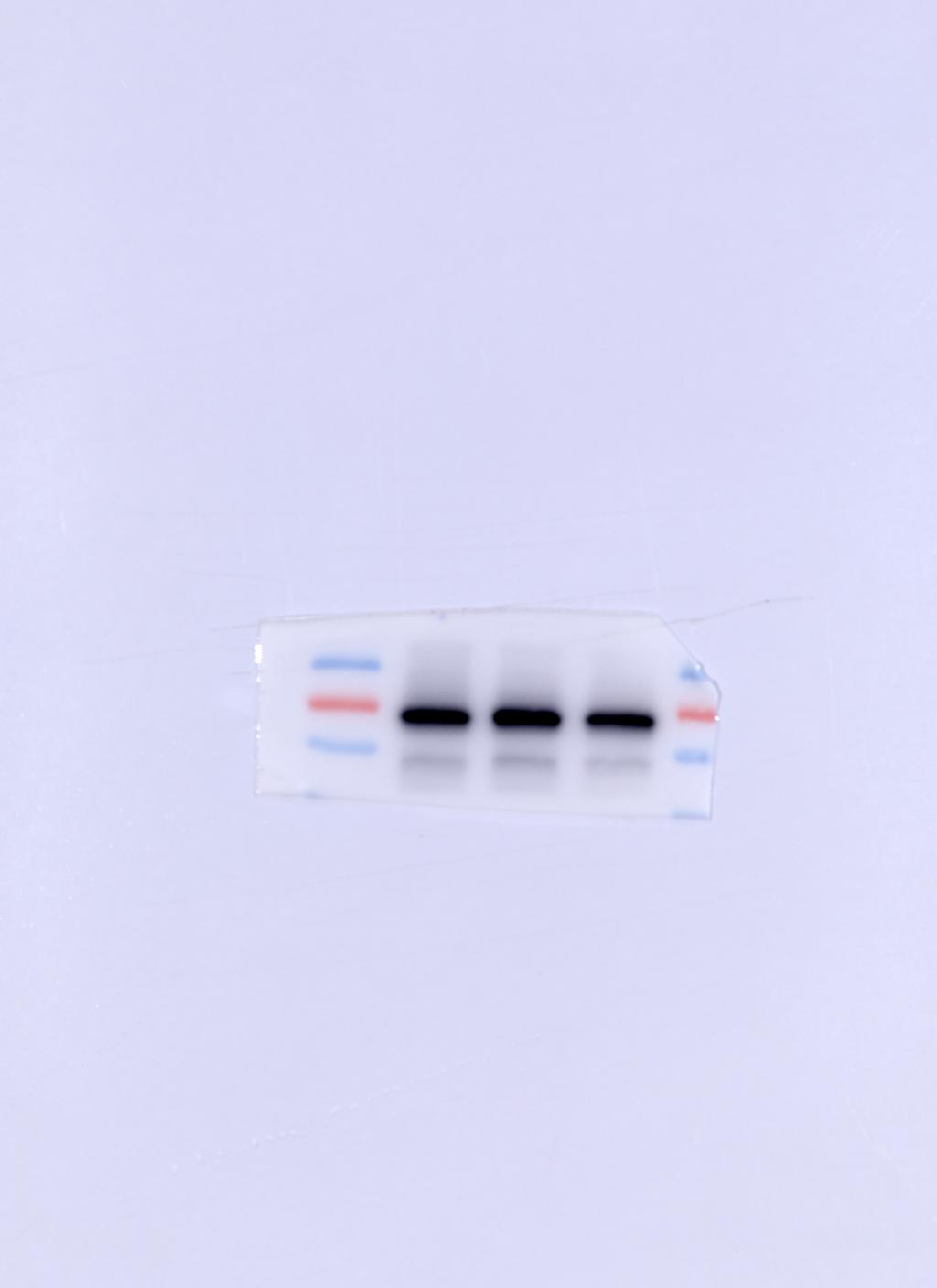

Supplement: Supplemental Information 2 — The CDF files for Fig. 2 are the data of analyzing the fingerprints of 10 batches of the rhizome of M. dauricum Polysaccharide. The software to open this file is the Similarity Evaluation System for Chromatographic Fingerprint of Traditional Chinese Medicine, and the software installation package is available in the Figure 2 folder. The OPJU file in the Figure 3 folder is the data of PCA analysis, and the open software is Origin, which can be downloaded and used for a fee from the Origin official website. [file peerj-10-13946-s002.zip › Raw date/Fig.6/p-NFKB/2/NFκB+Marker.jpg]

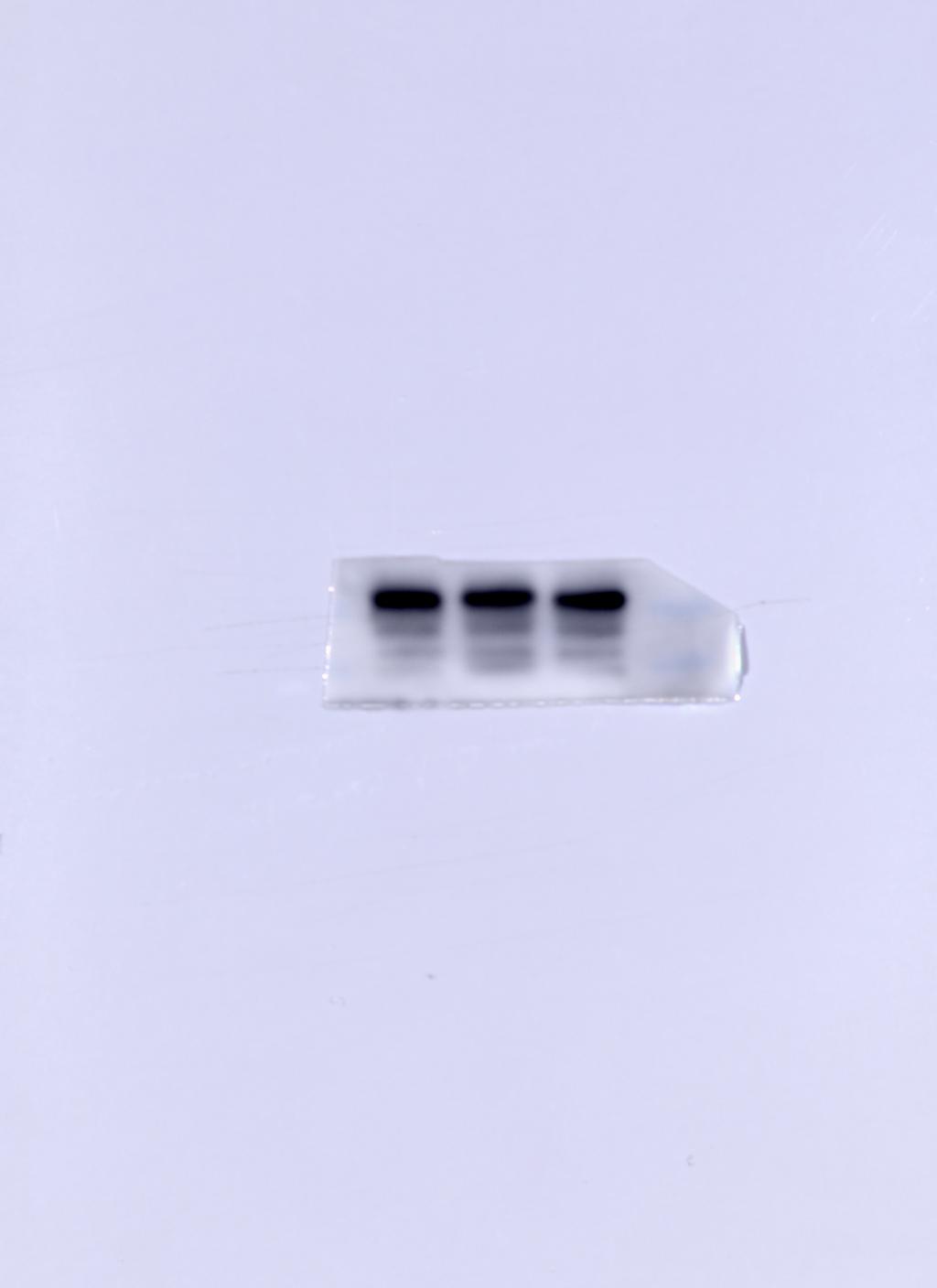

Supplement: Supplemental Information 2 — The CDF files for Fig. 2 are the data of analyzing the fingerprints of 10 batches of the rhizome of M. dauricum Polysaccharide. The software to open this file is the Similarity Evaluation System for Chromatographic Fingerprint of Traditional Chinese Medicine, and the software installation package is available in the Figure 2 folder. The OPJU file in the Figure 3 folder is the data of PCA analysis, and the open software is Origin, which can be downloaded and used for a fee from the Origin official website. [file peerj-10-13946-s002.zip › Raw date/Fig.6/p-NFKB/2/NFκB-GAPDH+Marker.jpg]

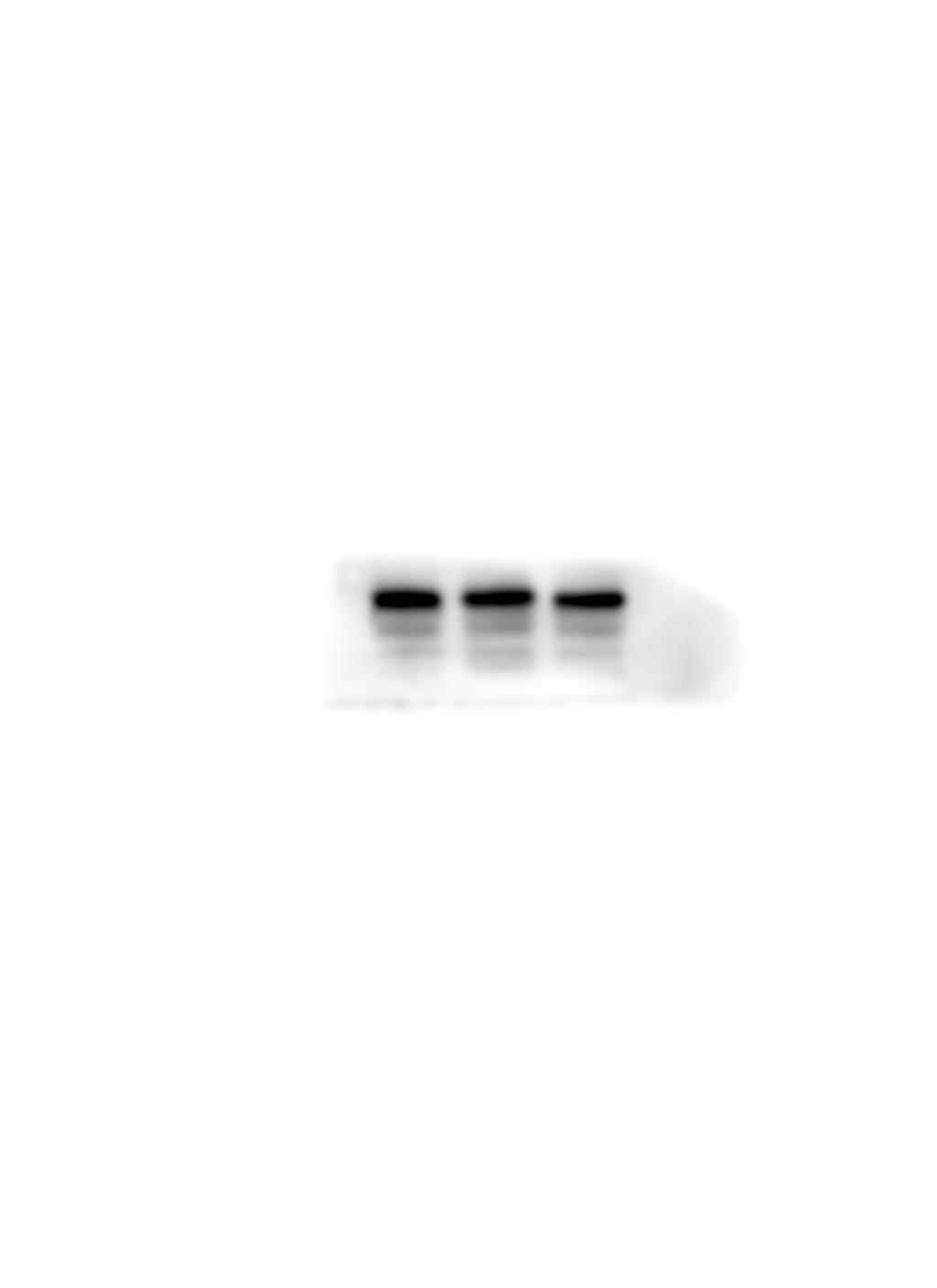

Supplement: Supplemental Information 2 — The CDF files for Fig. 2 are the data of analyzing the fingerprints of 10 batches of the rhizome of M. dauricum Polysaccharide. The software to open this file is the Similarity Evaluation System for Chromatographic Fingerprint of Traditional Chinese Medicine, and the software installation package is available in the Figure 2 folder. The OPJU file in the Figure 3 folder is the data of PCA analysis, and the open software is Origin, which can be downloaded and used for a fee from the Origin official website. [file peerj-10-13946-s002.zip › Raw date/Fig.6/p-NFKB/2/NFκB-GAPDH.jpg]

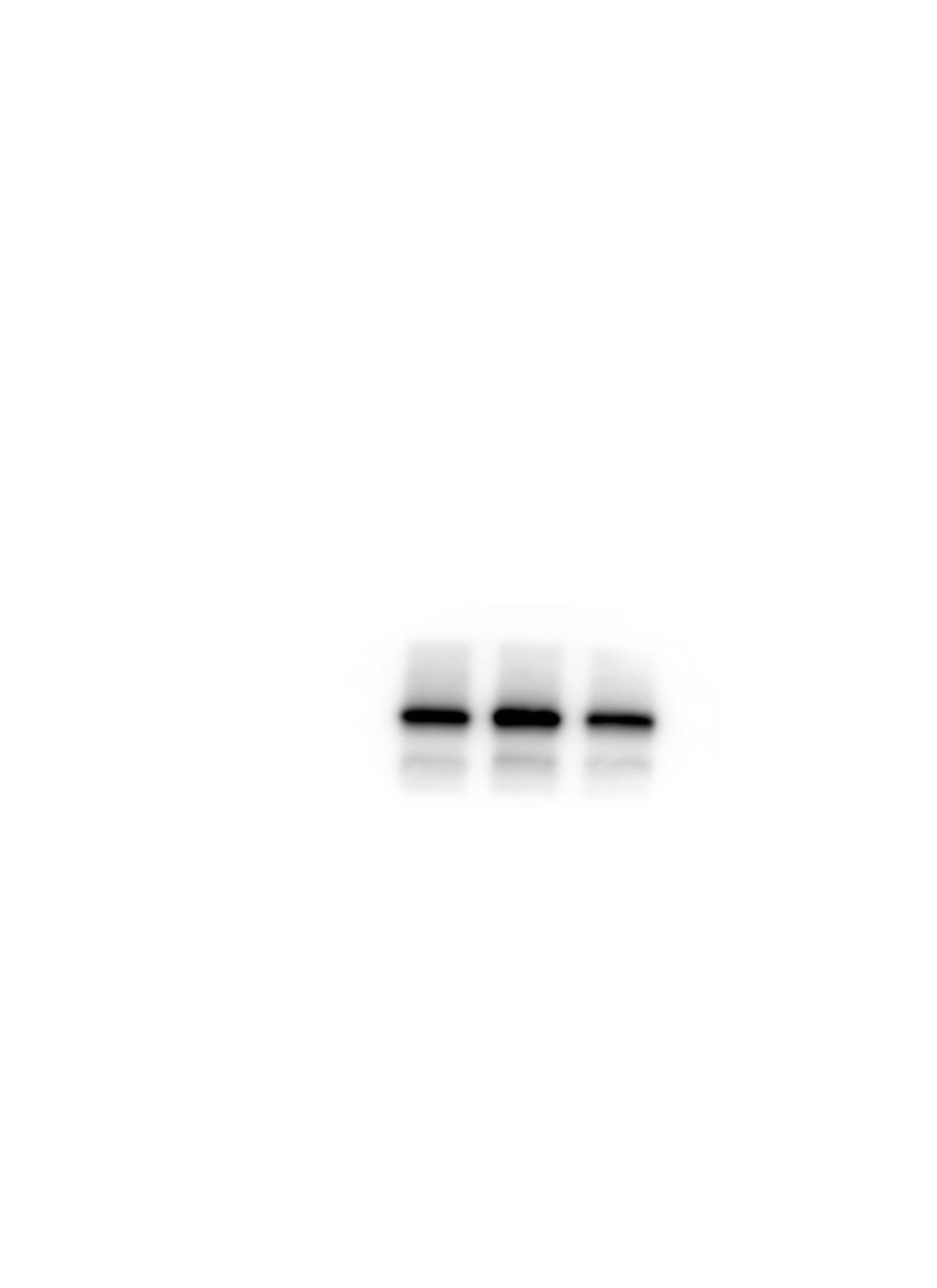

Supplement: Supplemental Information 2 — The CDF files for Fig. 2 are the data of analyzing the fingerprints of 10 batches of the rhizome of M. dauricum Polysaccharide. The software to open this file is the Similarity Evaluation System for Chromatographic Fingerprint of Traditional Chinese Medicine, and the software installation package is available in the Figure 2 folder. The OPJU file in the Figure 3 folder is the data of PCA analysis, and the open software is Origin, which can be downloaded and used for a fee from the Origin official website. [file peerj-10-13946-s002.zip › Raw date/Fig.6/p-NFKB/2/NFκB.jpg]

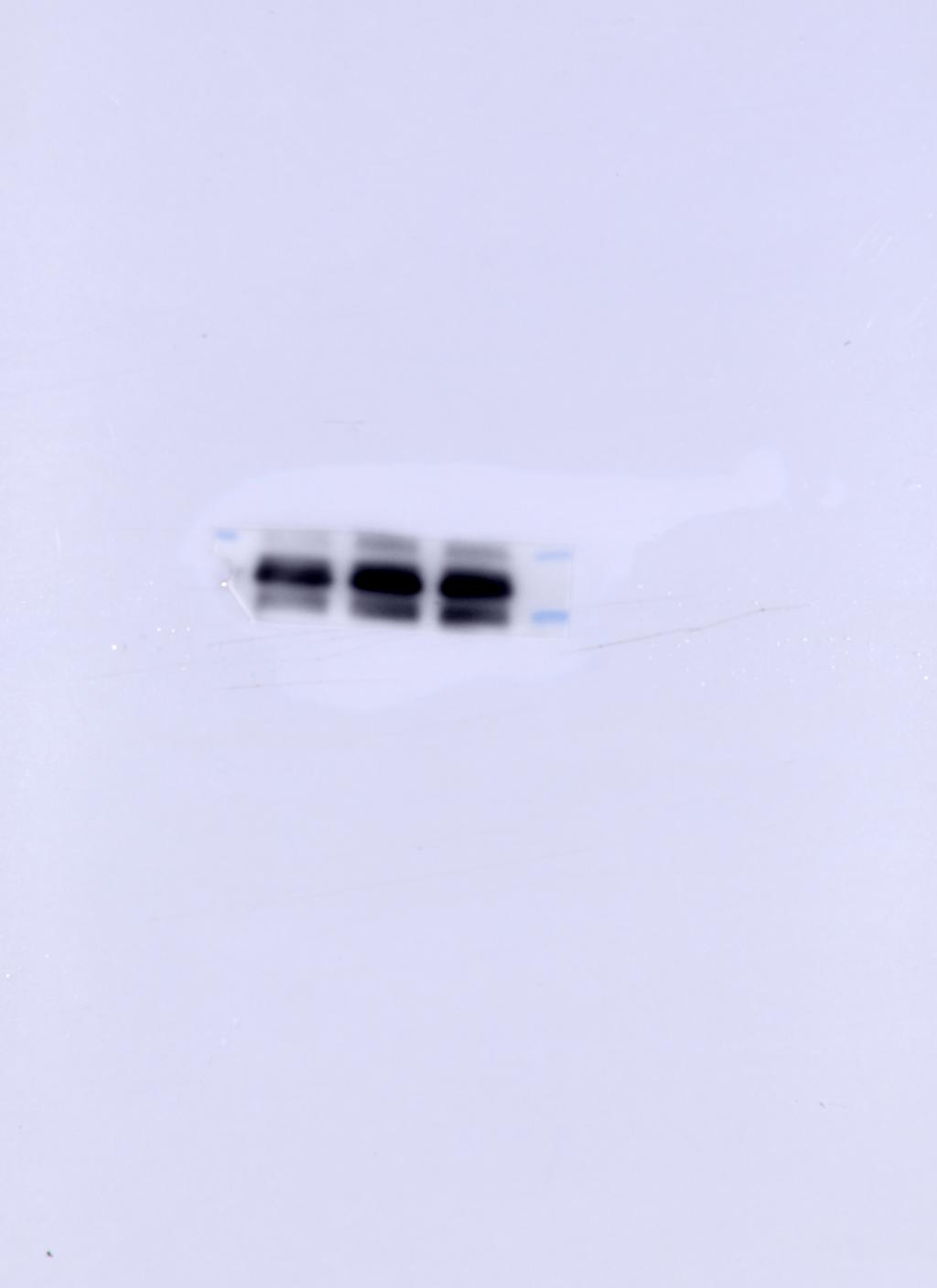

Supplement: Supplemental Information 2 — The CDF files for Fig. 2 are the data of analyzing the fingerprints of 10 batches of the rhizome of M. dauricum Polysaccharide. The software to open this file is the Similarity Evaluation System for Chromatographic Fingerprint of Traditional Chinese Medicine, and the software installation package is available in the Figure 2 folder. The OPJU file in the Figure 3 folder is the data of PCA analysis, and the open software is Origin, which can be downloaded and used for a fee from the Origin official website. [file peerj-10-13946-s002.zip › Raw date/Fig.6/p-NFKB/2/p-NFκB+Marker.jpg]

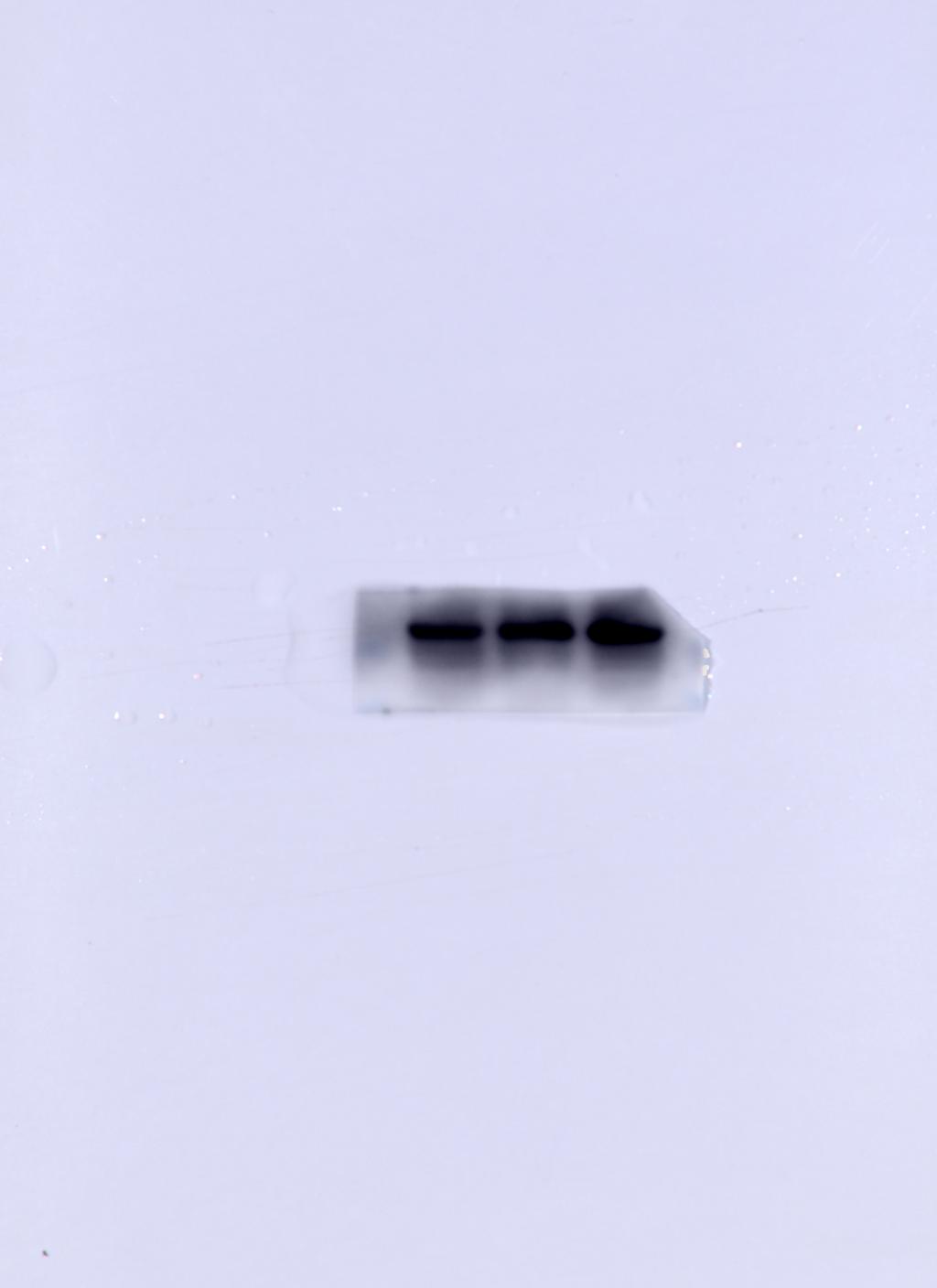

Supplement: Supplemental Information 2 — The CDF files for Fig. 2 are the data of analyzing the fingerprints of 10 batches of the rhizome of M. dauricum Polysaccharide. The software to open this file is the Similarity Evaluation System for Chromatographic Fingerprint of Traditional Chinese Medicine, and the software installation package is available in the Figure 2 folder. The OPJU file in the Figure 3 folder is the data of PCA analysis, and the open software is Origin, which can be downloaded and used for a fee from the Origin official website. [file peerj-10-13946-s002.zip › Raw date/Fig.6/p-NFKB/2/p-NFκB-GAPDH+Marker.jpg]

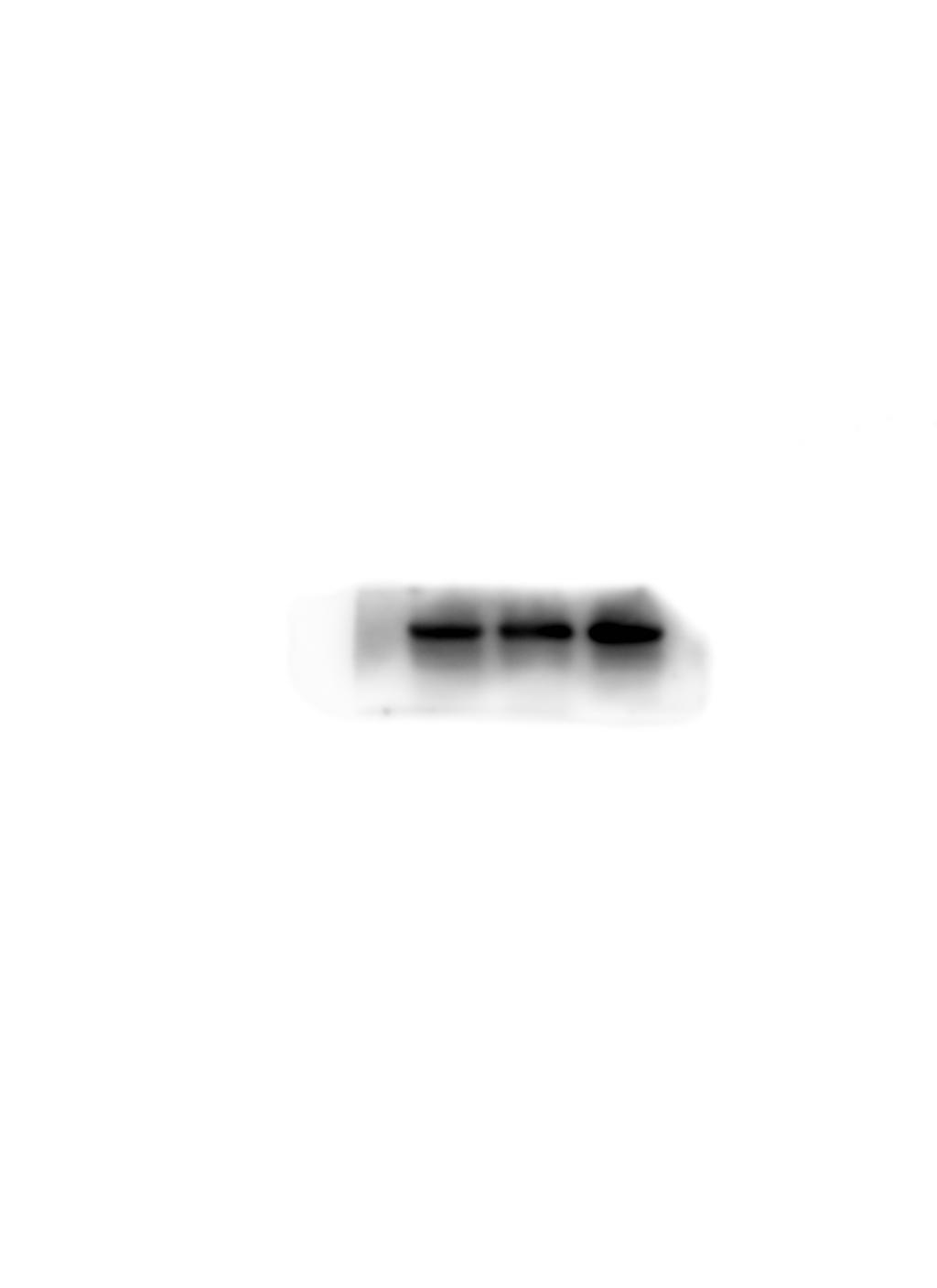

Supplement: Supplemental Information 2 — The CDF files for Fig. 2 are the data of analyzing the fingerprints of 10 batches of the rhizome of M. dauricum Polysaccharide. The software to open this file is the Similarity Evaluation System for Chromatographic Fingerprint of Traditional Chinese Medicine, and the software installation package is available in the Figure 2 folder. The OPJU file in the Figure 3 folder is the data of PCA analysis, and the open software is Origin, which can be downloaded and used for a fee from the Origin official website. [file peerj-10-13946-s002.zip › Raw date/Fig.6/p-NFKB/2/p-NFκB-GAPDH.jpg]

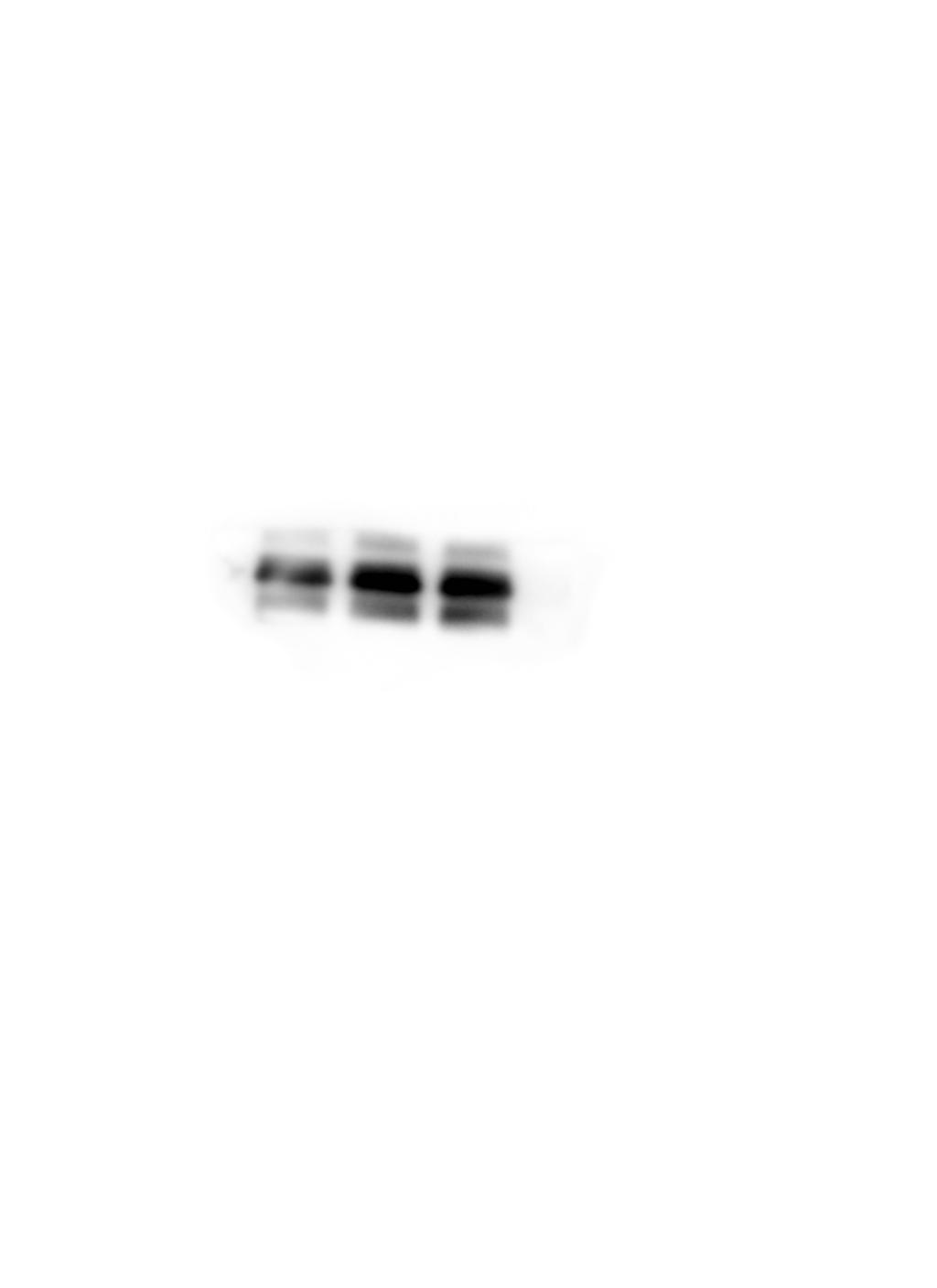

Supplement: Supplemental Information 2 — The CDF files for Fig. 2 are the data of analyzing the fingerprints of 10 batches of the rhizome of M. dauricum Polysaccharide. The software to open this file is the Similarity Evaluation System for Chromatographic Fingerprint of Traditional Chinese Medicine, and the software installation package is available in the Figure 2 folder. The OPJU file in the Figure 3 folder is the data of PCA analysis, and the open software is Origin, which can be downloaded and used for a fee from the Origin official website. [file peerj-10-13946-s002.zip › Raw date/Fig.6/p-NFKB/2/p-NFκB.jpg]

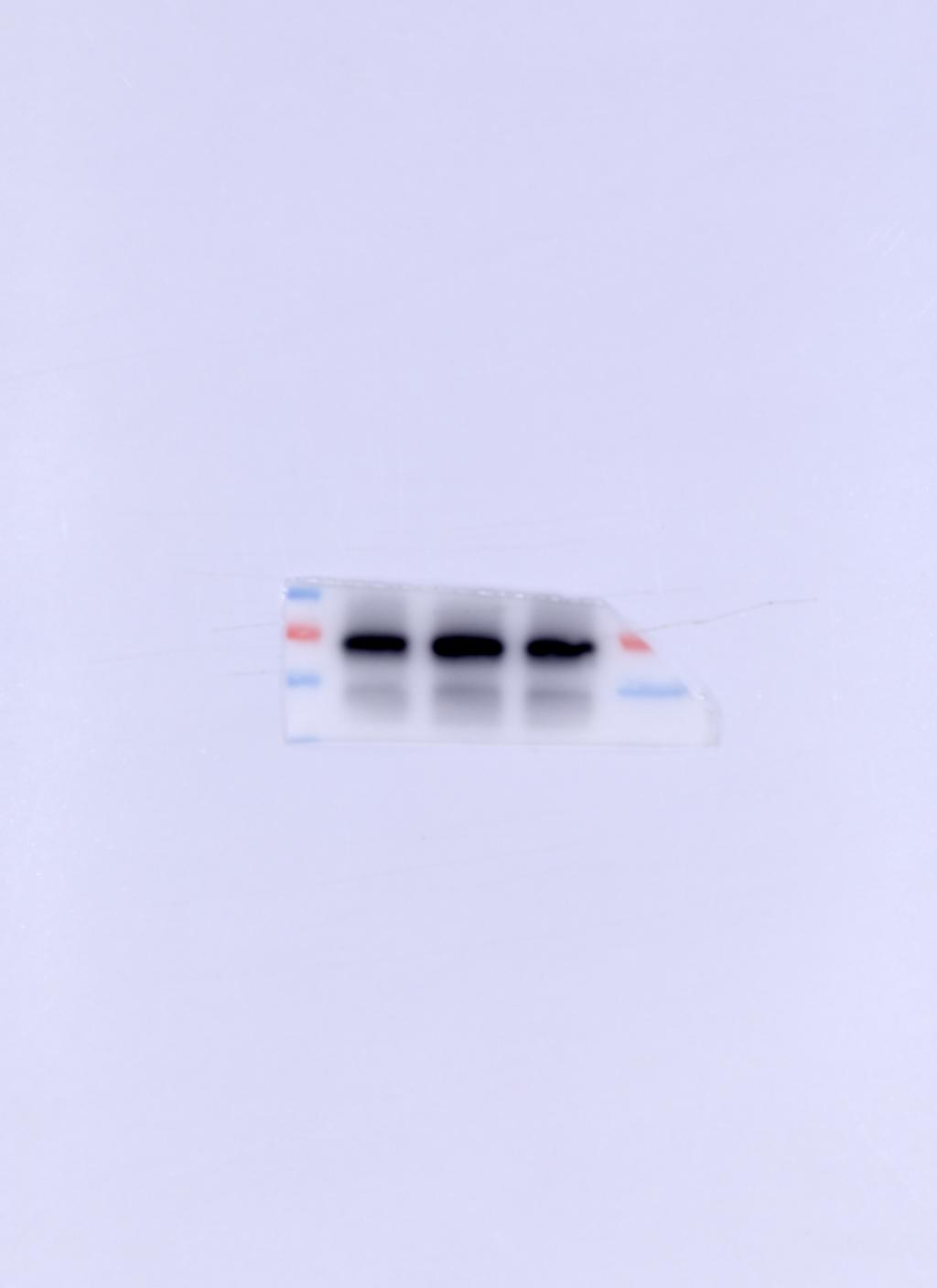

Supplement: Supplemental Information 2 — The CDF files for Fig. 2 are the data of analyzing the fingerprints of 10 batches of the rhizome of M. dauricum Polysaccharide. The software to open this file is the Similarity Evaluation System for Chromatographic Fingerprint of Traditional Chinese Medicine, and the software installation package is available in the Figure 2 folder. The OPJU file in the Figure 3 folder is the data of PCA analysis, and the open software is Origin, which can be downloaded and used for a fee from the Origin official website. [file peerj-10-13946-s002.zip › Raw date/Fig.6/p-NFKB/3/NFκB+Marker.jpg]

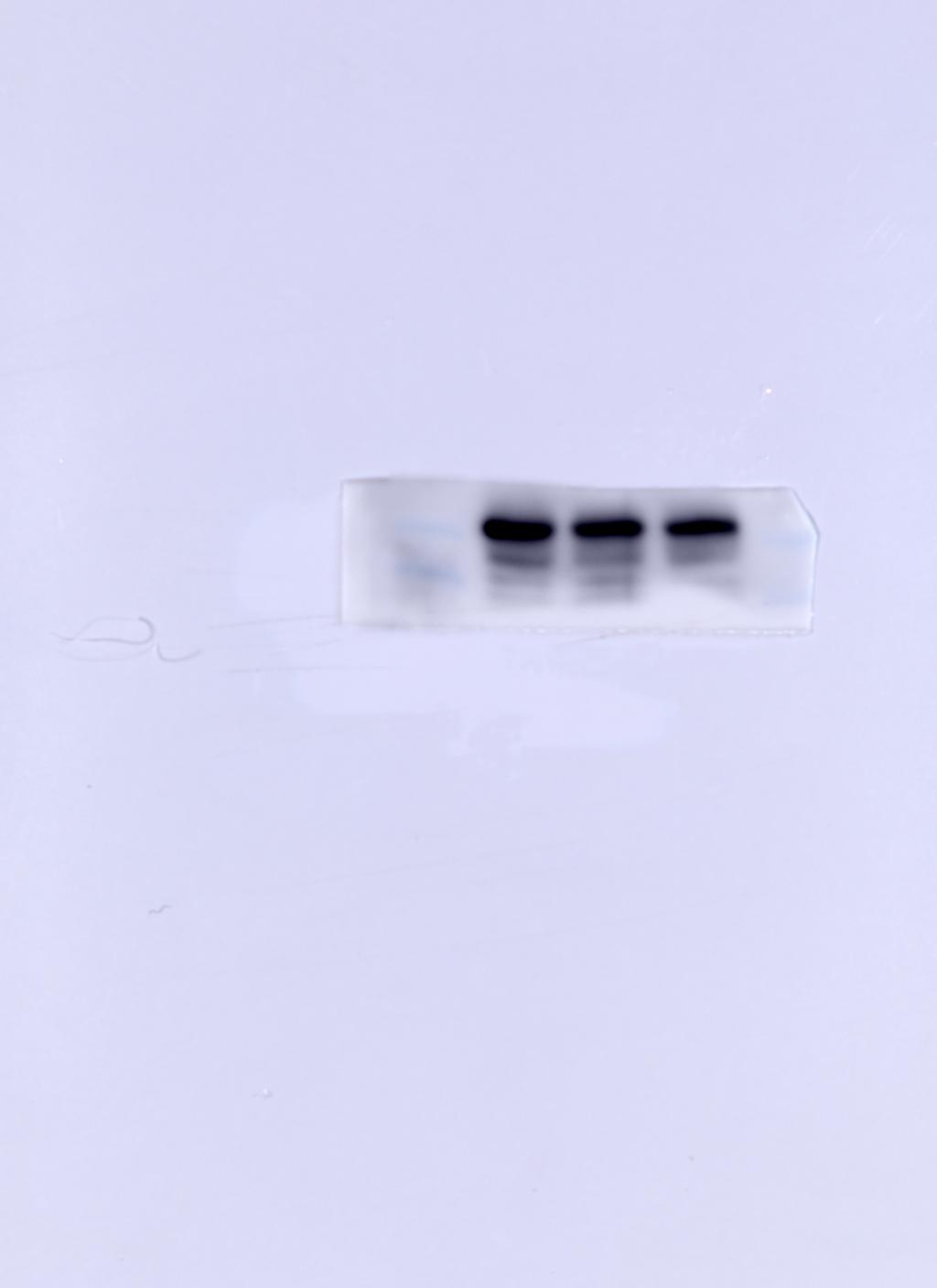

Supplement: Supplemental Information 2 — The CDF files for Fig. 2 are the data of analyzing the fingerprints of 10 batches of the rhizome of M. dauricum Polysaccharide. The software to open this file is the Similarity Evaluation System for Chromatographic Fingerprint of Traditional Chinese Medicine, and the software installation package is available in the Figure 2 folder. The OPJU file in the Figure 3 folder is the data of PCA analysis, and the open software is Origin, which can be downloaded and used for a fee from the Origin official website. [file peerj-10-13946-s002.zip › Raw date/Fig.6/p-NFKB/3/NFκB-GAPDH+Marker.jpg]

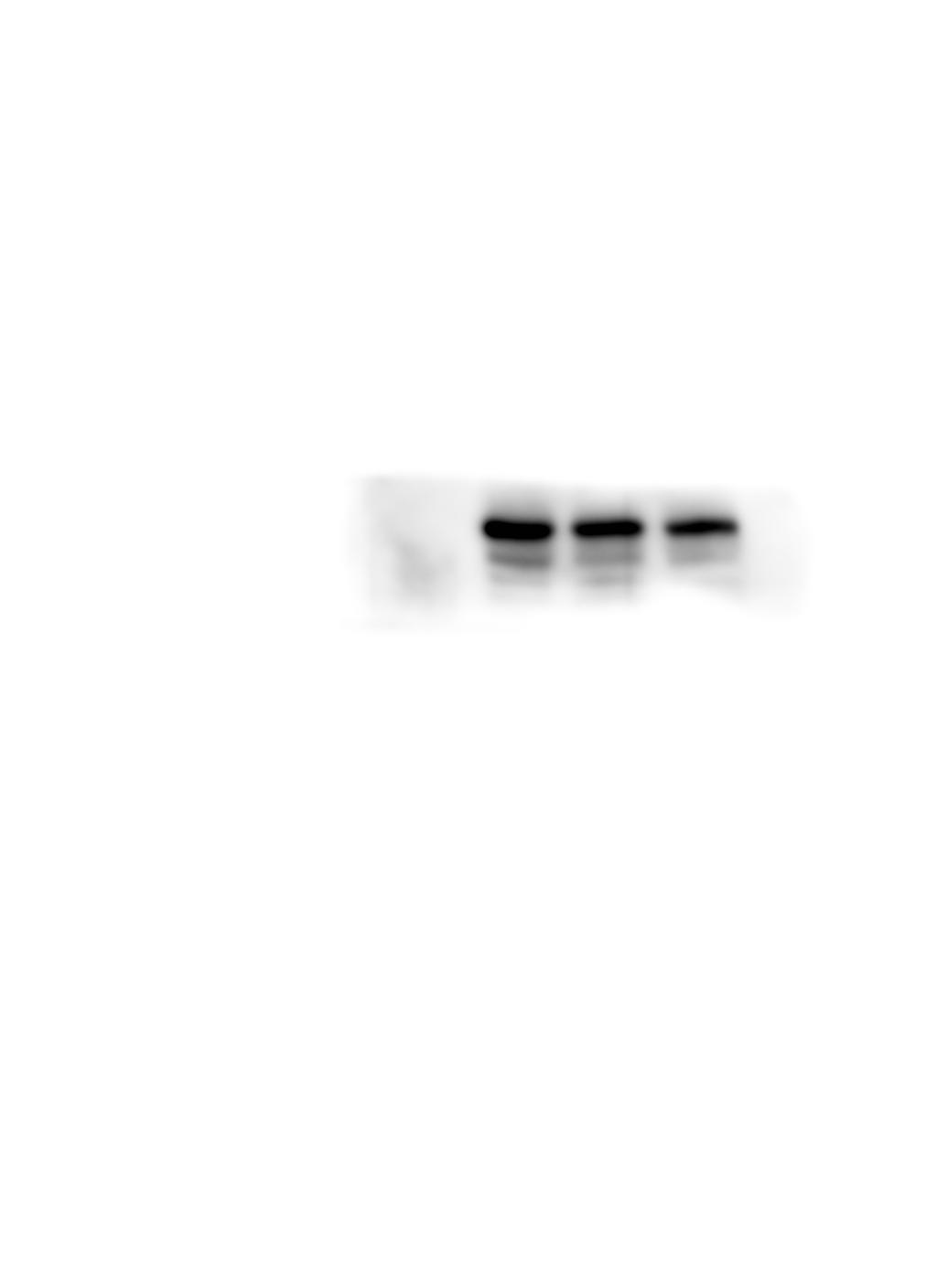

Supplement: Supplemental Information 2 — The CDF files for Fig. 2 are the data of analyzing the fingerprints of 10 batches of the rhizome of M. dauricum Polysaccharide. The software to open this file is the Similarity Evaluation System for Chromatographic Fingerprint of Traditional Chinese Medicine, and the software installation package is available in the Figure 2 folder. The OPJU file in the Figure 3 folder is the data of PCA analysis, and the open software is Origin, which can be downloaded and used for a fee from the Origin official website. [file peerj-10-13946-s002.zip › Raw date/Fig.6/p-NFKB/3/NFκB-GAPDH.jpg]

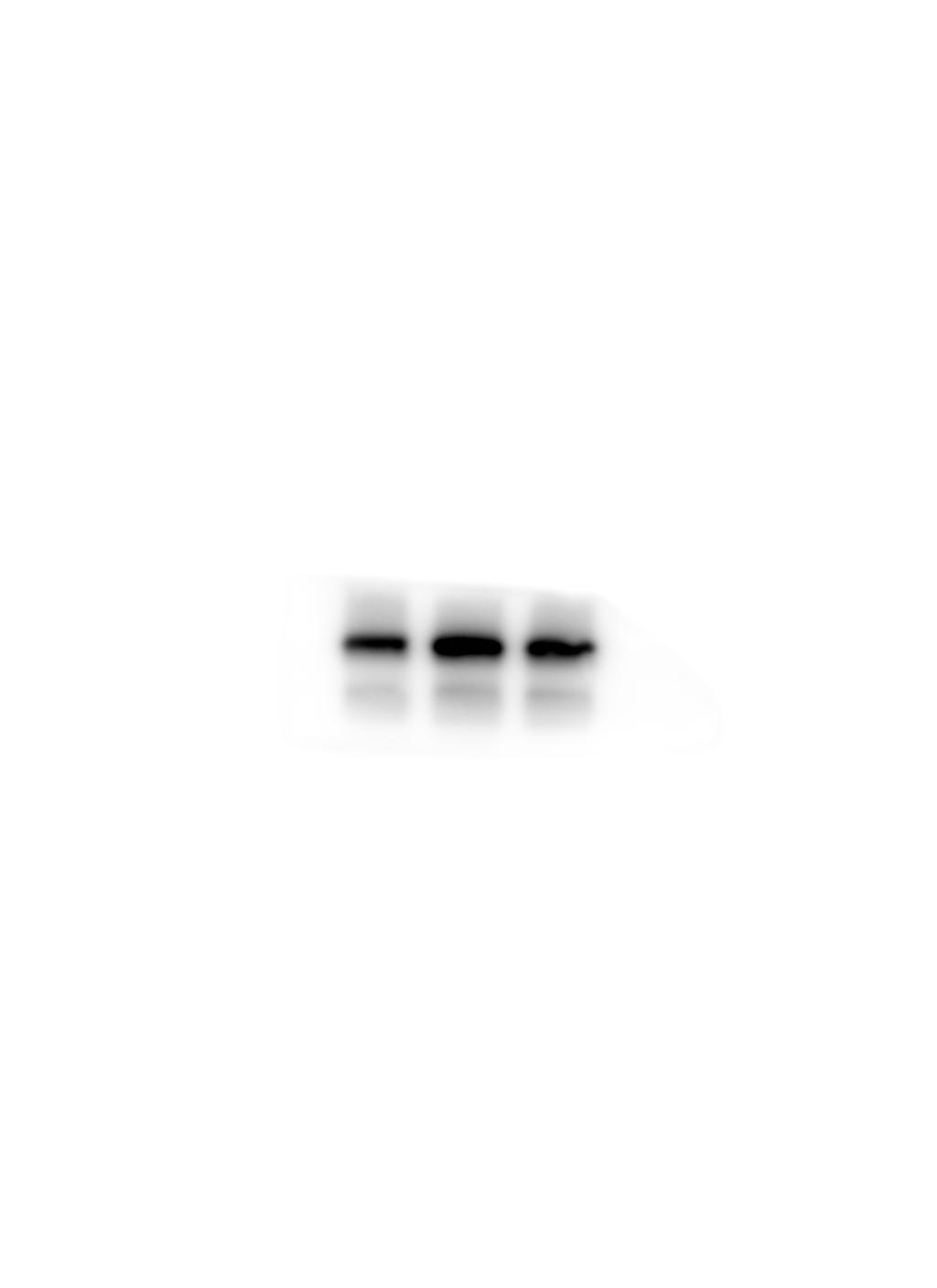

Supplement: Supplemental Information 2 — The CDF files for Fig. 2 are the data of analyzing the fingerprints of 10 batches of the rhizome of M. dauricum Polysaccharide. The software to open this file is the Similarity Evaluation System for Chromatographic Fingerprint of Traditional Chinese Medicine, and the software installation package is available in the Figure 2 folder. The OPJU file in the Figure 3 folder is the data of PCA analysis, and the open software is Origin, which can be downloaded and used for a fee from the Origin official website. [file peerj-10-13946-s002.zip › Raw date/Fig.6/p-NFKB/3/NFκB.jpg]

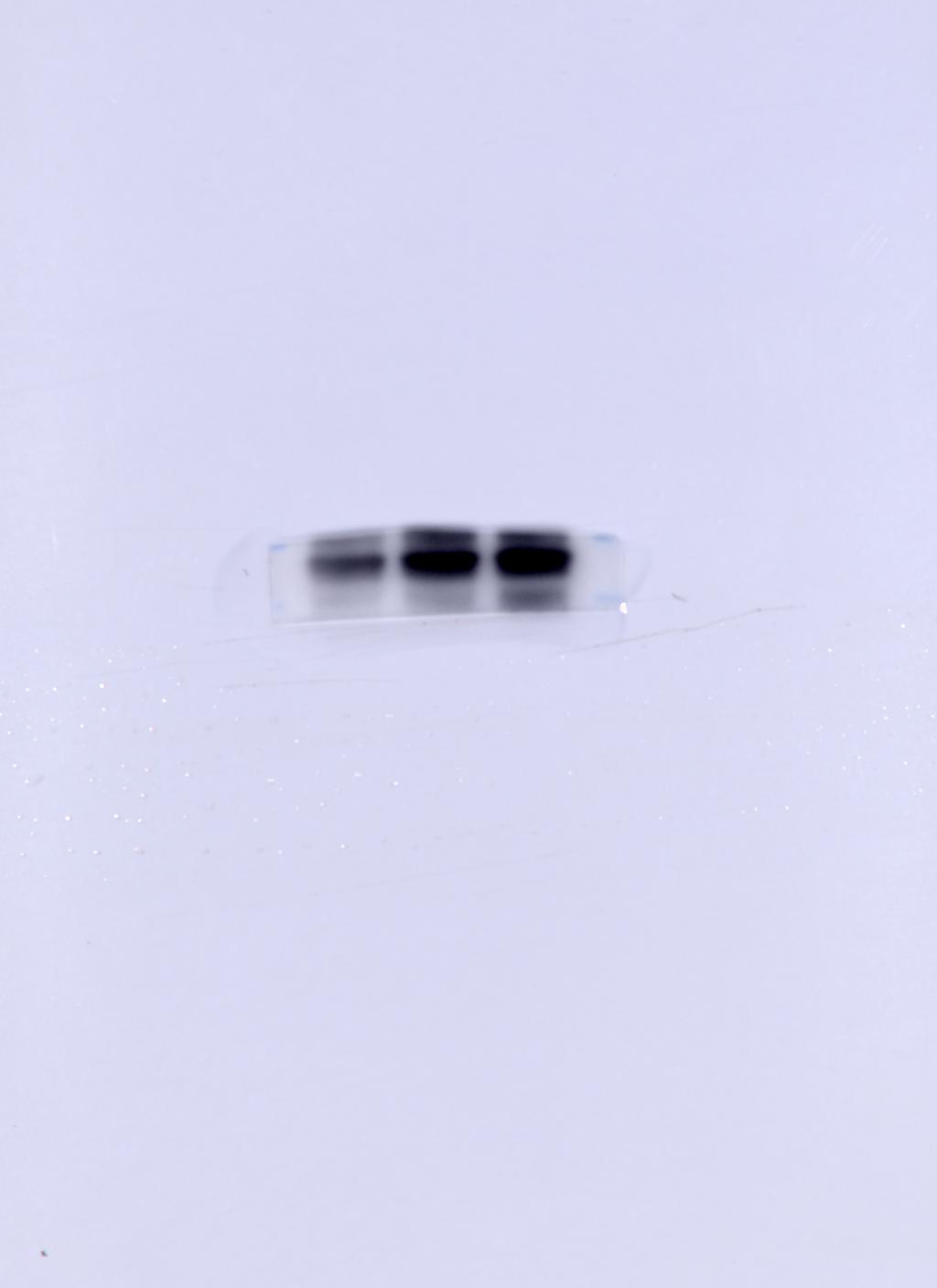

Supplement: Supplemental Information 2 — The CDF files for Fig. 2 are the data of analyzing the fingerprints of 10 batches of the rhizome of M. dauricum Polysaccharide. The software to open this file is the Similarity Evaluation System for Chromatographic Fingerprint of Traditional Chinese Medicine, and the software installation package is available in the Figure 2 folder. The OPJU file in the Figure 3 folder is the data of PCA analysis, and the open software is Origin, which can be downloaded and used for a fee from the Origin official website. [file peerj-10-13946-s002.zip › Raw date/Fig.6/p-NFKB/3/p-NFκB+Marker.jpg]

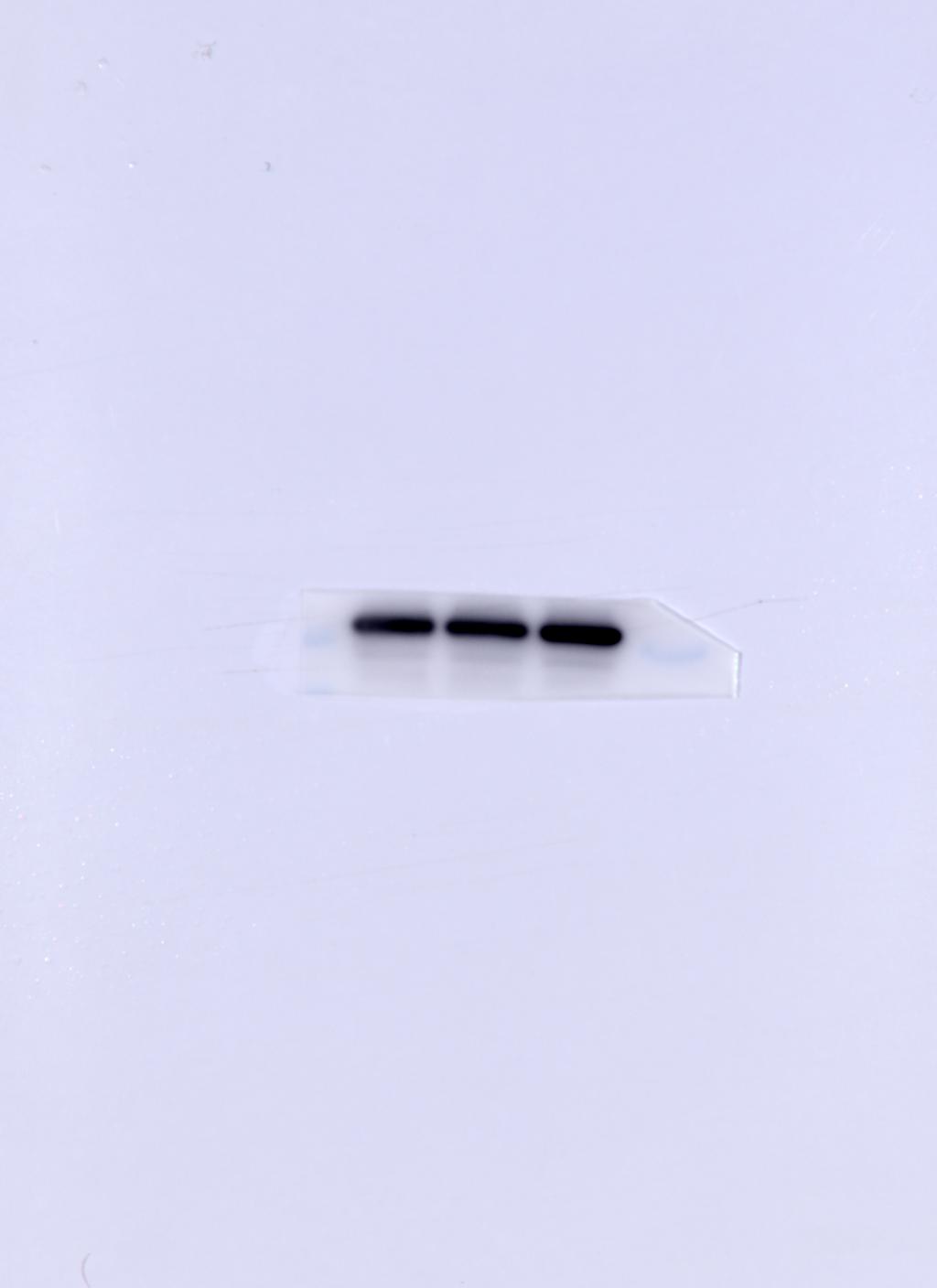

Supplement: Supplemental Information 2 — The CDF files for Fig. 2 are the data of analyzing the fingerprints of 10 batches of the rhizome of M. dauricum Polysaccharide. The software to open this file is the Similarity Evaluation System for Chromatographic Fingerprint of Traditional Chinese Medicine, and the software installation package is available in the Figure 2 folder. The OPJU file in the Figure 3 folder is the data of PCA analysis, and the open software is Origin, which can be downloaded and used for a fee from the Origin official website. [file peerj-10-13946-s002.zip › Raw date/Fig.6/p-NFKB/3/p-NFκB-GAPDH+Marker.jpg]

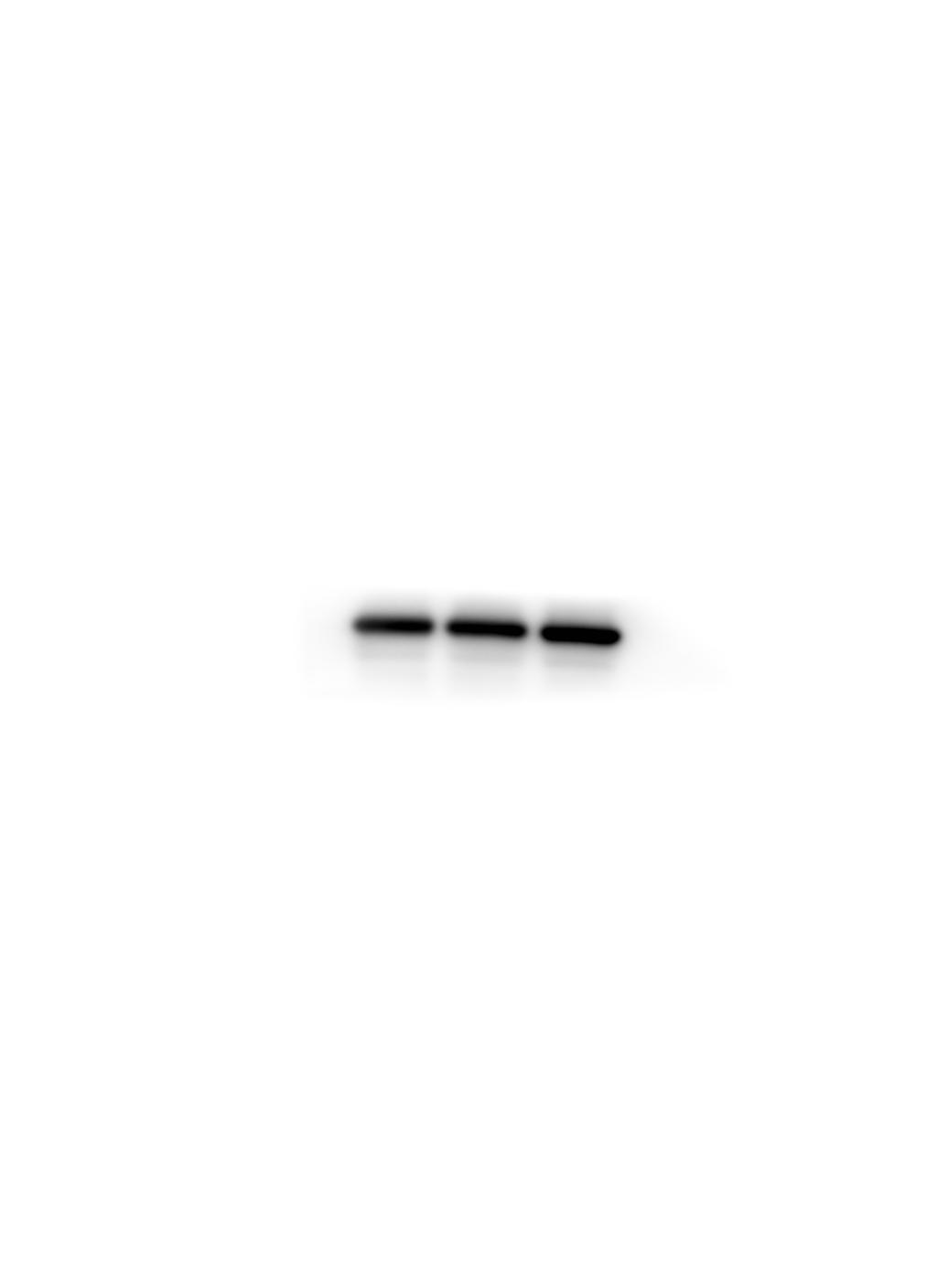

Supplement: Supplemental Information 2 — The CDF files for Fig. 2 are the data of analyzing the fingerprints of 10 batches of the rhizome of M. dauricum Polysaccharide. The software to open this file is the Similarity Evaluation System for Chromatographic Fingerprint of Traditional Chinese Medicine, and the software installation package is available in the Figure 2 folder. The OPJU file in the Figure 3 folder is the data of PCA analysis, and the open software is Origin, which can be downloaded and used for a fee from the Origin official website. [file peerj-10-13946-s002.zip › Raw date/Fig.6/p-NFKB/3/p-NFκB-GAPDH.jpg]

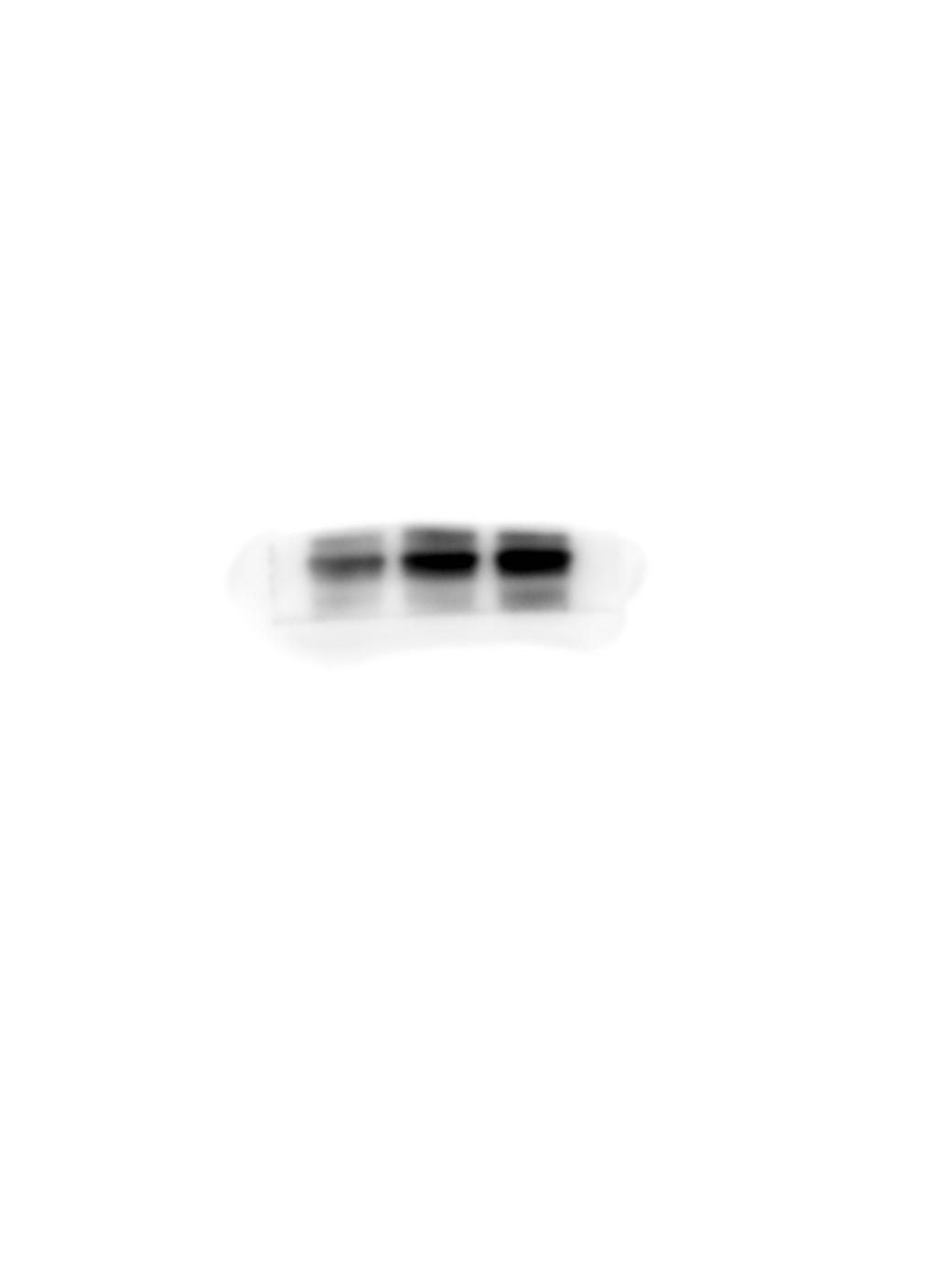

Supplement: Supplemental Information 2 — The CDF files for Fig. 2 are the data of analyzing the fingerprints of 10 batches of the rhizome of M. dauricum Polysaccharide. The software to open this file is the Similarity Evaluation System for Chromatographic Fingerprint of Traditional Chinese Medicine, and the software installation package is available in the Figure 2 folder. The OPJU file in the Figure 3 folder is the data of PCA analysis, and the open software is Origin, which can be downloaded and used for a fee from the Origin official website. [file peerj-10-13946-s002.zip › Raw date/Fig.6/p-NFKB/3/p-NFκB.jpg]

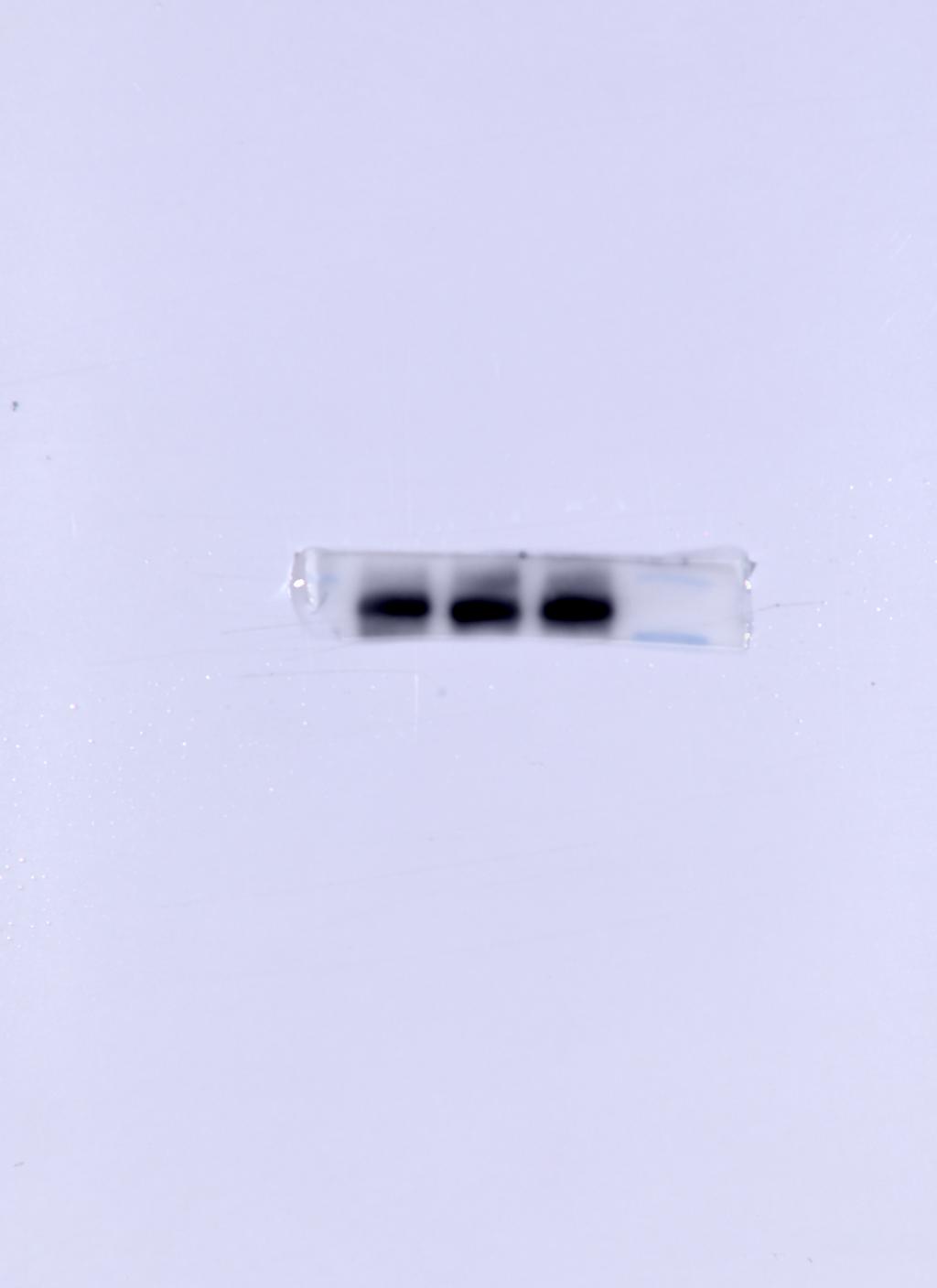

Supplement: Supplemental Information 2 — The CDF files for Fig. 2 are the data of analyzing the fingerprints of 10 batches of the rhizome of M. dauricum Polysaccharide. The software to open this file is the Similarity Evaluation System for Chromatographic Fingerprint of Traditional Chinese Medicine, and the software installation package is available in the Figure 2 folder. The OPJU file in the Figure 3 folder is the data of PCA analysis, and the open software is Origin, which can be downloaded and used for a fee from the Origin official website. [file peerj-10-13946-s002.zip › Raw date/Fig.6/p-P38/1/p-P38+Marker.jpg]

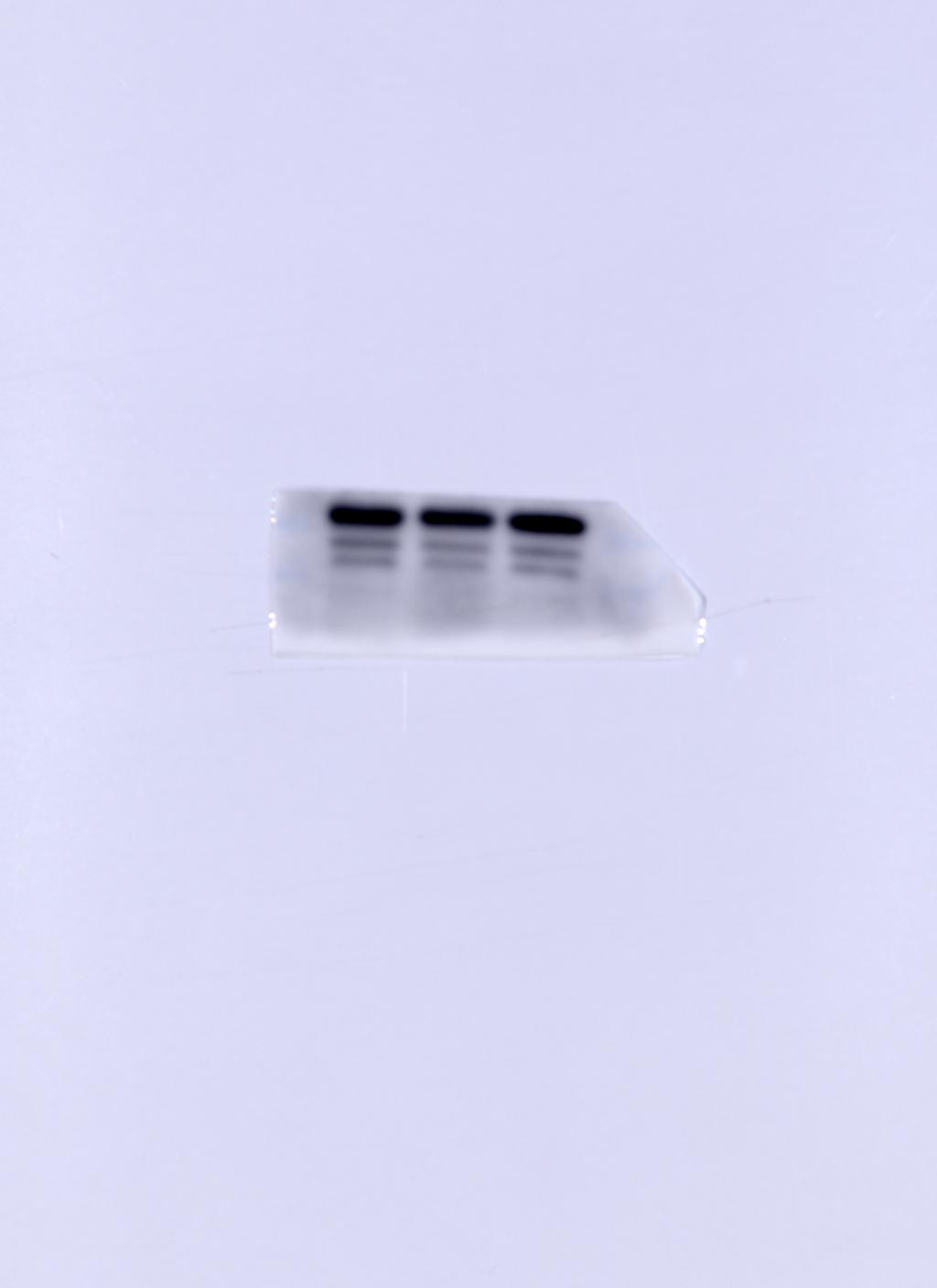

Supplement: Supplemental Information 2 — The CDF files for Fig. 2 are the data of analyzing the fingerprints of 10 batches of the rhizome of M. dauricum Polysaccharide. The software to open this file is the Similarity Evaluation System for Chromatographic Fingerprint of Traditional Chinese Medicine, and the software installation package is available in the Figure 2 folder. The OPJU file in the Figure 3 folder is the data of PCA analysis, and the open software is Origin, which can be downloaded and used for a fee from the Origin official website. [file peerj-10-13946-s002.zip › Raw date/Fig.6/p-P38/1/p-P38-GAPDH+Marker.jpg]

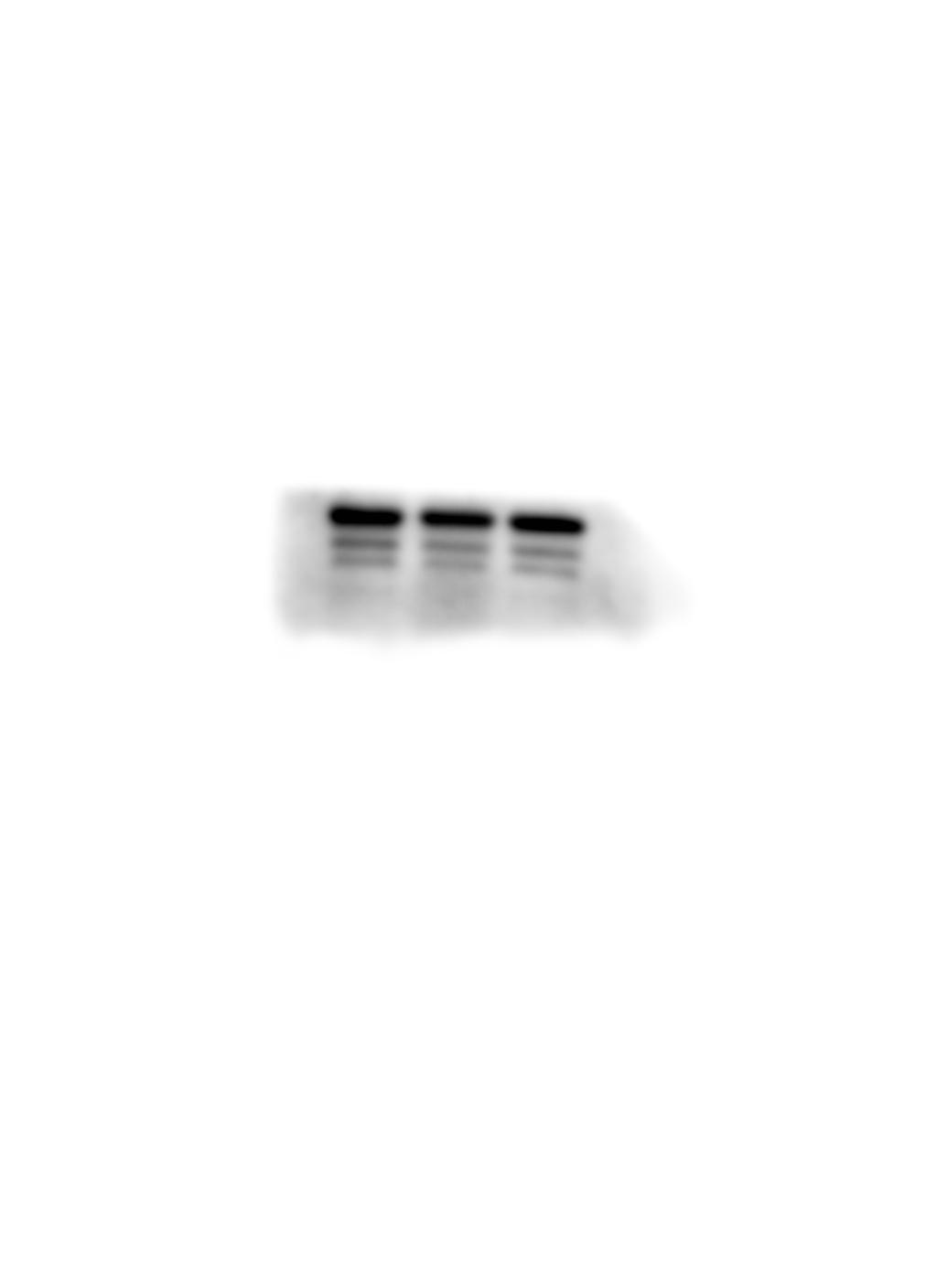

Supplement: Supplemental Information 2 — The CDF files for Fig. 2 are the data of analyzing the fingerprints of 10 batches of the rhizome of M. dauricum Polysaccharide. The software to open this file is the Similarity Evaluation System for Chromatographic Fingerprint of Traditional Chinese Medicine, and the software installation package is available in the Figure 2 folder. The OPJU file in the Figure 3 folder is the data of PCA analysis, and the open software is Origin, which can be downloaded and used for a fee from the Origin official website. [file peerj-10-13946-s002.zip › Raw date/Fig.6/p-P38/1/p-P38-GAPDH.jpg]

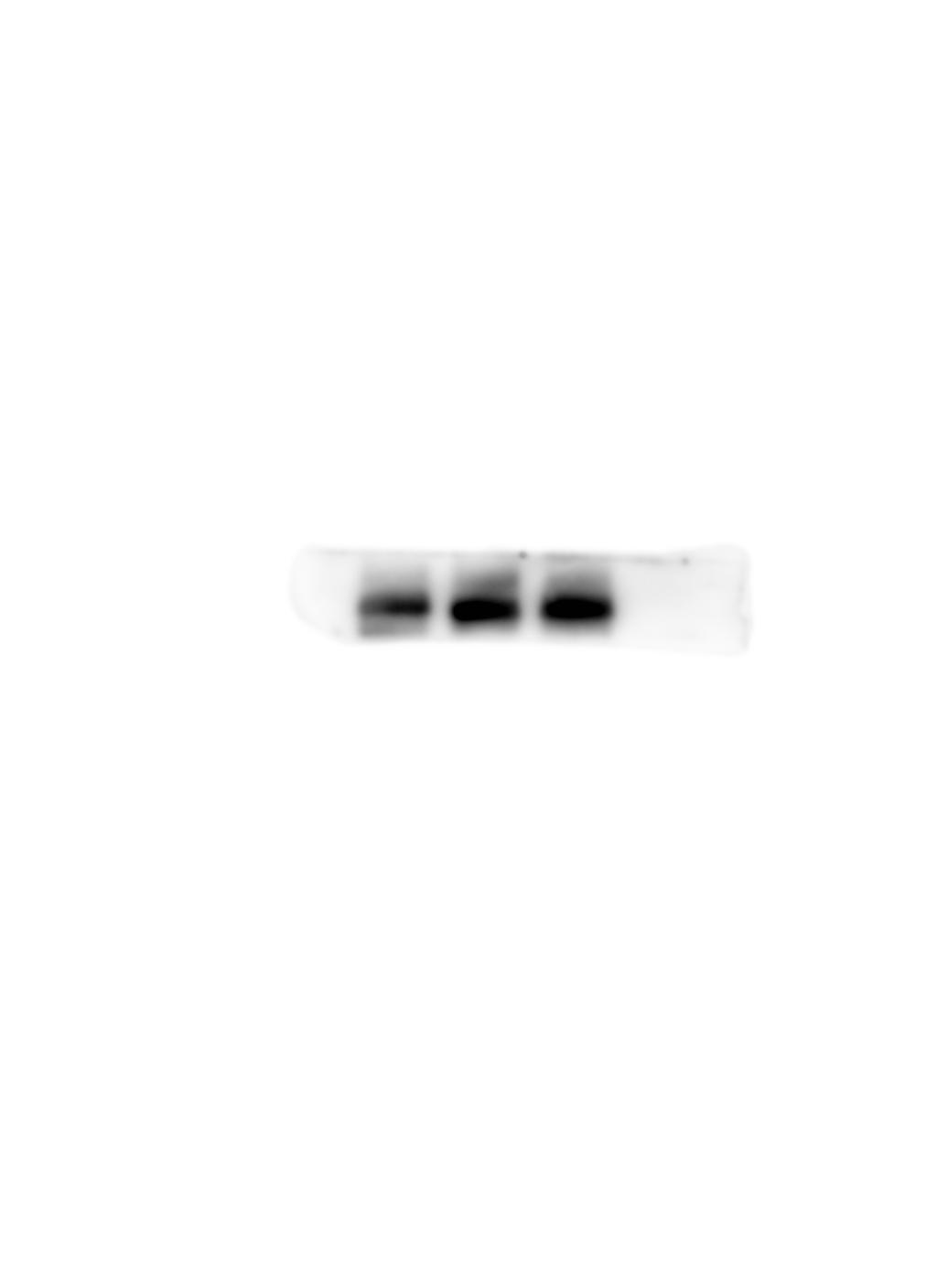

Supplement: Supplemental Information 2 — The CDF files for Fig. 2 are the data of analyzing the fingerprints of 10 batches of the rhizome of M. dauricum Polysaccharide. The software to open this file is the Similarity Evaluation System for Chromatographic Fingerprint of Traditional Chinese Medicine, and the software installation package is available in the Figure 2 folder. The OPJU file in the Figure 3 folder is the data of PCA analysis, and the open software is Origin, which can be downloaded and used for a fee from the Origin official website. [file peerj-10-13946-s002.zip › Raw date/Fig.6/p-P38/1/p-P38.jpg]

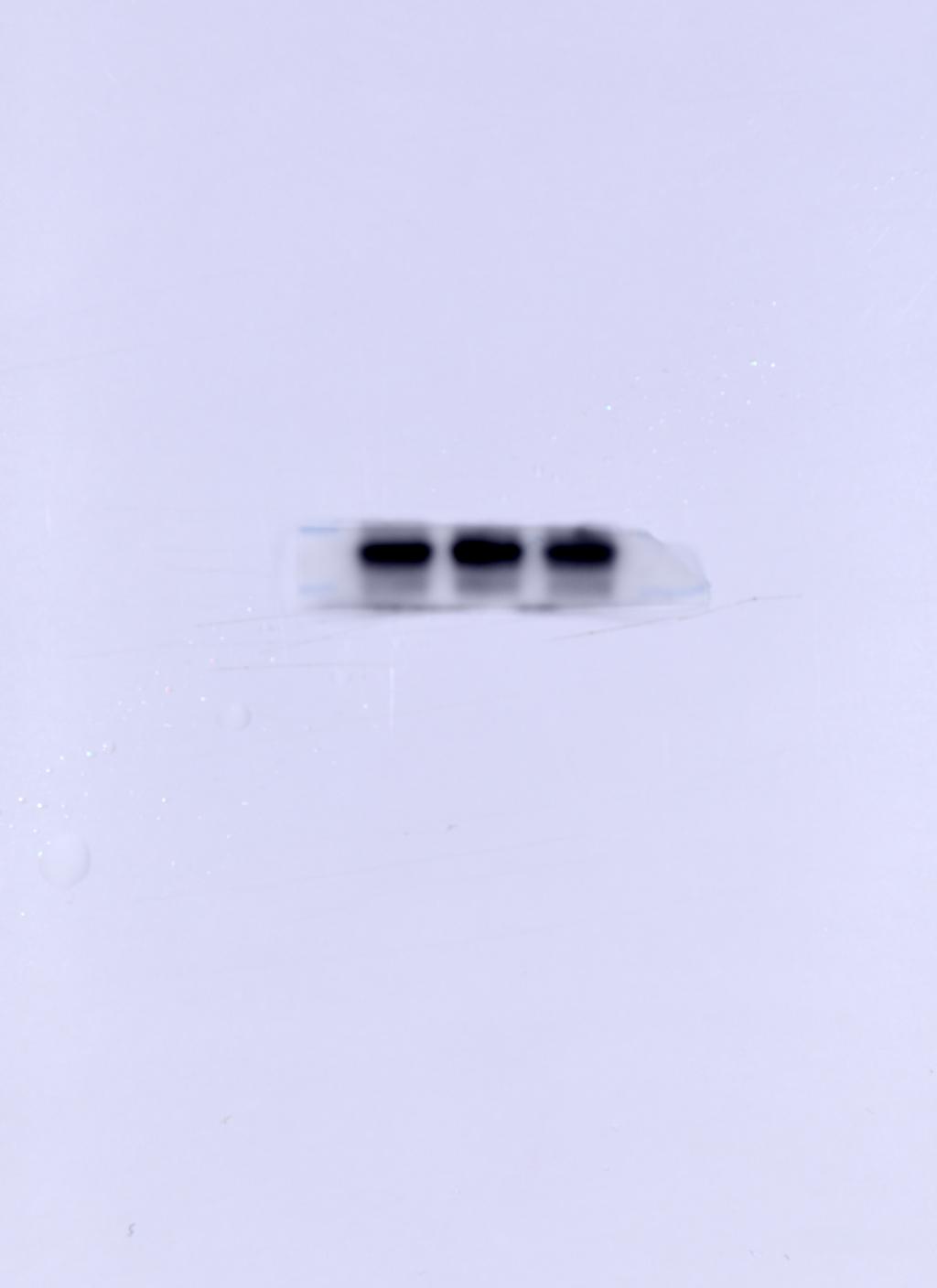

Supplement: Supplemental Information 2 — The CDF files for Fig. 2 are the data of analyzing the fingerprints of 10 batches of the rhizome of M. dauricum Polysaccharide. The software to open this file is the Similarity Evaluation System for Chromatographic Fingerprint of Traditional Chinese Medicine, and the software installation package is available in the Figure 2 folder. The OPJU file in the Figure 3 folder is the data of PCA analysis, and the open software is Origin, which can be downloaded and used for a fee from the Origin official website. [file peerj-10-13946-s002.zip › Raw date/Fig.6/p-P38/1/P38+Marker.jpg]

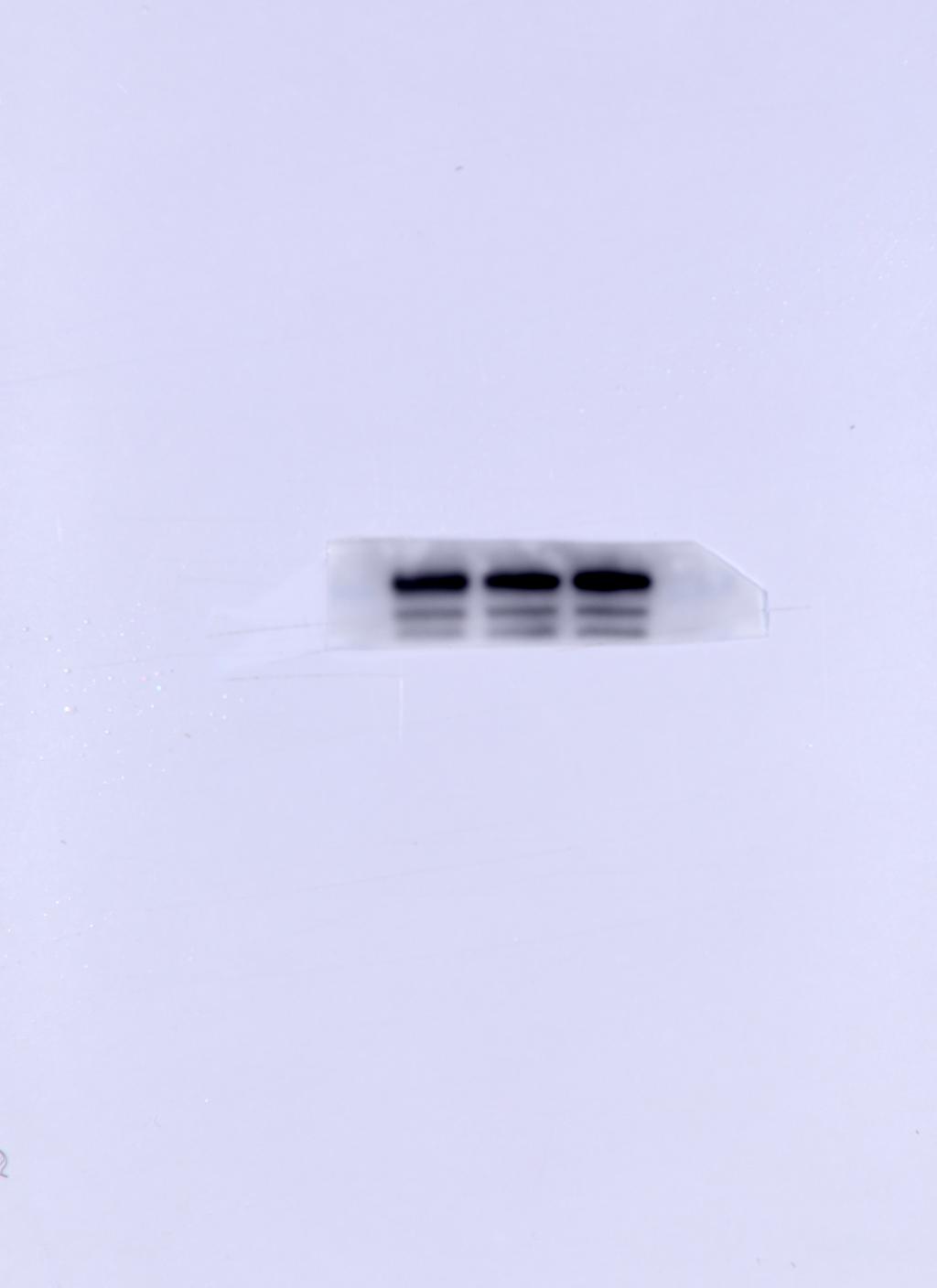

Supplement: Supplemental Information 2 — The CDF files for Fig. 2 are the data of analyzing the fingerprints of 10 batches of the rhizome of M. dauricum Polysaccharide. The software to open this file is the Similarity Evaluation System for Chromatographic Fingerprint of Traditional Chinese Medicine, and the software installation package is available in the Figure 2 folder. The OPJU file in the Figure 3 folder is the data of PCA analysis, and the open software is Origin, which can be downloaded and used for a fee from the Origin official website. [file peerj-10-13946-s002.zip › Raw date/Fig.6/p-P38/1/P38-GAPDH+Marker.jpg]

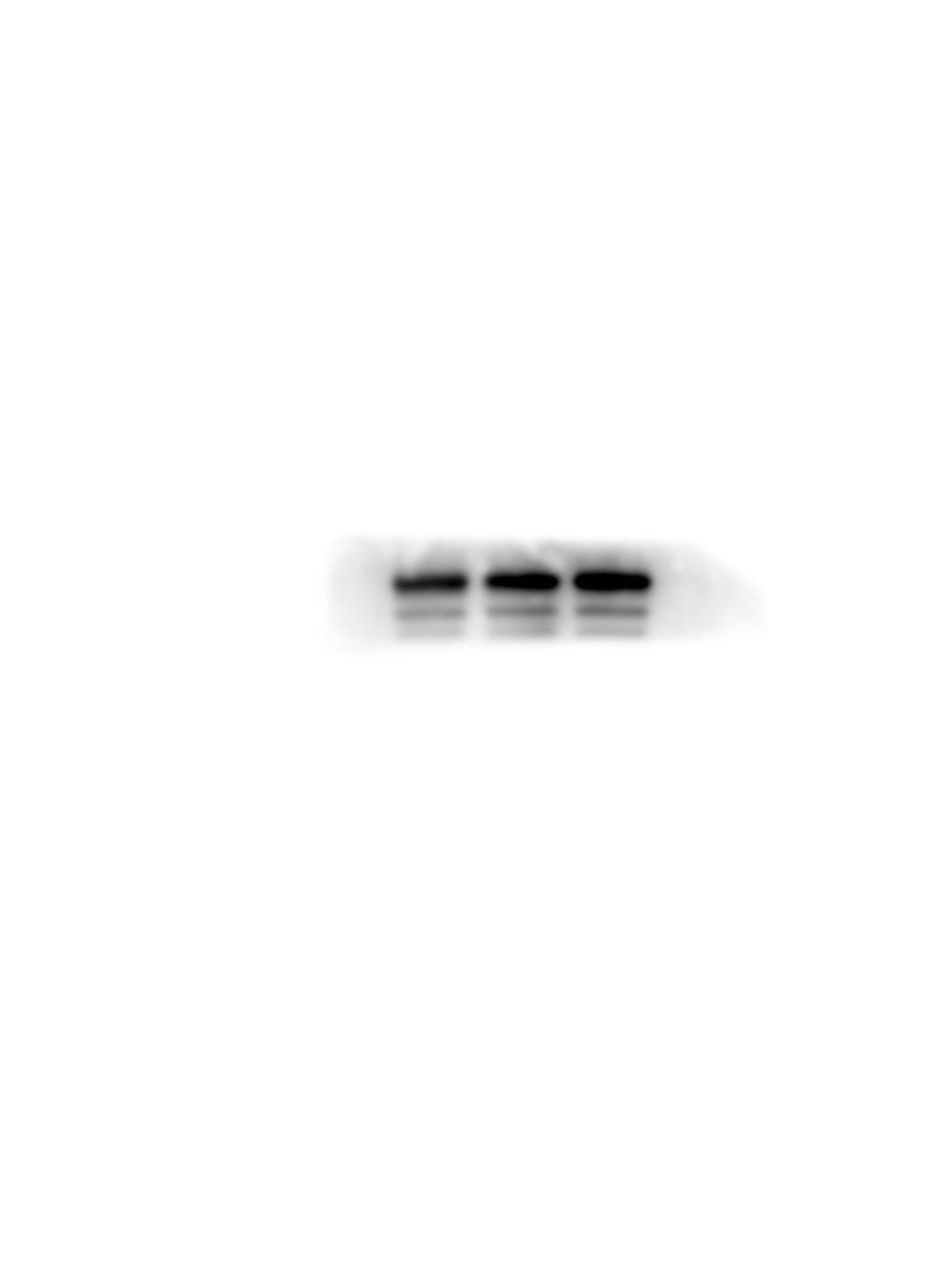

Supplement: Supplemental Information 2 — The CDF files for Fig. 2 are the data of analyzing the fingerprints of 10 batches of the rhizome of M. dauricum Polysaccharide. The software to open this file is the Similarity Evaluation System for Chromatographic Fingerprint of Traditional Chinese Medicine, and the software installation package is available in the Figure 2 folder. The OPJU file in the Figure 3 folder is the data of PCA analysis, and the open software is Origin, which can be downloaded and used for a fee from the Origin official website. [file peerj-10-13946-s002.zip › Raw date/Fig.6/p-P38/1/P38-GAPDH.jpg]

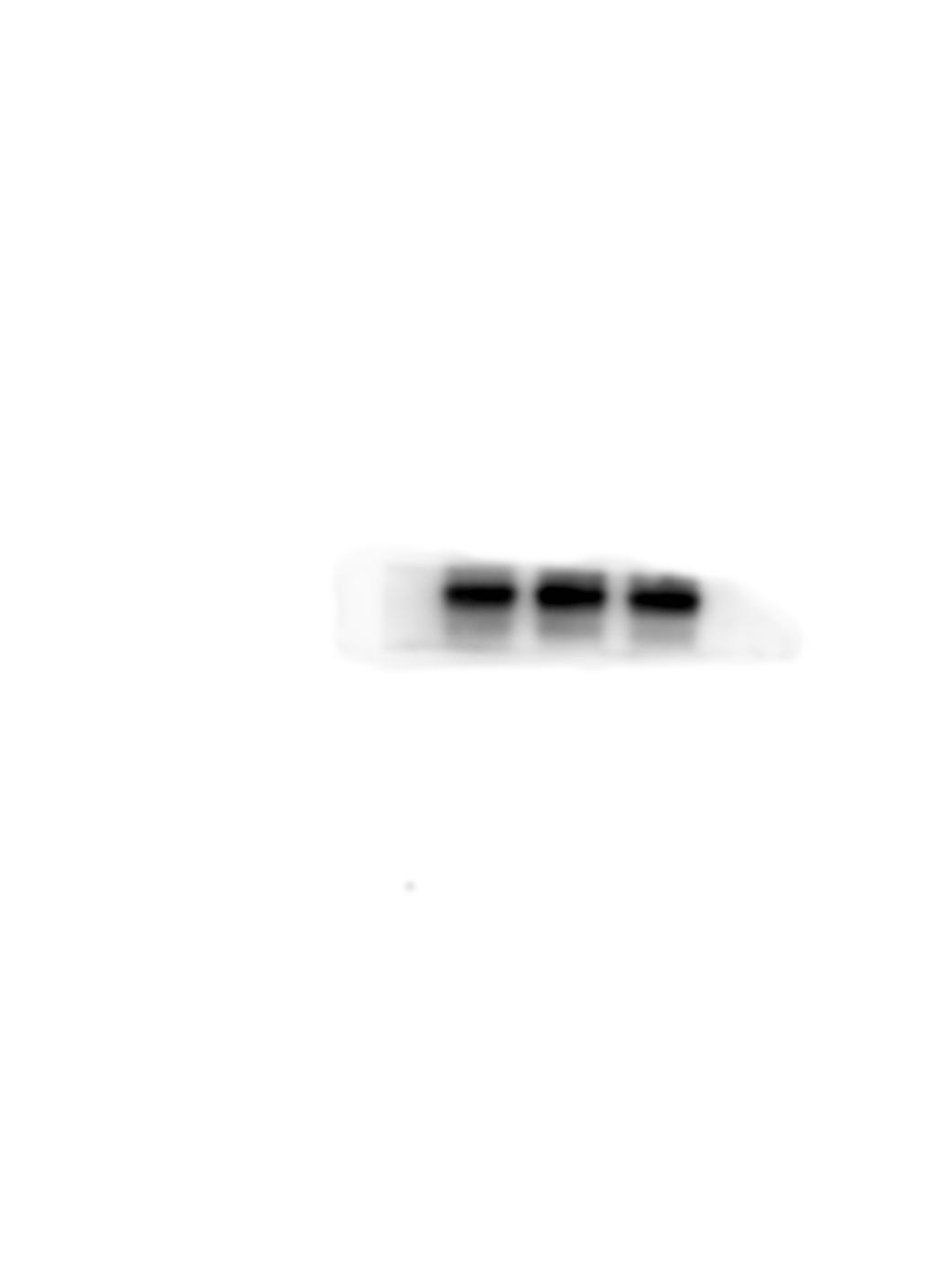

Supplement: Supplemental Information 2 — The CDF files for Fig. 2 are the data of analyzing the fingerprints of 10 batches of the rhizome of M. dauricum Polysaccharide. The software to open this file is the Similarity Evaluation System for Chromatographic Fingerprint of Traditional Chinese Medicine, and the software installation package is available in the Figure 2 folder. The OPJU file in the Figure 3 folder is the data of PCA analysis, and the open software is Origin, which can be downloaded and used for a fee from the Origin official website. [file peerj-10-13946-s002.zip › Raw date/Fig.6/p-P38/1/P38.jpg]

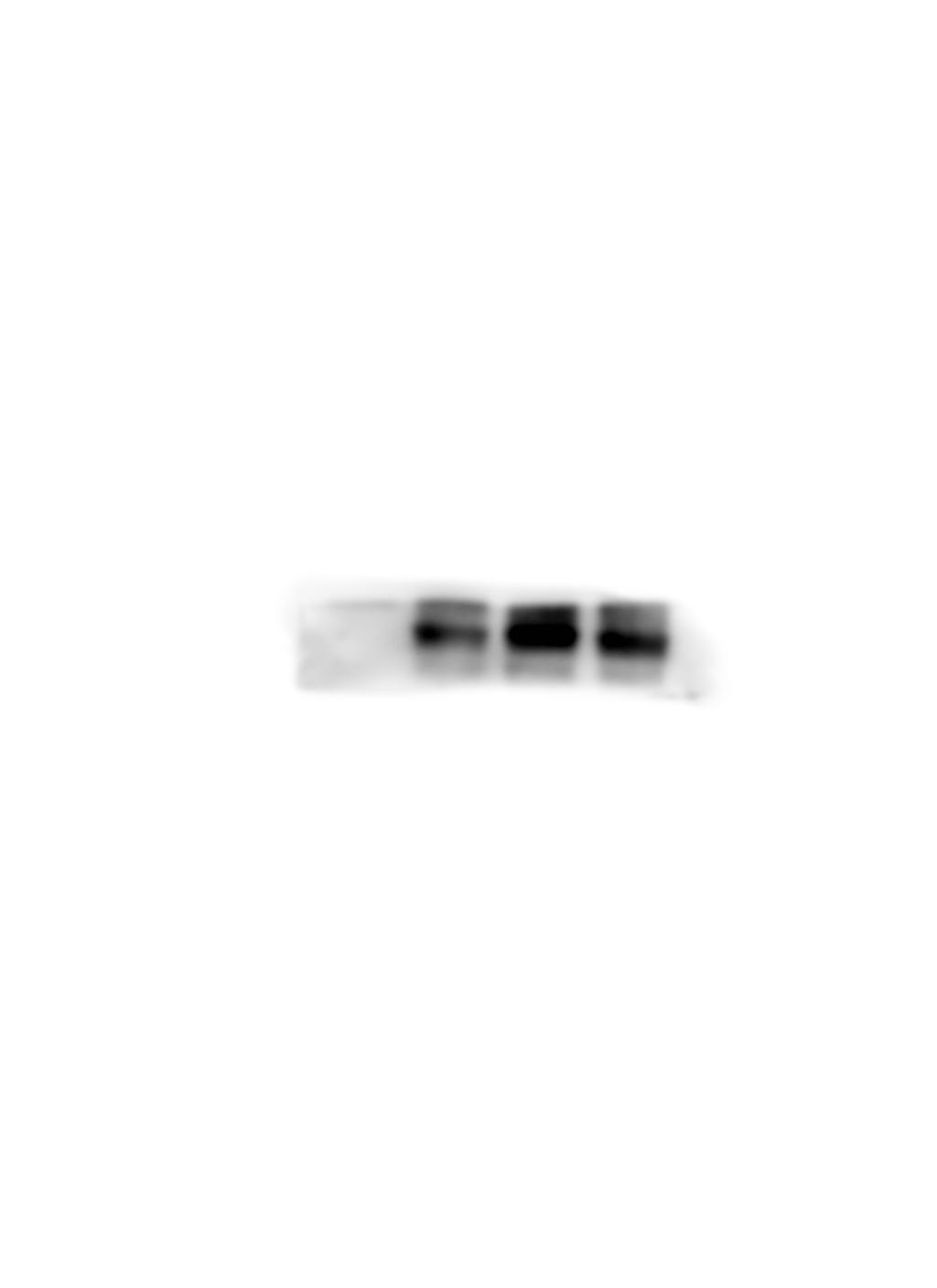

Supplement: Supplemental Information 2 — The CDF files for Fig. 2 are the data of analyzing the fingerprints of 10 batches of the rhizome of M. dauricum Polysaccharide. The software to open this file is the Similarity Evaluation System for Chromatographic Fingerprint of Traditional Chinese Medicine, and the software installation package is available in the Figure 2 folder. The OPJU file in the Figure 3 folder is the data of PCA analysis, and the open software is Origin, which can be downloaded and used for a fee from the Origin official website. [file peerj-10-13946-s002.zip › Raw date/Fig.6/p-P38/2/p-P38 .jpg]

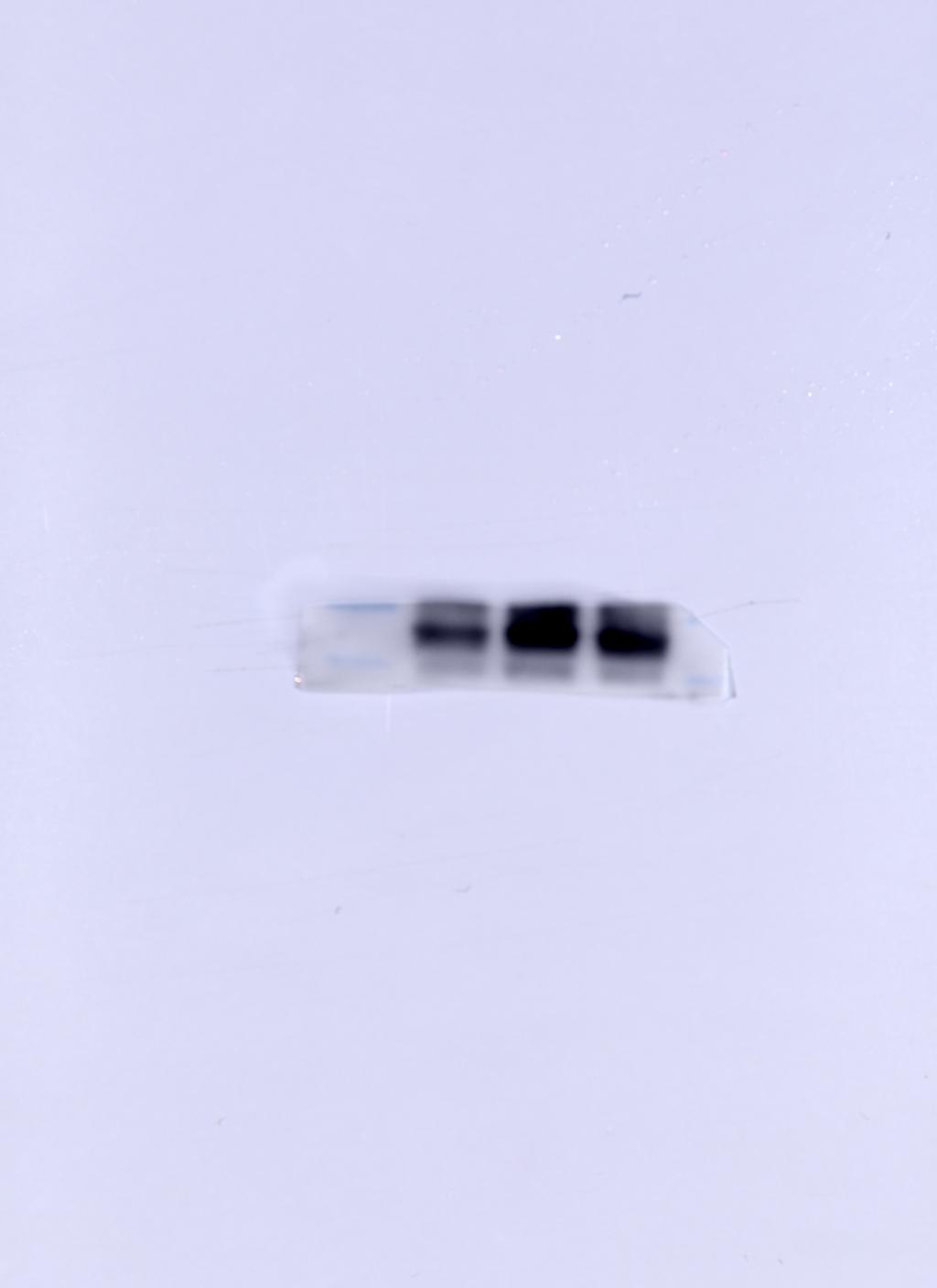

Supplement: Supplemental Information 2 — The CDF files for Fig. 2 are the data of analyzing the fingerprints of 10 batches of the rhizome of M. dauricum Polysaccharide. The software to open this file is the Similarity Evaluation System for Chromatographic Fingerprint of Traditional Chinese Medicine, and the software installation package is available in the Figure 2 folder. The OPJU file in the Figure 3 folder is the data of PCA analysis, and the open software is Origin, which can be downloaded and used for a fee from the Origin official website. [file peerj-10-13946-s002.zip › Raw date/Fig.6/p-P38/2/p-P38+Marker.jpg]

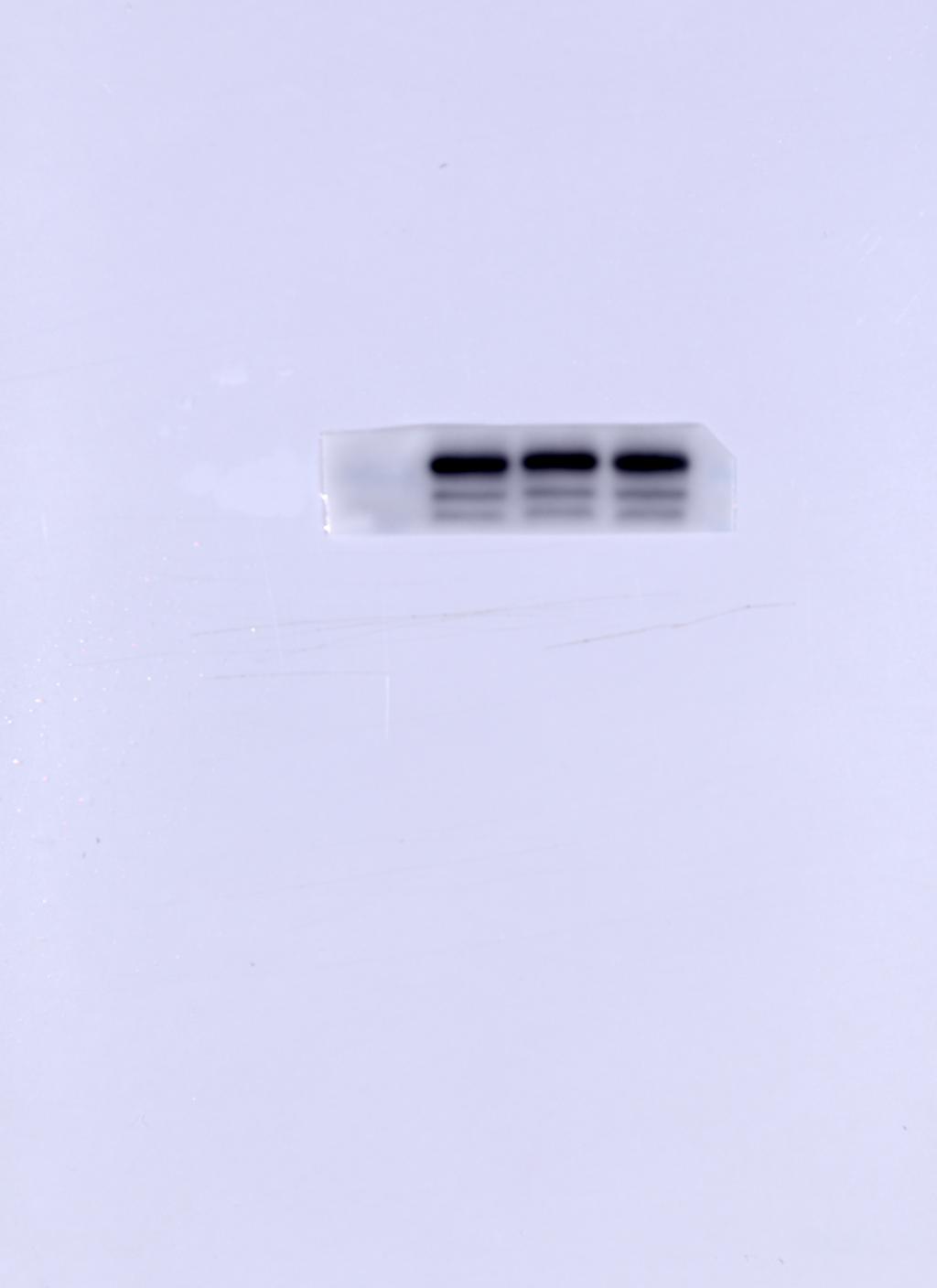

Supplement: Supplemental Information 2 — The CDF files for Fig. 2 are the data of analyzing the fingerprints of 10 batches of the rhizome of M. dauricum Polysaccharide. The software to open this file is the Similarity Evaluation System for Chromatographic Fingerprint of Traditional Chinese Medicine, and the software installation package is available in the Figure 2 folder. The OPJU file in the Figure 3 folder is the data of PCA analysis, and the open software is Origin, which can be downloaded and used for a fee from the Origin official website. [file peerj-10-13946-s002.zip › Raw date/Fig.6/p-P38/2/p-P38-GAPDH+Marker.jpg]

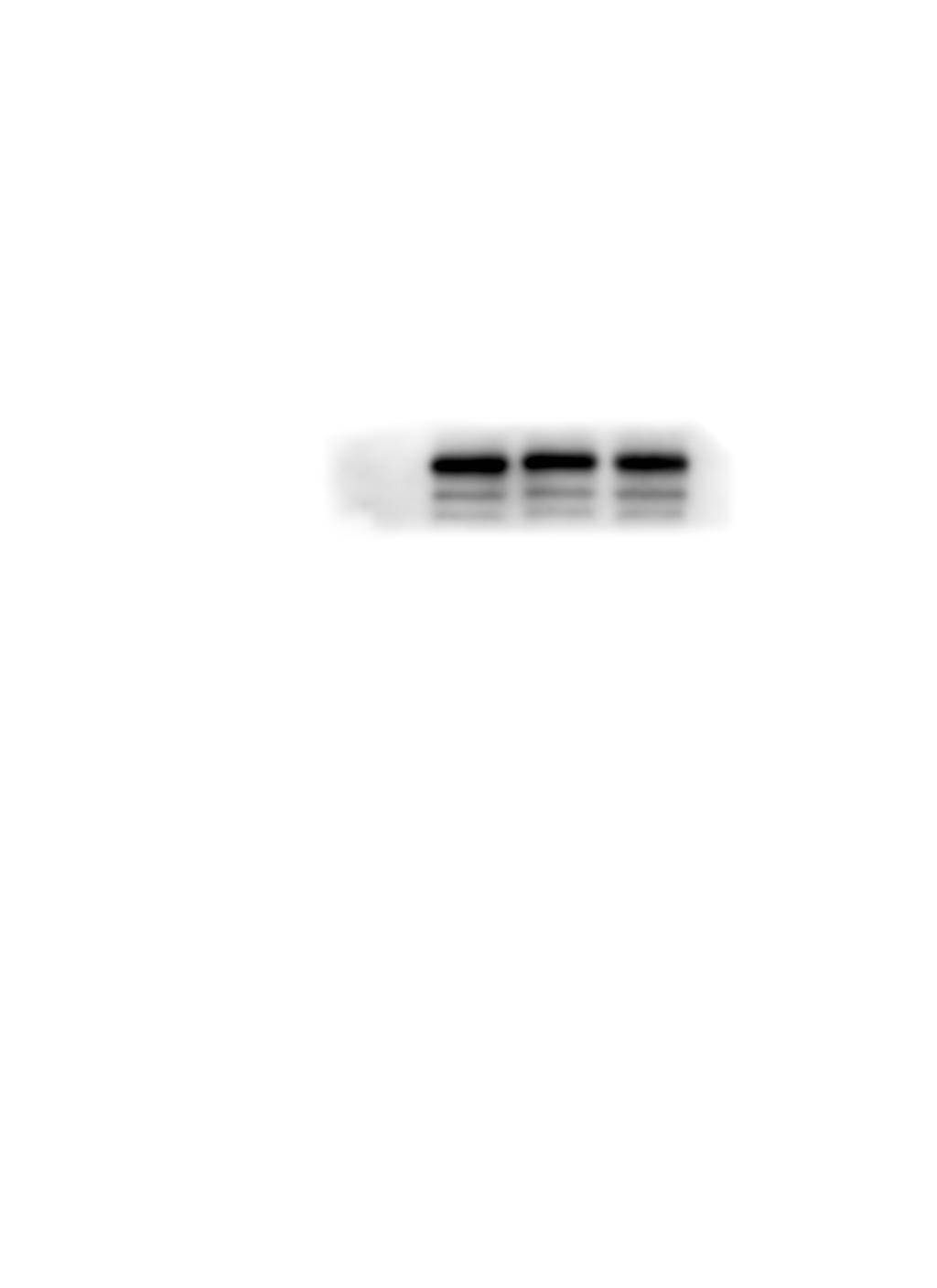

Supplement: Supplemental Information 2 — The CDF files for Fig. 2 are the data of analyzing the fingerprints of 10 batches of the rhizome of M. dauricum Polysaccharide. The software to open this file is the Similarity Evaluation System for Chromatographic Fingerprint of Traditional Chinese Medicine, and the software installation package is available in the Figure 2 folder. The OPJU file in the Figure 3 folder is the data of PCA analysis, and the open software is Origin, which can be downloaded and used for a fee from the Origin official website. [file peerj-10-13946-s002.zip › Raw date/Fig.6/p-P38/2/p-P38-GAPDH.jpg]

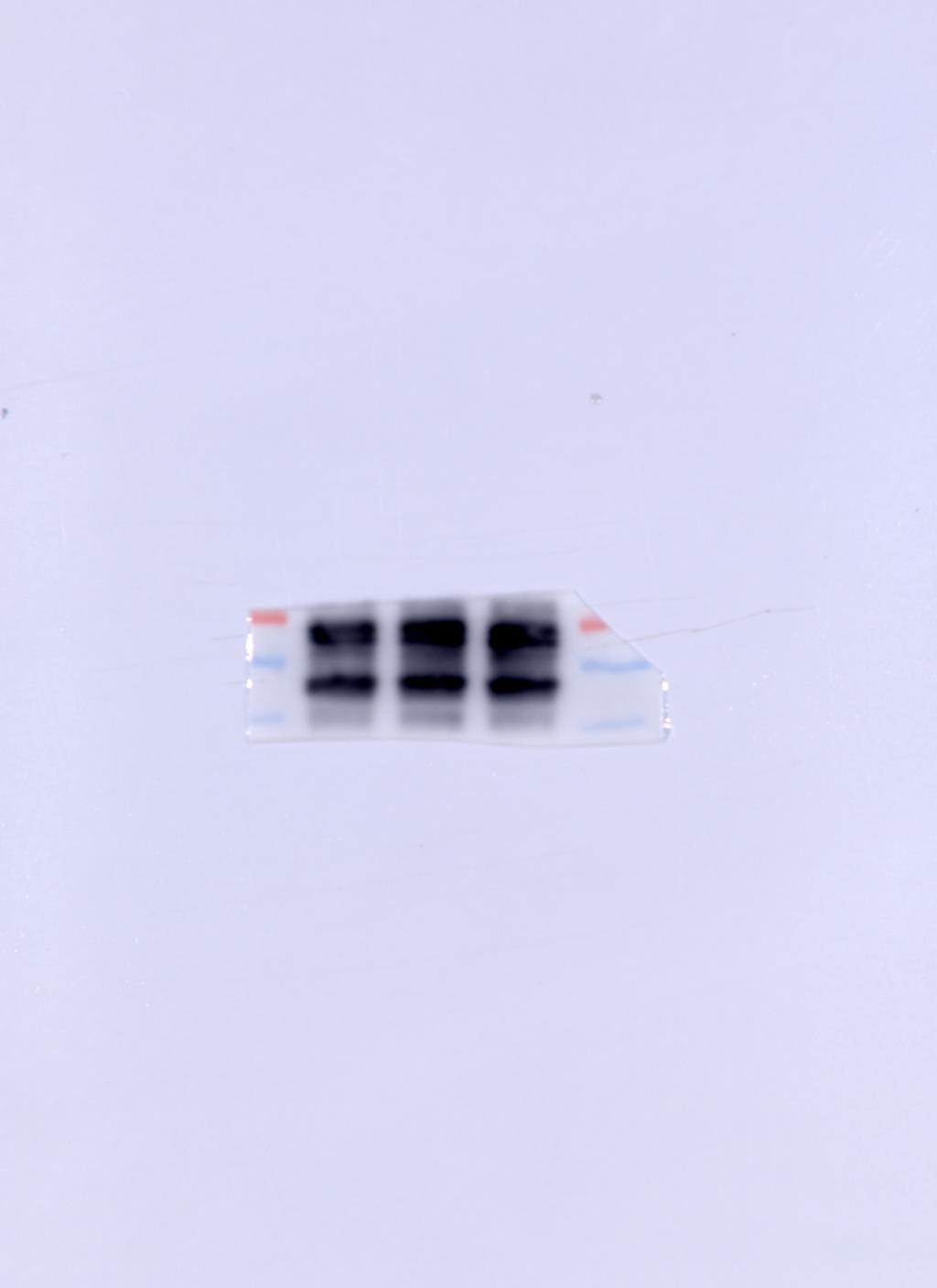

Supplement: Supplemental Information 2 — The CDF files for Fig. 2 are the data of analyzing the fingerprints of 10 batches of the rhizome of M. dauricum Polysaccharide. The software to open this file is the Similarity Evaluation System for Chromatographic Fingerprint of Traditional Chinese Medicine, and the software installation package is available in the Figure 2 folder. The OPJU file in the Figure 3 folder is the data of PCA analysis, and the open software is Origin, which can be downloaded and used for a fee from the Origin official website. [file peerj-10-13946-s002.zip › Raw date/Fig.6/p-P38/2/P38+Marker.jpg]

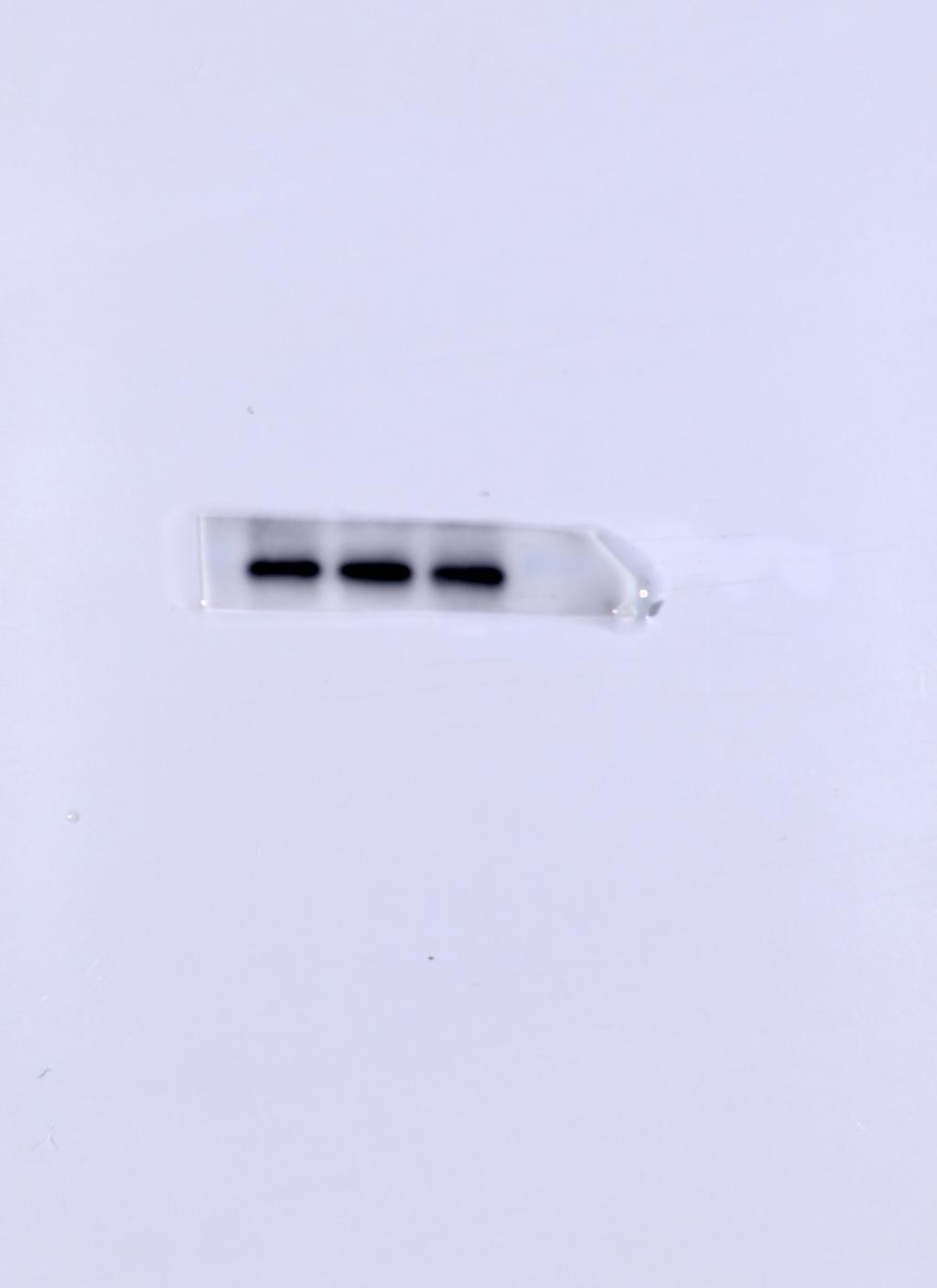

Supplement: Supplemental Information 2 — The CDF files for Fig. 2 are the data of analyzing the fingerprints of 10 batches of the rhizome of M. dauricum Polysaccharide. The software to open this file is the Similarity Evaluation System for Chromatographic Fingerprint of Traditional Chinese Medicine, and the software installation package is available in the Figure 2 folder. The OPJU file in the Figure 3 folder is the data of PCA analysis, and the open software is Origin, which can be downloaded and used for a fee from the Origin official website. [file peerj-10-13946-s002.zip › Raw date/Fig.6/p-P38/2/P38-GAPDH+Marker.jpg]

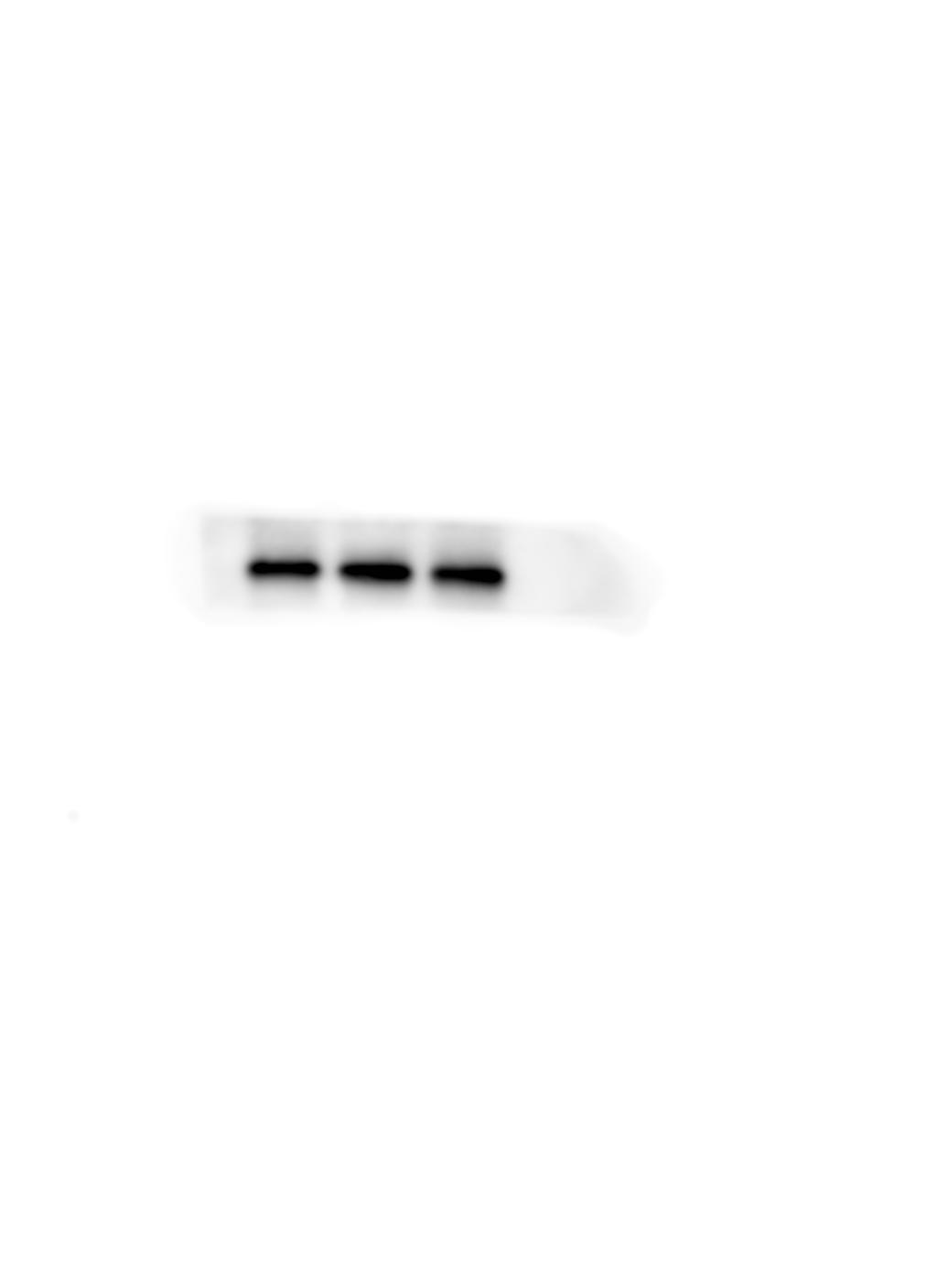

Supplement: Supplemental Information 2 — The CDF files for Fig. 2 are the data of analyzing the fingerprints of 10 batches of the rhizome of M. dauricum Polysaccharide. The software to open this file is the Similarity Evaluation System for Chromatographic Fingerprint of Traditional Chinese Medicine, and the software installation package is available in the Figure 2 folder. The OPJU file in the Figure 3 folder is the data of PCA analysis, and the open software is Origin, which can be downloaded and used for a fee from the Origin official website. [file peerj-10-13946-s002.zip › Raw date/Fig.6/p-P38/2/P38-GAPDH.jpg]

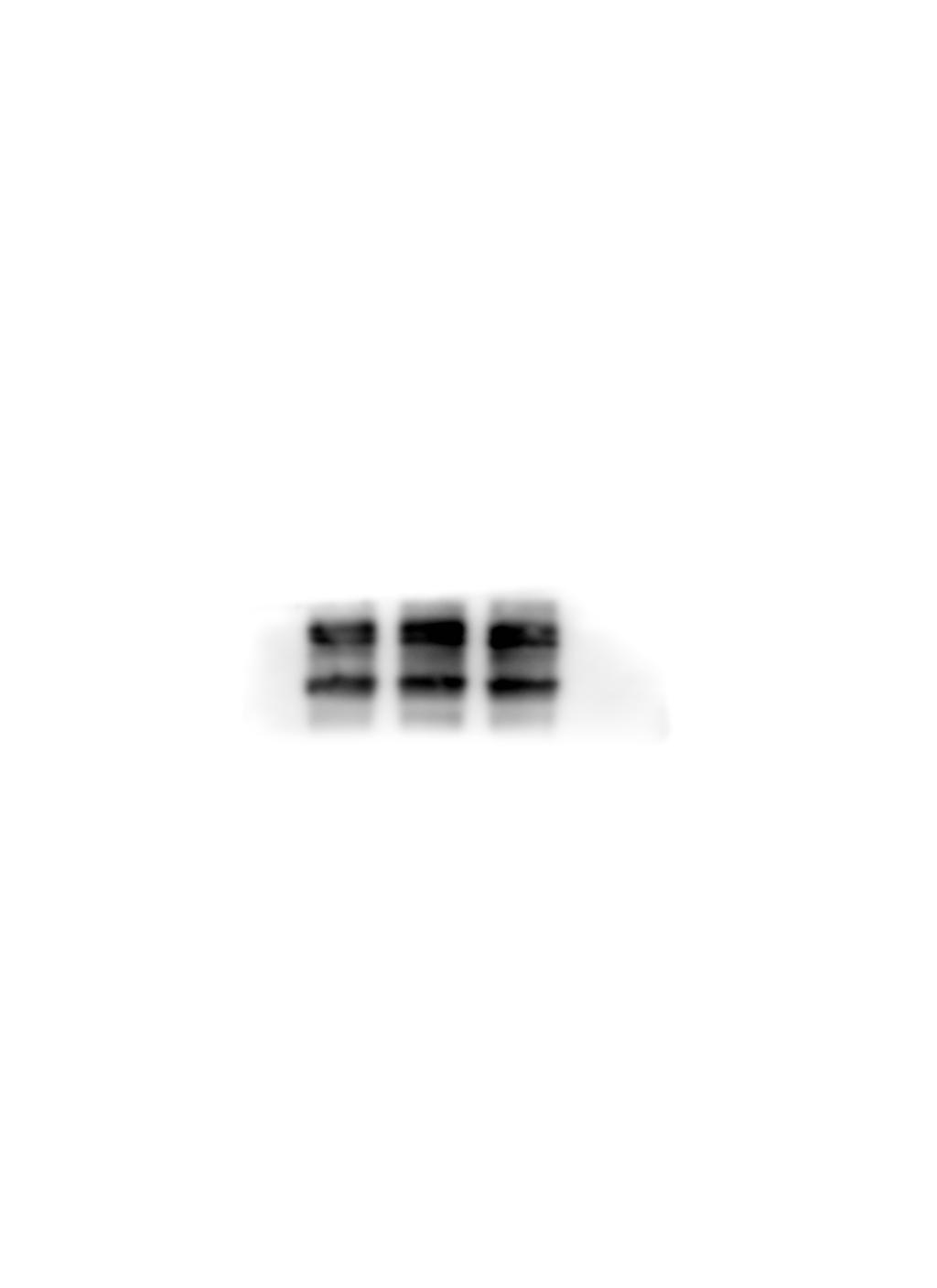

Supplement: Supplemental Information 2 — The CDF files for Fig. 2 are the data of analyzing the fingerprints of 10 batches of the rhizome of M. dauricum Polysaccharide. The software to open this file is the Similarity Evaluation System for Chromatographic Fingerprint of Traditional Chinese Medicine, and the software installation package is available in the Figure 2 folder. The OPJU file in the Figure 3 folder is the data of PCA analysis, and the open software is Origin, which can be downloaded and used for a fee from the Origin official website. [file peerj-10-13946-s002.zip › Raw date/Fig.6/p-P38/2/P38.jpg]
